# Single-cell Profiling Reveals Cooperative Participation of *SPP1^+^* Myeloid and *POSTN^+^* Fibroblast Subsets in Inflammation and Remodeling in Thoracic Aortic Aneurysm

**DOI:** 10.7150/ijms.124136

**Published:** 2026-03-04

**Authors:** Yingjiao Ju, Jingyi Yao, Song Zhang, Jiangxu Wu, Jiongao Xiang, Li Min, Mingyuan Liu

**Affiliations:** 1 Research Center, Beijing Friendship Hospital, Capital Medical University, Beijing 101300, China.; 2 Biobank, Beijing Friendship Hospital, Capital Medical University, Beijing 101300, China.; 3 Department of Vascular Surgery, Beijing Friendship Hospital, Capital Medical University, Beijing 100050, China.; 4 State Key Laboratory of Digestive Health, National Clinical Research Center for Digestive Diseases, Department of Gastroenterology, Beijing Friendship Hospital, Capital Medical University, Beijing 100050, China.

**Keywords:** Thoracic aortic aneurysm, single-cell RNA sequencing, myeloid-fibroblast crosstalk, SPP1/MK signaling, aortic wall remodeling, transcriptional regulation

## Abstract

Thoracic aortic aneurysm (TAA) is a life-threatening condition characterized by aortic dilation, inflammation, and extracellular matrix degradation. Despite advances in surgical management, effective pharmacological therapies are still lacking, largely due to an incomplete understanding of the cellular mechanisms driving disease progression. Although recent single-cell RNA sequencing (scRNA-seq) studies have revealed diverse cell types in TAA, the intercellular communication driving pathological remodeling is still poorly defined. Here, we performed integrated scRNA-seq analysis of human TAA (n = 8) and healthy aorta (n = 8) to construct a comprehensive cellular landscape. We identified a disease-associated crosstalk between *SPP1*^+^ myeloid cells and *POSTN*^+^ fibroblasts, mediated by SPP1 and MK signaling. These two cell subsets were enriched in TAA and co-activated TNF-α signaling via NF-κB and epithelial mesenchymal transition (EMT) pathways, thereby promoting inflammation and ECM remodeling. Cell-cell communication analysis further uncovered upregulated interactions involving SPP1-integrin (e.g., ITGAV/ITGA8/ITGA5+ITGB1) and MDK receptors (SDC4/SDC2/NCL/LRP1/ITGA4/ITGA6+ITGB1) in TAA. These computational findings were further supported by multiplex immunofluorescence and spatial transcriptomics analyses. By integrating key genes and signaling pathways, we identified hub genes and their associated transcription factors, whose regulatory activity was further supported by transcription factor regulon analysis. Our findings highlight the crucial role of myeloid-fibroblast interactions in driving TAA pathogenesis and identify potential therapeutic targets.

## Introduction

Thoracic Aortic Aneurysm (TAA) is a life-threatening cardiovascular disorder characterized by pathological aortic dilation, persistent inflammation and extracellular matrix degradation, which ultimately resulting in progressive structural compromise of the vascular wall^1^. TAA is often asymptomatic until rupture, which is associated with a mortality rate as high as 90%, making it a critical public health concern^2^, ^3^. Epidemiological studies have reported a significant increase in the global burden of TAA. From 2002 to 2014, the annual incidence nearly doubled, rising from 3.5 to 7.6 cases per 100,000 individuals^4^, and this upward trend continues^5^. Current clinical management of TAA primarily depends on open surgical repair involving resection and synthetic graft reconstruction, but this approach is limited by elevated perioperative rupture risk and frequent postoperative complications, including infection and thrombosis^6^. More importantly, the lack of effective pharmacological treatments highlights a significant gap in the non-surgical management of TAA. Therefore, elucidating the underlying cellular and molecular mechanisms, identifying therapeutic targets, and developing novel treatment strategies are of great significance for the prevention and treatment of TAA.

The pathological mechanisms underlying TAA are multifactorial, involving inflammatory responses, extracellular matrix (ECM) degradation, and aberrant aortic wall remodeling^1^. Although previous studies have demonstrated that both immune cells (e.g., macrophages) and non-immune cells (e.g., fibroblasts) contribute to disease progression^7^-^9^, the distinct characteristics of key cellular subsets and their interaction networks remain to be fully elucidated. Among these, myeloid cells, particularly macrophages play a central role in aortic wall remodeling and the formation of aortic dissection^10^, ^11^. Pro-inflammatory macrophages promote TAA progression by secreting matrix metalloproteinases (MMPs) and inflammatory cytokines, which induce ECM degradation and apoptosis of vascular smooth muscle cells^8^, ^12^. Adventitial fibroblasts, a major cellular component of the aortic wall^13^, actively contribute to aortic wall remodeling by migrating, proliferating, and producing collagen and matrix components during injury repair^14^. In TAA, medial vascular smooth muscle cells are markedly reduced, and fibroblasts become the predominant effector cells promoting aortic wall remodeling^15^.

Recent advances in scRNA-seq have substantially deepened our understanding of the cellular heterogeneity underlying TAA. However, most studies to date have primarily focused on characterizing VSMC and macrophage subpopulations, such as *Il1rn*^+^ or *Trem1*^+^ macrophages, or profiling global cellular composition, while the specific intercellular interactions between myeloid and fibroblast subsets have remained largely unexplored. For instance, Liu et al. delineated 20 distinct cellular subpopulations in bicuspid aortic valve (BAV)-associated TAA tissues, including various subtypes of VSMCs, fibroblasts, macrophages, and T lymphocytes, yet did not investigate intercellular communication networks in detail^16^. Liu et al. focused on macrophage heterogeneity and demonstrated that selective targeting of *Il1rn*^+^/*Trem*1^+^ macrophages significantly reduced aortic rupture in murine models^8^. In parallel, their investigation into VSMC phenotypic modulation revealed potential transcriptional regulators but did not address broader patterns of cell-cell communication^17^. Cao et al. further uncovered VSMC diversity in aneurysm pathogenesis^18^, while Wang et al. provided a broader cellular interaction map involving macrophages, endothelial cells, fibroblasts, and VSMCs in both healthy and diseased aortas, but did not specifically examine* SPP1*^+^ myeloid and *POSTN*^+^ fibroblasts^19^. Similarly, Chou et al. described the cellular diversity in sporadic ascending aortic aneurysms, concluding that disease progression is characterized by gene expression changes within established cellular lineages rather than the emergence of novel cell types^20^. Despite these important contributions, the mechanistic basis of myeloid-fibroblast interactions and their potential role in regulating chronic inflammation and ECM remodeling in TAA remains insufficiently understood.

Midkine (MDK, also known as MK) is a heparin-binding growth factor that plays pleiotropic roles in inflammation, tissue repair, and aortic wall remodeling^21^. It exerts its biological effects through interactions with several membrane receptors and co-receptors, including PTPRZ1, LRP1, ALK, integrin complexes, Notch2 and proteoglycans, thereby activating downstream signaling cascades including PI3K/AKT, NF-κB, and JAK/STAT pathways^22^. In cardiovascular diseases, MDK has been implicated in promoting vascular smooth muscle cell proliferation, macrophage recruitment, fibroblast activation, and extracellular matrix remodeling, contributing to the progression of atherosclerosis, post-ischaemic remodelling, and aortic wall pathology^23^-^25^. Given its dual role in orchestrating inflammatory and fibrotic responses, MK signaling may serve as a critical mediator of immune-stromal crosstalk within the aneurysmal microenvironment, potentially linking myeloid derived inflammatory cues with fibroblast driven aortic wall remodeling.

In this study, publicly available scRNA-seq data were integrated to construct a comprehensive cellular landscape of TAA and healthy aortic tissues, enabling a systematic characterization of the functional properties of myeloid and fibroblast subpopulations and their disease-associated alterations. Notably, *SPP1^+^* myeloid cells and *POSTN^+^* fibroblasts were found to cooperatively promote inflammation and ECM remodeling via SPP1/MK signaling-mediated ligand-receptor interactions, thereby exacerbating structural damage to the aortic wall. Further analysis identified potential hub genes and associated transcription factors that are linked to key molecular signatures and signaling pathways. These findings not only enhance our understanding of TAA pathogenesis but also lay a theoretical foundation for the development of targeted therapeutic strategies.

## Materials and methods

### scRNA-seq data acquisition and processing

Raw scRNA-seq data were obtained from the SRA database (PRJNA919181/GSE222318 and PRJNA649846/GSE155468), comprising 8 healthy control and 8 TAA samples. Data preprocessing was performed using the Cell Ranger count pipeline (v8.0.1) with alignment to the GRCh38 human reference genome. Potential doublets were identified and removed using Scrublet (expected doublet rate: 0.06)^26^. Low-quality cells were filtered out using Seurat (v4.0)^27^ based on the following criteria: UMI counts <1,000 or >50,000, detected genes <300 or >5,000, mitochondrial gene content >10%, and Scrublet-flagged doublets. Batch effects across samples were corrected using Harmony (v1.2.3)^28^, based on the top 30 principal components (PCs). The integrated dataset was then subjected to dimensionality reduction via UMAP and unsupervised clustering (30 PCs, resolution = 0.6). Cell types were manually annotated based on known canonical markers in combination with cluster-specific marker genes (Benjamini-Hochberg adjusted *P* values < 0.05). Major cell populations were further subclustered using the 30 PCs with a lower resolution parameter (resolution = 0.1). Subpopulations were named by combining cluster labels with the top differentially expressed genes (based on the highest log2 fold change). Hub gene score was calculated using the AddModuleScore function.

### Differential gene expression analysis, functional annotation, and GSEA

Differentially expressed genes (DEGs) were identified using the FindAllMarkers function in the Seurat package. Genes expressed in at least 10% of cells within each cluster and with a log2 fold change greater than 0.25 were selected for further analysis. Genes with adjusted *P* values less than 0.05 were considered statistically significant. Among these, genes with an average log2 fold change greater than 0 were regarded as upregulated. Functional enrichment analysis of DEGs was performed using the enricher function in the clusterProfiler package (v4.6.2)^29^, with Hallmark gene sets (category “H”) from the msigdbr package (v10.0.1)^30^ serving as the reference background. Gene Set Enrichment Analysis (GSEA) was also carried out using the clusterProfiler package with the same Hallmark gene sets from the MsigDB database Pathways with Benjamini-Hochberg (BH) adjusted *P* values below 0.05 were considered significantly enriched. Gene Set Variation Analysis (GSVA) was conducted using the GSVA package (v1.46.0)^31^, based on the average gene expression within each cell cluster. The GSVA scores were calculated using the "gsva" method with a Poisson distribution kernel.

### Cell communication pseudotime and transcription factor prediction

Intercellular communications were analyzed using the CellChat package (v1.6.1)^32^, classifying interaction types into “Secreted Signaling”. The single-cell pseudotime differentiation trajectory was constructed using the monocle package (v2.34.0)^33^. Transcription factor prediction for single-cell subpopulations was performed using dorothea (v1.10.0)^34^, with downsampling of 100 cells for calculation. Regulon activity was normalized using the scale method, and each regulon contained at least four target genes. Cell cluster-specific activated transcription factors were identified using the FindAllMarkers function. Only upregulated genes were selected, which were expressed in at least 25% of cells within the cluster and had a log2 fold change greater than 0.25. Transcription factors for hub genes were further predicted using the TRRUST database^35^.

### Transcription factor regulon inference and activity analysis

Motif enrichment and regulon activity analyses were performed using the Python implementation of SCENIC (pySCENIC v0.12.1)^36^. The normalized single-cell expression matrix was converted to a loom file and used as input for downstream analyses. Gene regulatory networks were inferred using GRNBoost2 with candidate regulators restricted to a curated list of human transcription factors. Regulons were subsequently defined using the cisTarget module implemented in pySCENIC, based on the hg38 RefSeq-r80 cisTarget motif ranking database with a ±10 kb window around transcription start sites (hg38__refseq-r80__10kb_up_and_down_tss.mc9nr.genes_vs_motifs.rankings.feather). Regulon activity was quantified at the single-cell level using AUCell by assessing the enrichment of regulon target genes within each cell's expressed gene set. Regulon activity scores were compared across cell subtypes and disease conditions, with downstream interpretation focusing on eight transcription factors prioritized based on dorothea and TRRUST predictions (*POU5F1*, *POU2F1*, *HOXD3*, *PAX6*, *TWIST2*, *JUND*, *CEBPA*, and *SP1*).

### Multiplex immunofluorescence staining and quantitative image analysis

Multiplex immunofluorescence staining was performed on paraffin-embedded aortic tissue sections to validate the spatial association between *SPP1*^+^ myeloid cells and *POSTN*^+^ fibroblasts. Tissue sections (4-5 μm) were deparaffinized, rehydrated, and subjected to heat-induced antigen retrieval using citrate buffer (pH 6.0). Endogenous peroxidase activity was quenched with 3% hydrogen peroxide, followed by blocking with normal serum. Sections were incubated overnight at 4 °C with primary antibodies against CD68 (AF20022, AiFang Biological, 1:1000 dilution), SPP1 (AFRP0012, AiFang Biological, 1:500 dilution), α-SMA (AFMM0002, AiFang Biological, 1:1000 dilution), and POSTN (AFRM0263, AiFang Biological, 1:1000 dilution). HRP-conjugated polymer secondary antibodies were applied, and signal detection was achieved using tyramide signal amplification (TSA) with fluorophore-labeled tyramides. Antibody stripping by heat-induced antigen retrieval was performed between staining cycles to enable multiplex labeling. Nuclei were counterstained with DAPI. Fluorescence images were acquired using a multispectral fluorescence microscope under identical acquisition settings, and image processing was performed using KFSlideOS software.

Quantitative analysis of mIF images was performed in QuPath (v0.5.1). Cells were segmented using a StarDist-based workflow. A supervised machine-learning object classifier was trained on representative regions to classify cell phenotypes and identify double-positive populations. For each group, 15 randomly selected ROIs (150 μm × 150 μm) were annotated and analyzed. In each ROI, the proportions of CD68^+^SPP1^+^ and α-SMA^+^POSTN^+^ double-positive cells were calculated as the number of double-positive cells divided by the total number of detected DAPI^+^ cells. In addition, mean fluorescence intensity (MFI) of SPP1 and POSTN was quantified within the corresponding double-positive cells and normalized to DAPI intensity in the same ROI (SPP1/DAPI and POSTN/DAPI). For group comparisons of double-positive cell proportions, counts were pooled within each group and assessed using Fisher's exact test (given the limited number of sections for mIF validation). DAPI-normalized MFI (SPP1/DAPI and POSTN/DAPI) was compared between groups at the ROI level using a two-sided Mann-Whitney U test (Wilcoxon rank-sum).

### Spatial Transcriptomics Analysis

Spatial transcriptomics (ST) data were processed and analyzed using the Seurat (v4.0) framework. To enable accurate cell type annotation of ST spots, a curated scRNA-seq reference was constructed by integrating a comprehensive single cell dataset. Cells were grouped according to their annotated cell types, and approximately 300 cells were randomly subsampled from each group to generate a balanced reference. Both the reference dataset and the ST dataset were normalized using SCTransform, with 3,000 variable features retained. SCT normalized assays were used for all downstream integration and label transfer steps. Cell type labels and associated prediction scores were transferred to ST spots using an anchor-based approach implemented in FindTransferAnchors and TransferData with 30 dimensions. Transferred annotations were stored in the metadata of the ST object for subsequent analyses.

Spatial colocalization between predicted cell type programs was assessed using a k-nearest-neighbor (kNN) based framework. Spatial coordinates for each spot were obtained using GetTissueCoordinates, and the k nearest neighbors (k = 3, 4, 5, 6, 8, 10, 12, and 15) were identified using the FNN algorithm. Given two cell type-specific prediction scores (*A* and *B*), a continuous colocalization metric was defined as:



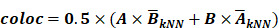



where 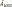
and 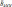
denote the mean values of *A* and *B* among the k nearest spatial neighbors, respectively. Spots with *coloc* > 0 were classified as colocalization positive. A k sensitivity analysis was performed to assess robustness across spatial scales. Group differences in the frequency of colocalization positive spots were evaluated using one-sided Fisher's exact tests, and odds ratios were calculated.

## Results

### Cell type characteristics of human ascending aorta reveal pivotal roles of myeloid and fibroblast cells

**Figure 1:**
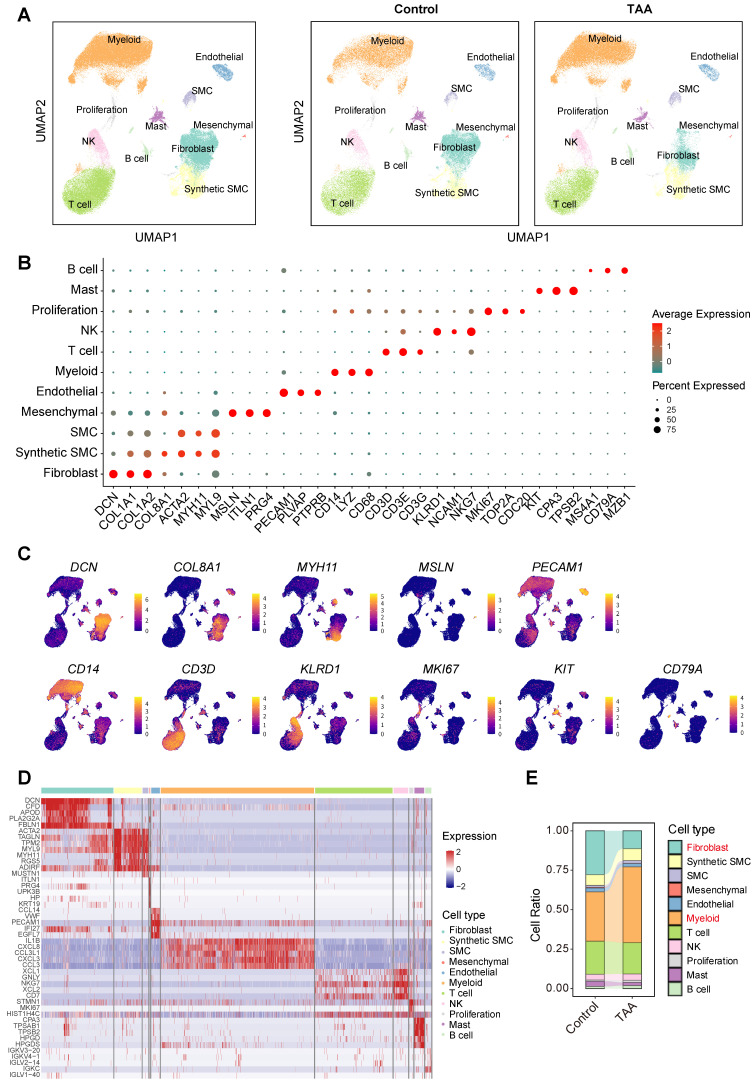
** Single-cell transcriptomic profiling of 120,057 cells from control and TAA groups. A,** UMAP plot on the left displays the integrated dataset, with cells colored by 11 major cell types. The UMAP plots on the right show the distribution and composition of these major cell types in each group.** B**, Dot plot showing the expression of classical marker genes used to define each major cell type. **C,** UMAP plots illustrating the spatial expression patterns of representative marker genes.** D**, Heatmap showing the top five marker genes for each major cell type.** E**, Stacked bar plot comparing the proportions of each major cell type between control and TAA groups.

To construct a comprehensive and quantitatively matched cellular landscape of the human ascending aorta, we integrated publicly available scRNA-seq datasets from TAA patients (n = 8) and healthy controls (n = 8), specifically PRJNA919181 (GSE222318) and PRJNA649846 (GSE155468). We employed the Harmony algorithm to correct for potential batch effects between these two distinct datasets (Supplementary Figure 1). Following stringent quality control batch effect correction, and normalization, we obtained a total of 120,057 high-quality cells, including 55,337 from healthy individuals and 64,720 from TAA patients. Data from all 16 samples were integrated using the Seurat framework. Unsupervised clustering based on canonical marker expression identified 11 major cell types (Figure 1A). These included fibroblasts (*DCN*, *COL1A1*, *COL1A2*; n = 22,743), smooth muscle cells (SMCs; *MYH11*, *ACTA2*, *MYL9*; n = 8,637), synthetic SMCs (*DCN*, *COL8A1*, *ACTA2*; n = 1,831), mesenchymal cells (*MSLN*, *ITLN1*, *PRG4*; n = 258), endothelial cells (*PECAM1*, *PLVAP*, *PTPRB*; n = 2,738), myeloid cells (*CD14*, *LYZ*, *CD68*; n = 48,377), T cells (*CD3D*, *CD3E*, *CD3G*; n = 24,469), natural killer (NK) cells (*KLRD1*, *NCAM1*, *NKG7*; n = 4,618), proliferating cells (*MKI67*, *TOP2A*, *CDC20*; n = 1,337), mast cells (*KIT*, *CPA3*, *TPSB2*; n = 3,122), and B cells (*MS4A1*, *CD79A*, *MZB1*; n = 1,927) (Figure 1B-C). Representative molecular signatures for each cell type are presented in Figure 1D. Among these, myeloid cells, T cells, NK cells, proliferating cells, mast cells, and B cells were grouped as immune populations, while fibroblasts, SMCs, synthetic SMCs, mesenchymal cells, and endothelial cells were classified as non-immune populations.

To investigate potential population shifts associated with disease, we compared cell distributions by separating the control and TAA groups in the UMAP visualizations (Figure 1A). Although only fibroblasts exhibited statistically significant changes between conditions, markedly declining in TAA tissues, myeloid cells showed a notable upward trend (Figure 1E, Supplementary Figure 2), consistent with prior studies implicating immune infiltration in TAA progression^7^, ^8^. The reduction in fibroblast populations aligns with previous reports of extracellular matrix degradation and aortic wall remodeling during aneurysm development^15^. Together, these compositional changes highlight myeloid cells and fibroblasts as key cellular contributors to TAA pathogenesis, guiding our subsequent analyses toward these pivotal cell types.

### Pro-inflammatory functions of myeloid cells play a crucial role in TAA pathogenesis

**Figure 2:**
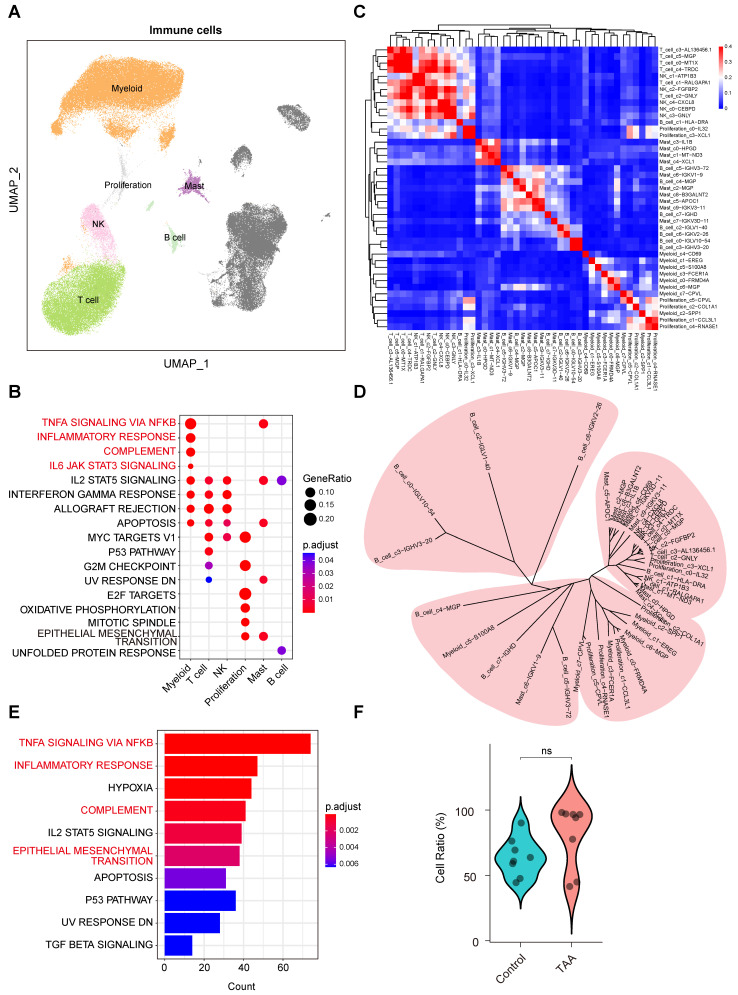
** Molecular characteristics of immune cells in human ascending aortic tissue. A**, UMAP plots showing the distribution of immune cells. **B**, Hallmark pathway enrichment analysis across immune cell types. Marker color and size represent the adjusted *P* value and gene ratio, respectively. Adjusted *P* values were calculated using the BH method. **C**, Unsupervised hierarchical clustering of the average gene expression for the top 1000 variable genes among immune subpopulations, illustrating the correlation distance and relatedness between clusters. **D**, Unsupervised hierarchical clustering of the top 1,000 most variable genes from each immune subcluster reveals the transcriptional relatedness among distinct cell lineages. **E**, Bar plot showing hallmark enrichment of differentially expressed genes between TAA and control immune cells. Adjusted *P* values were calculated using the BH method, and marker color indicates the adjusted *P* value. **F**, Violin plots showing a trend toward higher levels of immune cells in the TAA group compared to the control group, although the difference was not statistically significant. Statistical analysis was performed using the Wilcoxon rank-sum test.

To elucidate the molecular characteristics of immune cells within the thoracic aortic wall, we focused our analysis on immune cell populations, including myeloid cells, T cells, NK cells, proliferating cells, mast cells, and B cells (Figure 2A). Functional enrichment analysis revealed that myeloid cells were predominantly associated with pro-inflammatory responses. Key enriched pathways included TNF-α signaling via NF-κB, the inflammatory response, complement, and IL6-JAK-STAT3 signaling (Figure 2B).

To further dissect immune cell heterogeneity, we performed integrative unsupervised reclustering of all immune cells, identifying 43 distinct subpopulations (Figure 2C). These subtypes were annotated based on their unique gene expression profiles (Supplementary Figure 3A). Based on DEGs within each immune cell subset, we assessed transcriptional similarities among the subtypes. While most subtypes within the same major immune population clustered closely, further supporting the accuracy of our cell-type annotations. Interestingly, we also observed cross-lineage similarities among certain subpopulations (Figure 2C-D), suggesting that these subpopulations may be responding to similar stimuli, resulting in comparable transcriptional profiles.

Comparative analysis of immune cells between TAA and control samples revealed that DEGs were predominantly enriched in TNF-α signaling via NF-κB, inflammatory response, and complement pathways (Figure 2E), consistent with the known pro-inflammatory roles of myeloid cells, even though cell proportions showed minimal differences between the groups (Figure 2F). Strikingly, we also identified significant enrichment of the EMT pathway (Figure 2E), suggesting a pathogenic synergy: infiltrating myeloid cells may secrete effector molecules (e.g., proteases, cytokines) that act in concert with EMT-transformed non-immune cells. In this context, myeloid cells facilitate extracellular matrix degradation, while EMT-activated non-immune cells may promote maladaptive aortic remodeling, together driving progressive weakening and dilation of the vessel wall in TAA. Coupled with the observed expansion of myeloid populations in TAA tissues, these findings underscore the pivotal role of pro-inflammatory myeloid cells in driving TAA pathogenesis.

### Fibroblasts exhibit both remodeling and pro-inflammatory characteristics

**Figure 3:**
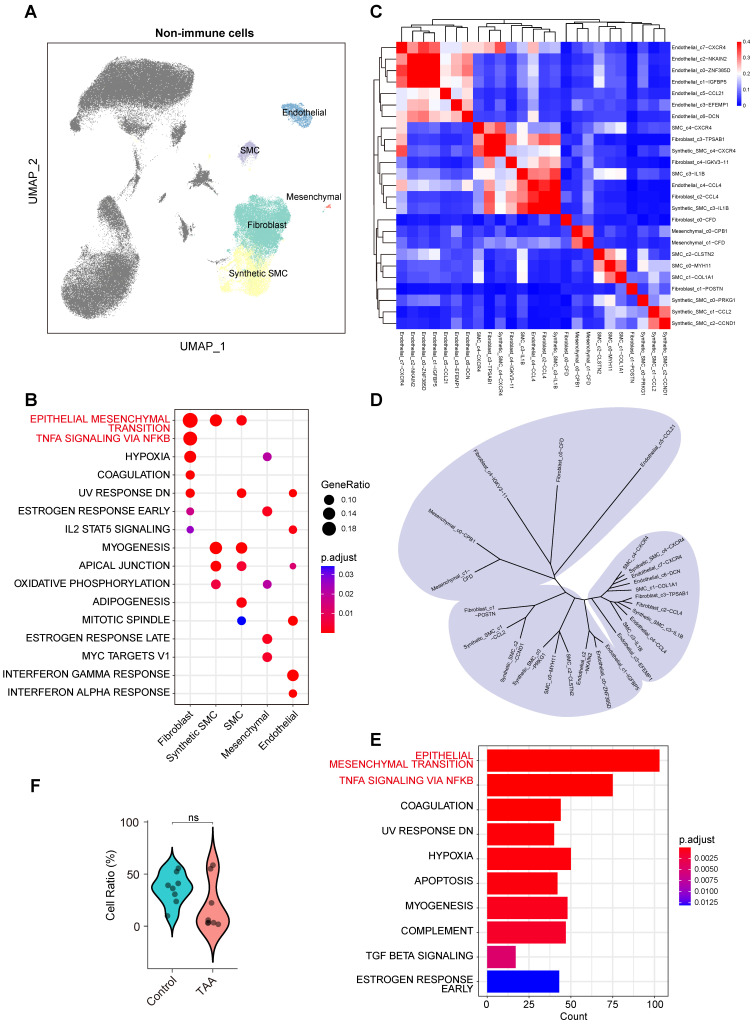
** Molecular characteristics of non-immune cells in human ascending aortic tissue. A**, UMAP plots showing the distribution of non-immune cells. **B**, Hallmark pathway enrichment analysis across non-immune cell types. Marker color and size represent the adjusted *P* value and gene ratio, respectively. Adjusted *P* values were calculated using the BH method. **C**, Unsupervised hierarchical clustering of the average gene expression for the top 1000 variable genes among non-immune subpopulations, illustrating the correlation distance and relatedness between clusters. **D**, Unsupervised hierarchical clustering of the top 1,000 most variable genes from each non immune subcluster reveals the transcriptional relatedness among distinct cell lineages. **E**, Bar plot showing hallmark enrichment of differentially expressed genes between TAA and control non-immune cells. Adjusted *P* values were calculated using the BH method, and marker color indicates the adjusted *P* value. **F**, Violin plots showing a trend toward lower levels of non-immune cells in the TAA group compared to the Control group, although the difference was not statistically significant. Statistical analysis was performed using the Wilcoxon rank-sum test.

Non-immune cells play essential roles in maintaining the structural and functional integrity of the aortic wall. We conducted an in-depth analysis of non-immune populations, including fibroblasts, synthetic SMCs, contractile SMCs, mesenchymal cells, and endothelial cells (Figure 3A), and found that fibroblasts exhibited notable both remodeling and pro-inflammatory characteristics. Functional enrichment analysis revealed significant enrichment of the EMT pathway in fibroblasts, synthetic SMCs, and SMCs (Figure 3B), indicating their involvement in aortic wall remodeling processes. Remarkably, fibroblasts also showed significant enrichment in the TNF-α signaling via NF-κB pathway, a characteristic feature of inflammatory activation, despite their classification as non-immune cells.

To further dissect cellular heterogeneity, we performed reclustering and reannotation of non-immune cells (Supplementary Figure 3B), resulting in the identification of 25 distinct subpopulations. DEG-based similarity analysis (Figure 3C) revealed considerable cross-lineage relationships, especially among fibroblast subsets (Figure 3D), suggesting functional diversity within this cell type. For example, the Fibroblast_c0-*CFD* subpopulation clustered closely with mesenchymal cells, whereas Fibroblast_c1-*POSTN* was more closely aligned with SMCs and synthetic SMCs, indicating that fibroblasts may exhibit functional diversity driven by distinct regulatory mechanisms or activation state.

Differential analysis between control and TAA samples confirmed that the EMT pathway was the most significantly altered across non-immune populations (Figure 3E), reflecting disruption of vascular structure in disease, despite no significant differences in cell proportions between the groups (Figure 3F). Notably, the TNF-α signaling via NF-κB pathway was also prominently enriched in the TAA group (Figure 3E). Together with the observed transcriptional profile of fibroblasts, these findings underscore their central role in TAA pathogenesis, not only as structural components but also as active participants in inflammatory processes.

### Coordinated but cell type-specific pathway activation across immune and stromal populations

Our preceding analyses revealed that key signaling pathways, including TNF-α signaling via NF-κB and EMT, were significantly enriched across diverse cell types spanning both immune (for example, myeloid cells) and stromal (for example, fibroblasts) lineages (Figures 2B, and 2E, 3B, and 3E, Supplementary Figure 4A). To determine whether this broad enrichment was driven by a common set of genes or by distinct, cell type-specific modules, we performed Jaccard similarity analysis of the gene sets constituting these pathways across cell populations.

The analysis demonstrated limited overlap among the underlying gene modules. For the TNF-α via NF-κB signaling pathway, the gene set similarity between immune (myeloid and mast) cells and stromal fibroblasts was moderate (Jaccard index = 0.33) (Supplementary Figure 4B), indicating partially shared but largely distinct transcriptional programs. EMT-related genes showed higher similarity among stromal subtypes (SMC and synthetic SMC; Jaccard index = 0.33) but minimal overlap with immune clusters (Jaccard index < 0.25) (Supplementary Figure 4C). These results suggest that the execution of these broadly enriched biological processes is highly dependent on cellular lineage.

Together, these findings highlight a coordinated but specialized pattern of pathway activation across immune and stromal compartments in TAA. Although immune and stromal cells exhibit simultaneous activation within the diseased microenvironment, they rely on distinct molecular programs to mediate inflammation and aortic wall remodeling. This integrated pattern of coordinated but cell type-specific pathway activation reveals a well-orchestrated functional interplay among distinct cell populations that collectively drive TAA progression.

### Cell-cell communication reveals myeloid-fibroblast interactions mediated by SPP1 and MK signaling pathways

**Figure 4:**
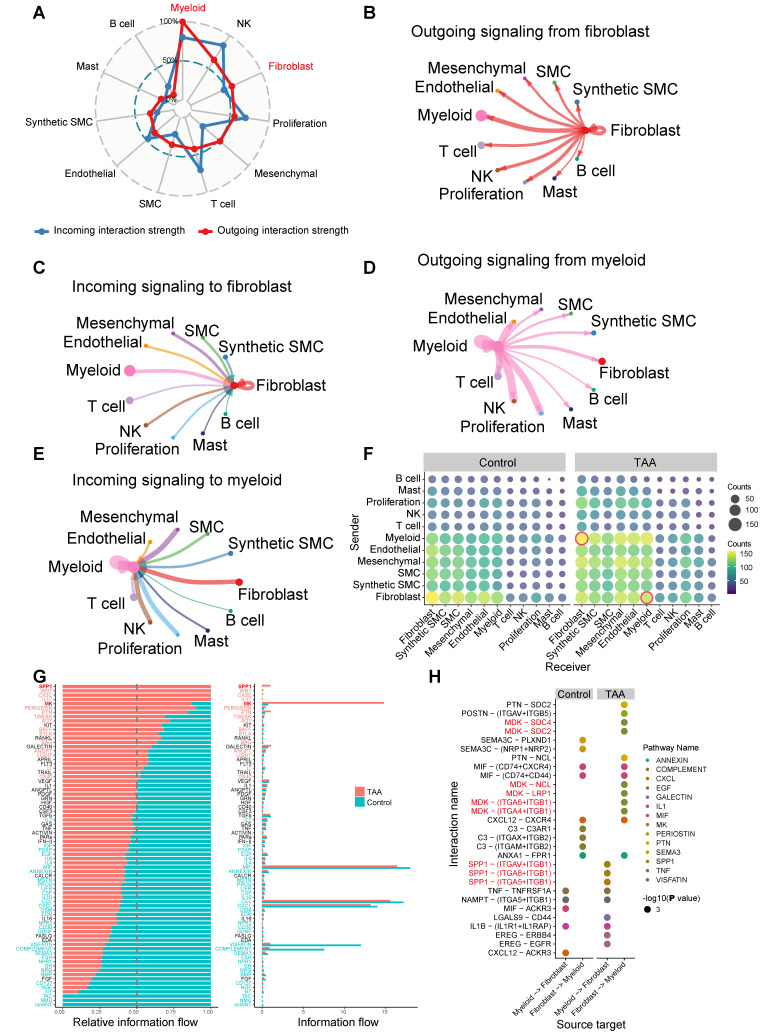
** Cell-cell communication between 11 major cell types. A**, Radar plot displaying intercellular communication patterns among the 11 major cell types. Outgoing and incoming signaling are represented by red and blue lines, respectively. **B**, Outgoing signaling from fibroblasts to the other ten major cell types. **C**, Incoming signaling to fibroblasts from the other ten major cell types. **D**, Outgoing signaling from myeloid cells to the other ten major cell types. **E**, Incoming signaling to myeloid cells from the other ten major cell types.** F**, A bubble plot displays the number of cell-cell communication interactions among 11 major cell types in the Control and TAA groups. Each bubble represents the interaction count between a pair of cell types, with size and color corresponding to the number of interactions. Interactions between myeloid cells and fibroblasts are highlighted with red circles.** G**, Cell-cell communication analysis identifying significantly upregulated signaling pathways in the TAA group. **H**, Disease-specific ligand-receptor pairs between myeloid and fibroblast populations. Circle size represents interaction significance (-log₁₀[*P* value]), while color indicates the different pathways for ligand-receptor pairs.

Building upon our findings that identified myeloid cells and fibroblasts as key contributors to TAA pathogenesis, we employed CellChat to systematically explore intercellular communication networks. Our analysis revealed that fibroblasts and myeloid cells function as central hubs in the signaling landscape of the aortic wall (Figure 4A-E). Notably, fibroblasts exhibited the strongest outgoing signaling toward myeloid cells and also received the most intense incoming signals from them, highlighting their central role in intercellular communication (Figure 4B-C). Myeloid cells, in turn, demonstrated robust bidirectional communication with all immune cell populations (Figure 4D-E), and their increased proportion in TAA further supports the presence of extensive immune infiltration. Notably, among non-immune cells, fibroblasts showed the strongest bidirectional signaling interactions with myeloid cells (Figure 4D-E). Importantly, disease-specific comparison revealed a marked enhancement in myeloid-fibroblast communication in TAA samples relative to controls (Figure 4F), emphasizing a potential cooperative interaction that contributes to disease progression. Differential communication analysis identified the SPP1 and MK pathways as the most significantly upregulated signaling axes in TAA (Figure 4G). Among these, myeloid-to-fibroblast interactions were primarily mediated by disease-enriched ligand-receptor pairs, including SPP1-(ITGAV+ITGB1), SPP1-(ITGA8+ITGB1), and SPP1-(ITGA5+ITGB1) (Figure 4H), suggesting that SPP1 signaling from myeloid cells plays a crucial role in activating fibroblasts during TAA. Fibroblast-to-myeloid communication in the disease group revealed activation of the MK signaling pathway through multiple ligand-receptor pairs, including MDK-SDC4, MDK-SDC2, MDK-NCL, MDK-LRP1, MDK-(ITGA6+ITGB1), and MDK-(ITGA4+ITGB1) (Figure 4H).

**Figure 5:**
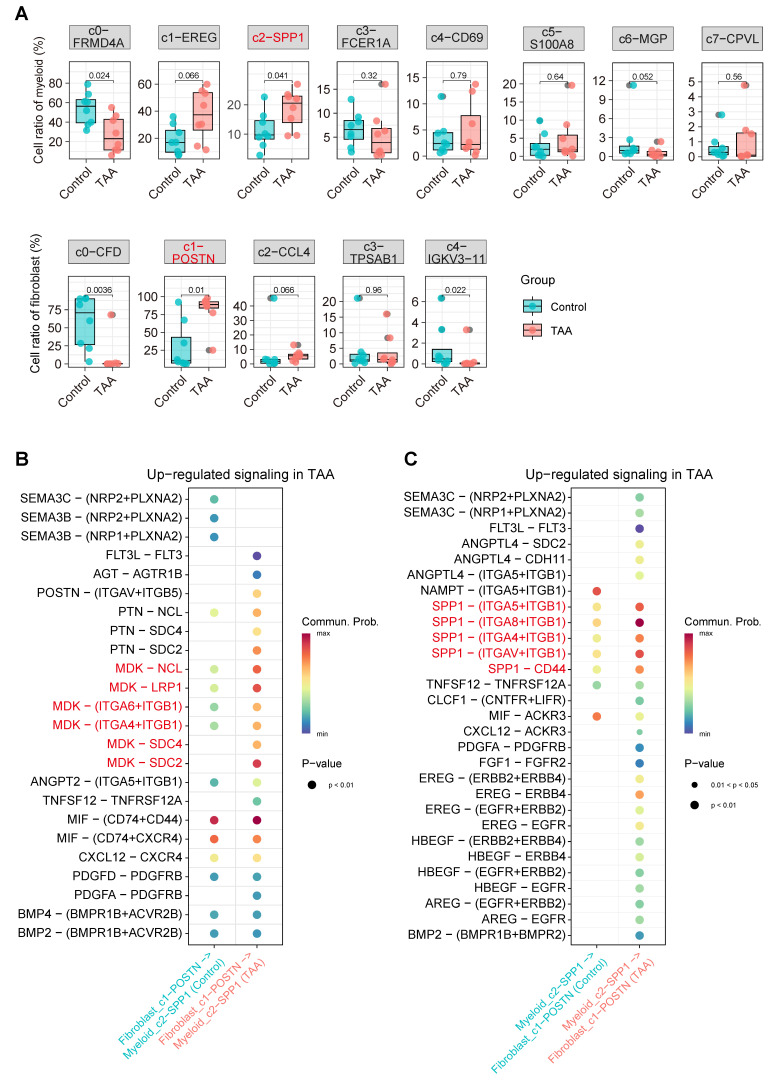
** Disease associated changes in myeloid and fibroblast subpopulations and their intercellular signaling. A**, Boxplot comparing the proportional differences of myeloid and fibroblast subclusters between TAA (red) and control (blue) groups. Statistical analysis was performed using the Wilcoxon rank-sum test. **B**, Significantly enhanced ligand-receptor signaling from fibroblast_c1-*POSTN* to myeloid_c2-*SPP1* in the TAA group. **C**, Increased ligand-receptor signaling from myeloid_c2-*SPP1* to fibroblast_c1-*POSTN* in the TAA group.

Detailed analysis of SPP1 signaling identified the *SPP1*^+^ myeloid subset Myeloid_c2-*SPP1* as the predominant source of this pathway (Supplementary Figure 5A-B). Interestingly, in the TAA group, the Myeloid_c1-*EREG* subset also partially contributed to SPP1 signal output, further amplifying pathway activity. For the MK signaling pathway, the primary sources of signaling were the *CFD^+^* and *POSTN^+^* fibroblast subsets, namely Fibroblast_c0-*CFD* and Fibroblast_c1-*POSTN* (Supplementary Figure 5C-D). In the TAA group, signal output from Fibroblast_c0-*CFD* was reduced, whereas Fibroblast_c1-*POSTN* maintained a strong signaling contribution. Notably, Fibroblast_c2-*CCL4* and Fibroblast_c3-*TPSAB1* displayed a striking increase in MK pathway signal output. Importantly, both Myeloid_c2-*SPP1* and Fibroblast_c1-*POSTN* subpopulations showed proportional significantly increases in TAA samples (Figure 5A), along with significantly enhanced intercellular communication (Figure 5B-C). These findings collectively demonstrate that Myeloid_c2-*SPP1* and Fibroblast_c1-*POSTN* subsets enhance mutual interactions through the SPP1 and MK signaling pathways, respectively, thereby actively contributing to TAA pathogenesis.

### Myeloid_c2-*SPP1* and Fibroblast_c1-*POSTN* may form “a positive feedback loop” that exacerbates vascular wall damage in TAA

To elucidate the biological roles of Myeloid_c2-*SPP1* and Fibroblast_c1-*POSTN* in TAA, we performed detailed molecular characterization, revealing that these subpopulations possess distinct yet synergistic functional signatures and form coordinated regulatory networks.

**Figure 6:**
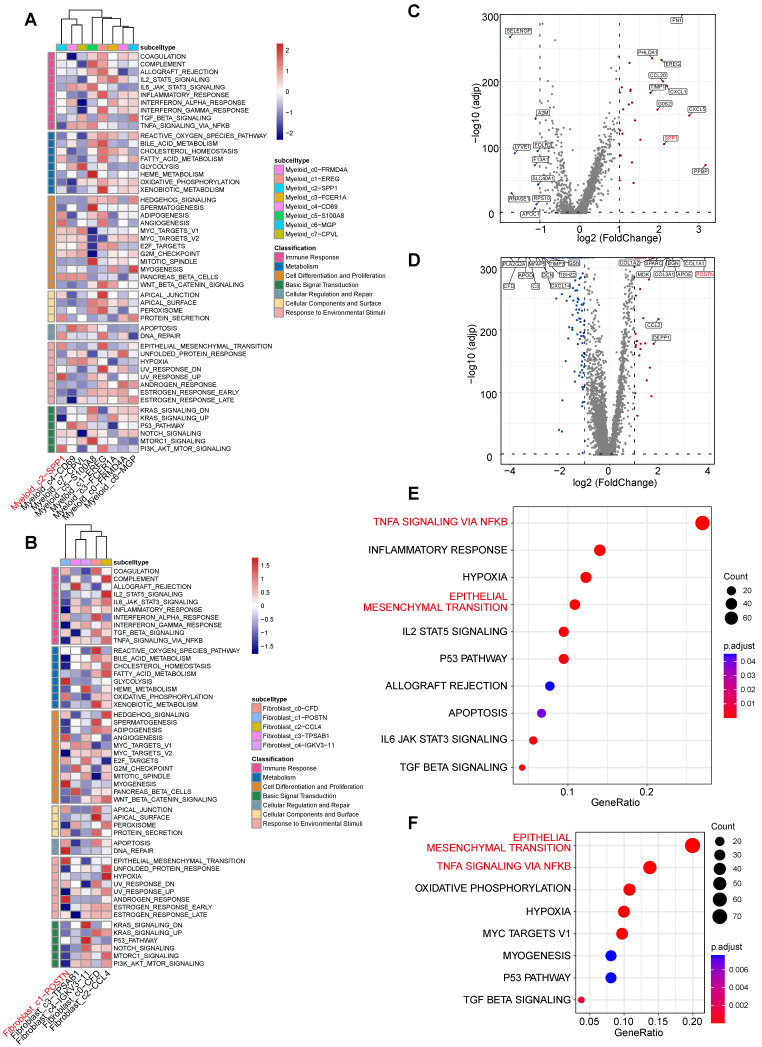
** Functional characterization of the myeloid_c2-*SPP1* and fibroblast_c1-*POSTN* subpopulations. A**, GSVA enrichment scores for myeloid subpopulations across MSigDB Hallmark gene sets. **B**, GSVA enrichment scores for fibroblast subpopulations across MSigDB Hallmark gene sets. **C-D**, Volcano plots depict significantly differentially expressed genes (DEGs) in myeloid_c2-*SPP1* (C) and fibroblast_c1-*POSTN* (D) subpopulations between TAA and control groups. **E**, Dot plot showing hallmark pathway enrichment based on upregulated DEGs in myeloid_c2-*SPP1* (TAA vs. control). Dot size indicates the number of DEGs per pathway, and color represents the adjusted *P* value (calculated using the BH method). **F**, Dot plot showing hallmark pathway enrichment based on upregulated DEGs in fibroblast_c1-*POSTN* (TAA vs. control), using the same visual metrics as in (E).

Myeloid_c2-*SPP1* was characterized by high expression of *SPP1* (osteopontin), a multifunctional secreted protein that interacts with integrin receptors to regulate cell adhesion, migration, proliferation, and ECM remodeling^37^. Gene Set Variation Analysis (GSVA) revealed significant enrichment of differentiation and proliferation-associated pathways, including MYC targets v1/v2, E2F targets, G2M checkpoint, adipogenesis, and angiogenesis, as well as tissue remodeling associated pathway epithelial mesenchymal transition (EMT) (Figure 6A). These findings suggest that Myeloid_c2-*SPP1* contributes to vascular wall dysfunction and ECM degradation through its involvement in cell proliferation, differentiation, and remodeling associated processes.

Similarly, Fibroblast_c1-*POSTN* exhibited high expression of *POSTN* (periostin), a key fibrotic mediator known to promote collagen crosslinking and ECM stiffening^38^. GSVA analysis indicated strong fibrotic proliferation and remodeling activity, marked by enrichment in MYC targets v1, mitotic spindle, EMT, and TGF-β signaling pathways (Figure 6B), collectively driving abnormal matrix deposition and remodeling.

Furthermore, we analyzed the DEGs and functional profiles of the two cell populations between the disease and control groups. The results showed that the subpopulation-specific genes *SPP1* and *POSTN* were significantly upregulated in the TAA group (Figure 6C-D). Functional enrichment of upregulated DEGs revealed that both populations in the TAA group accelerate the formation of a malignant microenvironment through similar signaling pathways. Notably, both Myeloid_c2-*SPP1* and Fibroblast_c1-*POSTN* exhibited significant activation of the TNF-α signaling via NF-κB, and EMT, as well as hypoxia (Figure 6E-F, Supplementary Figure 6). This convergence suggests a self-reinforcing interaction between Myeloid_c2-*SPP1* and Fibroblast_c1-*POSTN*. These mutual interactions may constitute a pathological feedback loop that amplifies pro-inflammatory and pro-fibrotic signals, thereby accelerating vascular wall damage and maladaptive remodeling in TAA.

### Convergent pathway activation driven by cell type-specific transcriptional regulation in Myeloid_c2-*SPP1* and Fibroblast_c1-*POSTN*

**Figure 7:**
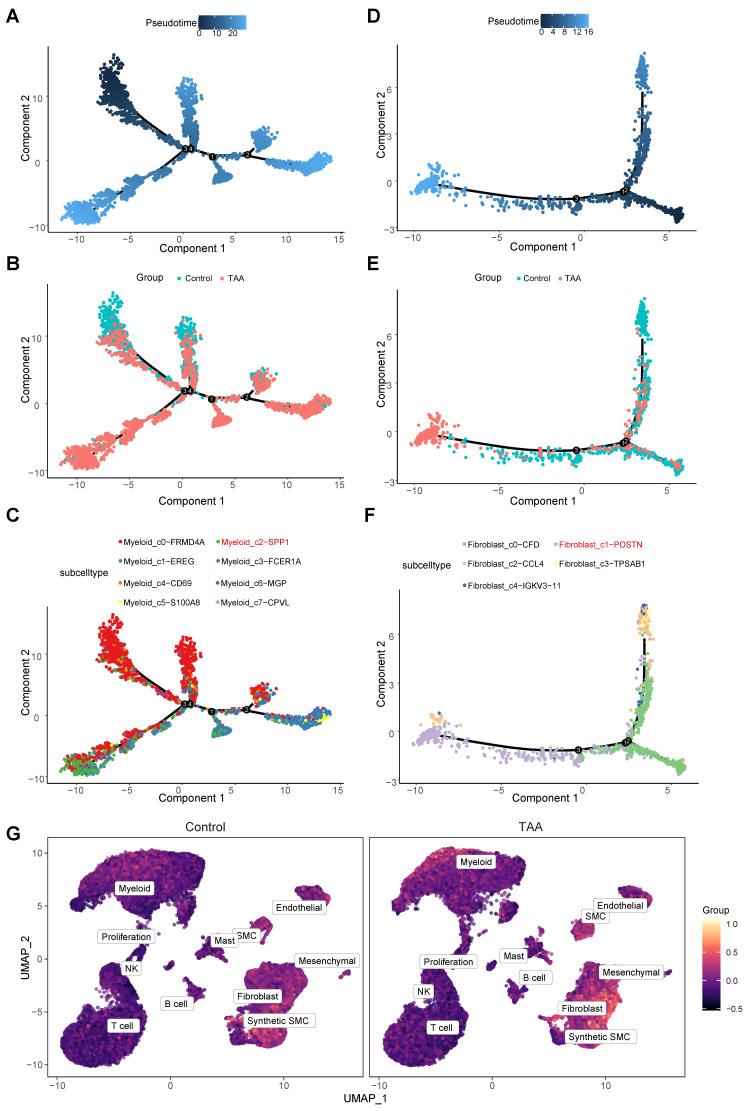
** Single-cell trajectory analysis of myeloid and fibroblast lineages using Monocle. A**, Pseudotime trajectory of myeloid cells during differentiation. **B**, Trajectories for myeloid cells of control and the TAA groups. **C**, Trajectories for myeloid cells of subtypes. **D**, Pseudotime trajectory of fibroblast cells during differentiation. **E**, Trajectories for fibroblast cells stratified of two groups. **F**, Trajectories for fibroblast cells of subtypes. **G**, Feature plots showing the hub gene score based on 18 core hub genes across control and TAA groups.

Pseudotime analysis revealed that both Myeloid_c2-*SPP1* and Fibroblast_c1-*POSTN* occupy late-stage positions along their respective developmental trajectories (Figure 7A-F), indicating that while they may not initiate disease onset, they likely represent terminally differentiated or activated cell states that intensify vascular wall damage during advanced stages of TAA.

To elucidate the upstream regulatory mechanisms driving their pathological roles, we conducted transcription factor (TF) prediction using DoRothEA. In the Myeloid_c2-*SPP1* subset, 14 specific regulators were significantly activated, with the top five being *PPARG*, *FOXP1*, *ERG*, *NR1H3*, and *FOXL2* (Supplementary Table 1). These TFs are primarily involved in the modulation of lipid metabolism (*PPARG*), regulation of immune cell function (*FOXP1*), promotion of angiogenesis (*ERG*), maintenance of cholesterol homeostasis (*NR1H3*), and ovarian development (*FOXL2*), suggesting their potential contribution to TAA pathogenesis by modulating lipid metabolism, regulating immune responses, promoting angiogenesis, and compromising vascular homeostasis, thereby facilitating chronic inflammation, aortic wall remodeling, and disease progression. Similarly, Fibroblast_c1-*POSTN* exhibited significant activation of 40 distinct TFs, with the top five being *TEAD1*, *PBX3*, *POU5F1*, *NANOG*, and *TCF4* (Supplementary Table 1). These regulators are mainly associated with ECM regulation (*TEAD1*), developmental gene regulation and ECM remodeling (*PBX3*), maintenance of stemness and cellular reprogramming (*POU5F1* and *NANOG*), and modulation of the Wnt/β-catenin signaling pathway (*TCF4*). Their activation implies a key role in promoting fibroblast activation, ECM remodeling, and pathological tissue repair, all of which are crucial processes contributing to TAA progression.

Despite distinct differences in transcriptional regulatory mechanisms, comparative transcriptomic analysis uncovered a convergence in downstream responses. Both subpopulations in the TAA group shared strong enrichment in TNF-α signaling via NF-κB pathway (33 overlapping genes, 27 of which were upregulated in both subpopulations, e.g., *ACKR3*, *CCL2*, *JUNB*, *NFKBIA*, *IL6*; Supplementary Table 2-3) and EMT pathway (21 genes, 16 of which were upregulated in both subpopulations, including *DCN*, *TPM4*, *TGFB1*, *TIMP1*, *IL6*; Supplementary Table 2-3). Intersection analysis further pinpointed five core hub genes: *GADD45B*, *GADD45A*, *PMEPA1*, *PTX3*, and *IL6* that were upregulated in the TAA group (Supplementary Table 2-3), suggesting a shared transcriptional mechanism underlying their inflammatory and fibrotic activities.

By integrating population-defining markers (*SPP1* and *POSTN*), disease-specific ligand-receptor pairs involved in cell-cell communication (*ITGAV*, *ITGB1*, *ITGA8*, *ITGA5*, *MDK*, *SDC4*, *SDC2*, *NCL*, *LRP1*, *ITGA6*, *ITGA4*), and the five shared crucial pathway associated genes (*GADD45B*, *GADD45A*, *PMEPA1*, *PTX3*, and *IL6*), we identified a set of 18 core hub genes. Using the AddModuleScore algorithm, we calculated a composite activity score for these genes and observed significantly elevated scores in myeloid cells and fibroblasts within the TAA group (Figure 7G), highlighting their potential functional relevance in disease progression. To explore upstream regulatory mechanisms, we conducted transcription factor (TF)-target prediction using the TRRUST database, which revealed that these hub genes are potentially regulated by eight key TFs: *POU5F1*, *POU2F1*, *HOXD3*, *PAX6*, *TWIST2*, *JUND*, *CEBPA*, and *SP1* (Supplementary Table 4, Supplementary Figure 7A). Within this network, *POU2F1* emerged as the most interconnected TF, and *SPP1* and *GADD45A* were the most frequently targeted genes (Supplementary Figure 7A).

To further support these predictions, we performed motif enrichment and regulon activity analyses using the pySCENIC workflow. Among the eight predicted transcription factors (TFs), six (*POU5F1*, *HOXD3*, *PAX6*, *TWIST2*, *JUND*, and *CEBPA*) were successfully enriched for specific binding motifs, with the number of enriched motifs ranked as *JUND* > *TWIST2* > *CEBPA* > *PAX6* > *HOXD3* > *POU5F1* (Supplementary Figure 7B). In contrast, *POU2F1* and *SP1* did not yield detectable motif enrichment, likely due to the absence of high-confidence binding motifs or low expression signal strength in our dataset. Notably, although *POU2F1* and *SP1* were identified as potential regulators by the TRRUST database, this discrepancy likely reflects the methodological differences between the two approaches. Regulon activity analysis further revealed distinct TF activation patterns between disease and control groups (Supplementary Figure 7C). Specifically, *CEBPA* was highly activated in Myeloid_c2-*SPP1* cells of the TAA group, whereas *JUND* showed enhanced activity in Fibroblast_c1-*POSTN* cells. Moreover, *PAX6* and *TWIST2* exhibited stronger activity in fibroblasts than in myeloid cells, while *CEBPA* displayed the opposite trend. *HOXD3* and *POU5F1* were broadly activated across both fibroblast and myeloid subclusters. These results provide convergent computational evidence supporting the involvement of these TFs in cell type-specific transcriptional regulation associated with inflammatory and ECM remodeling in TAA.

Collectively, these findings suggest that although Myeloid_c2-*SPP1* and Fibroblast_c1-*POSTN* are regulated by distinct, cell type-specific transcriptional programs that nonetheless converge on shared inflammatory and fibrotic pathways, thereby reinforcing pathological intercellular communication in TAA.

### Experimental and spatial transcriptomic validation of *SPP1*^+^ myeloid and *POSTN*^+^ fibroblast colocalization

**Figure 8:**
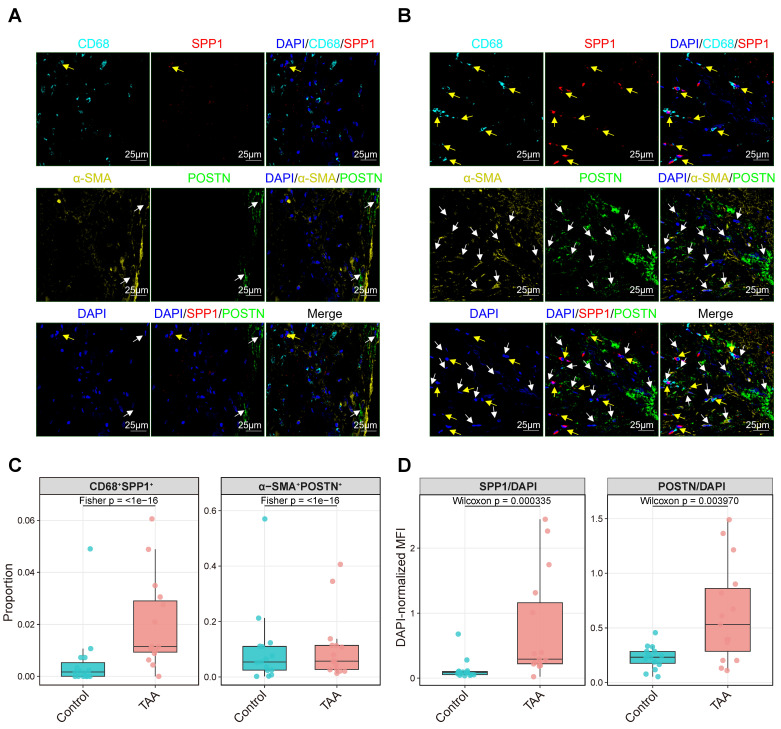
** Multiplex immunofluorescence validation of SPP1^+^ myeloid and POSTN^+^ fibroblast colocalization, with regional quantification of double-positive cell proportions and DAPI-normalized fluorescence intensity.** Representative multiplex immunofluorescence images of control **(A)** and TAA tissue sections **(B)** stained for DAPI (nuclei, blue), CD68 (myeloid cells, cyan), SPP1 (red), α-SMA (activated fibroblasts, yellow), and POSTN (green). Yellow arrows indicate SPP1^+^ myeloid cells, and white arrows indicate POSTN^+^ fibroblasts. Scale bars, 25 μm. **(C)** Proportions of CD68^+^SPP1^+^ and α-SMA^+^POSTN^+^ double-positive cells per region (each dot = one region; boxplots show median and IQR); group differences were assessed by Fisher's exact test on pooled counts, with p values shown. **(D)** DAPI-normalized MFI (SPP1/DAPI and POSTN/DAPI) per region (each dot = one region; boxplots show median and IQR); group differences were assessed by Mann-Whitney U test (Wilcoxon rank-sum), with p values shown. Colors indicate groups.

**Figure 9:**
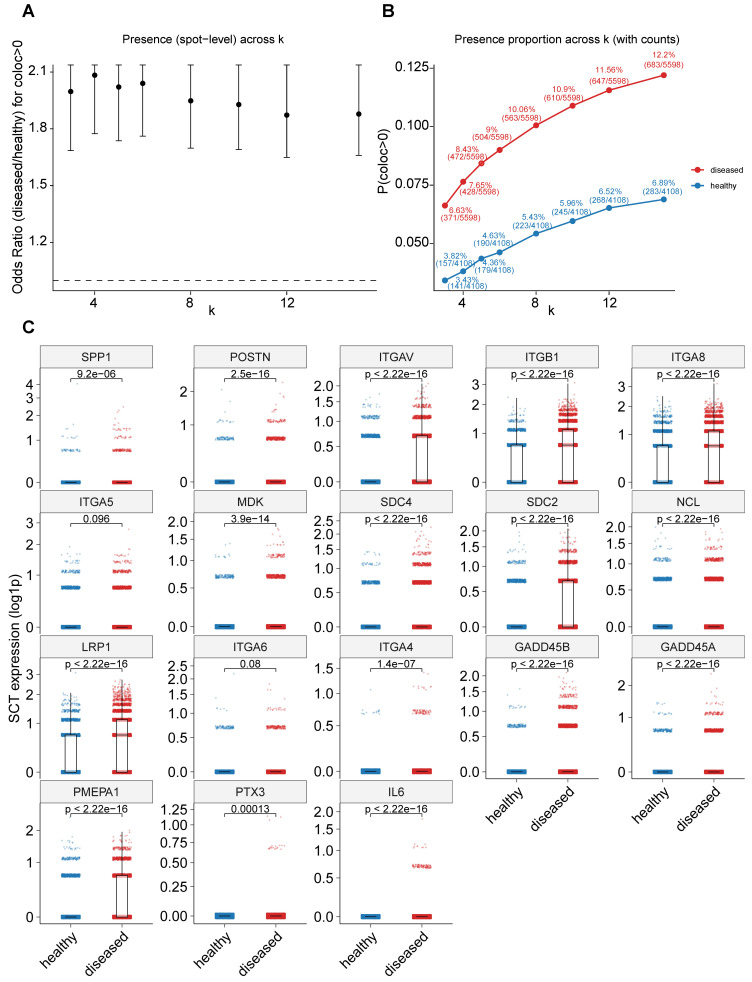
** Spatial transcriptomic validation of *SPP1*^+^ myeloid and *POSTN*^+^ fibroblast colocalization. A,** Odds ratios (ORs) for detecting spatial colocalization (*coloc* > 0) between *SPP1*^+^ myeloid and *POSTN*^+^ fibroblast signatures across varying k-nearest-neighbor values (k = 3, 4, 5, 6, 8, 10, 12, and 15). Points represent OR estimates comparing disease versus healthy samples, and error bars indicate 95% confidence intervals derived from one-sided Fisher's exact tests (disease > healthy). The dashed line denotes OR = 1. **B,** Proportion of *coloc*-positive spots in disease and healthy samples across different k values. Points indicate *coloc* positivity rates, with labels showing percentages and raw counts (n_pos/n_total); lines illustrate trends across spatial scales. **C,** Expression levels of 18 core hub genes comparing healthy and disease samples. Each dot represents a spot, and gene expression is shown as log-transformed values. *P* values were calculated using one-sided Wilcoxon rank-sum tests.

To independently validate the computationally inferred interaction between *SPP1*^+^ myeloid cells and *POSTN*^+^ fibroblasts, we performed multiplex immunofluorescence on an independent set of TAA and control tissues (1 case each; Figure 8) and further assessed spatial colocalization using an independent spatial transcriptomics dataset (2 TAA and 2 controls; Figure 9). These validation samples were independent of the scRNA-seq cohort, and their clinical characteristics are summarized in Supplementary Table 5.

For the multiplex immunofluorescence (mIF) validation, markers for myeloid cells (CD68), SPP1, activated fibroblasts (α-SMA), and POSTN were simultaneously visualized (Figure 8A-B). For quantitative analysis, 15 regions of interest (ROIs; 150 μm × 150 μm) were randomly selected per group, and the proportions of CD68^+^SPP1^+^ and α-SMA^+^POSTN^+^ double-positive cells as well as DAPI-normalized MFI (SPP1/DAPI and POSTN/DAPI) were calculated in each ROI. Representative TAA sections showed an apparent increase in both *SPP1*^+^ myeloid cells and *POSTN*^+^ fibroblasts compared with control, accompanied by increased spatial proximity between these populations. Merged images revealed evident colocalization of SPP1 and POSTN signals within fibrotic regions, whereas control tissue showed sparse expression and minimal overlap. Consistent with these observations, regional quantification demonstrated a higher proportion of CD68^+^SPP1^+^ and α-SMA^+^POSTN^+^ double-positive cells in TAA compared with control (Figure 8C), together with significant differences in DAPI-normalized SPP1 and POSTN fluorescence across regions (Figure 8D).

Consistent with these histological observations, analysis of independent public spatial transcriptomics datasets derived from Marfan syndrome-associated aortic root aneurysm, a fibrotic aortic disease sharing key pathological features with thoracic aortic aneurysm, provided additional support for the enhanced interaction between *SPP1*^+^ myeloid and *POSTN*^+^ fibroblasts in disease tissues. This dataset is deposited in the Genome Sequence Archive (GSA; accession no. HRA004063)^17^. Spatial colocalization analysis revealed that disease samples exhibited significantly higher odds ratios for *coloc*-positive spots and consistently elevated *coloc*-positive proportions at the spot level across multiple k-nearest-neighbor values (Figure 9A-B). Notably, nearly all of the 18 predefined core hub genes, identified from single-cell analyses based on population markers, ligand-receptor interactions, and shared pathway activation, were upregulated in disease samples versus controls, with 16 of 18 showing significant increases and the remaining two exhibiting similar upward trends (Figure 9C). Together, these experimental and spatial transcriptomic data provide convergent evidence supporting increased abundance and enhanced spatial colocalization of *SPP1*^+^ myeloid cells and *POSTN*^+^ fibroblasts in diseased aortic tissues.

## Discussion

This study systematically deciphered the critical roles of myeloid cells and fibroblasts in the progression of TAA through single-cell transcriptomic analyses. Our findings not only reveal the functional characteristics of key cellular subpopulations but also elucidate how they form a vicious cycle through specific signaling pathways and transcriptional regulatory networks, collectively contributing to vascular wall injury and remodeling in TAA.

First, we identified two novel subpopulations, Myeloid_c2-*SPP1* and Fibroblast_c1-*POSTN*, that play pivotal roles in TAA progression. In TAA, myeloid cells (e.g., macrophages, monocytes) contribute to vascular wall inflammation and ECM degradation by secreting pro-inflammatory cytokines and proteolytic enzymes, thereby activating inflammatory signaling pathways such as NF-κB^1^. In our study, Myeloid_c2-*SPP1* subset displayed strong *SPP1* expression and was significantly enriched in TAA samples. Elevated plasma *SPP1* levels have been linked to increased TAA risk^39^, ^40^, and *SPP1^+^* macrophages have been shown to promote fibrosis and endothelial-mesenchymal transition (EndoMT) in degenerative ascending aortic aneurysms^41^, ^42^. Mechanistically, *SPP1* may activate the TGF-β signaling pathway via integrin receptors (e.g., ITGAV, ITGA8, ITGA5, ITGB1), inducing fibroblast-to-myofibroblast differentiation and amplifying inflammation via NF-κB and NLRP3 inflammasome activation^43^-^45^.

On the other hand, we observed a notable enrichment of the Fibroblast_c1-*POSTN* subpopulation in TAA tissues, despite an overall reduction in fibroblast abundance. *POSTN*, a matricellular protein, regulates ECM remodeling and activates TGF-β, NF-κB, and PI3K/Akt signaling to drive fibrosis and chronic inflammation^46^. Its expression is normally low but rapidly induced following acute vascular injury, and is upregulated in aortic aneurysm tissues^47^-^49^. Notably, Fibroblast_c1-*POSTN* also serves as a major source of MK signaling. MK, a heparin-binding growth factor involved in cell proliferation, survival, migration, and inflammation^50^, ^51^. MK interacts with several receptors such as SDC4/SDC2, LRP1, ITGA4/ITGB1, and NCL to promote macrophage recruitment and fibroblast activation, thereby contributing to pathological ECM remodeling and vascular wall weakening^52^-^56^.

Second, our study identifies a synergistic interaction between *SPP1*^+^ myeloid cells and *POSTN*^+^ fibroblasts that contributes to the progression of TAA. While previous single-cell studies have advanced our understanding of TAA pathogenesis, they mainly focused on the heterogeneity of individual cell types. For instance, Li et al. (2020) mapped the cellular composition of ascending thoracic aortic aneurysm and highlighted regulators like ERG but did not explore immune-stromal crosstalk^7^. Liu et al. (2022) identified *Il1rn*^+^/*Trem*1^+^ pro-inflammatory macrophages as key drivers of inflammation and aneurysm rupture but did not examine their effects on fibroblast activation or ECM remodeling^8^. In contrast, our analysis reveals a specific interaction between *SPP1*^+^ myeloid cells and *POSTN*^+^ fibroblasts, mediated by SPP1 and MK signaling. Both SPP1 and MK are secreted matricellular proteins that exert overlapping roles in inflammation, angiogenesis, and fibrosis through shared receptor complexes, including integrins (ITGA5/ITGB1), and downstream PI3K/AKT, JAK/STAT, and NF-κB signaling^22^, ^57^. In the aneurysmal microenvironment, myeloid-derived SPP1 may activate fibroblasts via integrin-mediated signaling, potentially enhancing ECM production, while fibroblast-derived MK may further recruit macrophages and potentially amplify inflammatory cascades through NF-κB and STAT3 activation. This feedback loop could contribute to TNF-α signaling mediated inflammation and promote ECM remodeling, processes that are likely central to TAA pathogenesis. Compared to the general macrophage-fibroblast interactions described by Wang et al. (2022)^19^, our findings specify unique cellular subsets and signaling pathways, offering new mechanistic insights and suggesting the SPP1 and MK signaling as potential therapeutic targets. Similar SPP1/MK signaling has also been observed in lung adenocarcinoma^58^, supporting its broader relevance in pathological tissue remodeling. Clinically, the concurrent upregulation of SPP1 and MK signaling in TAA tissues raises the intriguing possibility that their circulating protein levels may serve as non-invasive biomarkers for disease diagnosis or monitoring. Both SPP1 (osteopontin) and MK are secreted proteins detectable in plasma and have been reported to correlate with disease severity in various cardiovascular and inflammatory conditions^59^, ^60^. Quantifying serum SPP1 and MDK concentrations could therefore provide complementary insights into the inflammatory and remodeling status of the aortic wall. Future prospective studies combining plasma proteomics, imaging, and histopathological validation are warranted to assess their predictive value for TAA onset, progression, and postoperative outcomes, ultimately facilitating early detection and individualized therapeutic stratification.

Third, our study shows that despite their distinct transcriptional profiles, Myeloid_c2-*SPP1* and Fibroblast_c1-*POSTN* synergistically promote inflammation and ECM remodeling in TAA through coordinated activation of the TNF-α signaling via NF-κB and EMT pathways, supported by shared hub genes. These findings provide important molecular insights into the cellular crosstalk driving TAA pathogenesis.

Shared inflammatory signaling mechanisms were observed between the two cell populations, with 27 out of 33 key genes (e.g., *IL6*, *CCL2*, *NFKBIA*) co-upregulated in the TAA group within the TNF-α signaling via NF-κB pathway, underscoring the central role of inflammation in TAA progression^7^, ^61^. Moreover, activation of the EMT pathway, evidenced by the co-upregulation of 16 out of 21 genes (e.g., *TGFB1*, *TIMP1*, *DCN*) in the TAA group suggests that both cell types may enhance migratory capacity and ECM synthesis through EMT-like processes. However, dysregulated EMT can further aggravate fibrosis or matrix degradation^62^. Notably, five cross-pathway hub genes (*GADD45B*/*A*, *PMEPA1*, *PTX3*, and *IL6*) were significantly upregulated in both cell types, potentially serving as key signaling bridges coordinating inflammation and ECM remodeling. For instance, *IL6*, a pro-inflammatory cytokine and NF-κB target, forms a positive feedback loop with STAT3 to amplify inflammatory responses, while also inducing *POSTN* expression in fibroblasts, thereby promoting fibrosis^63^. The GADD45 family is closely associated with inflammation, fibrosis, and oxidative stress in various tissues; *GADD45A* has been reported to exert a cytoprotective effect by modulating these processes^64^, whereas *GADD45B* , induced by stressors such as *IL-6*, *TNF-α*, *TGF-β*, and LPS, is involved in immune response and key regulators of fibrotic processes^65^, ^66^, suggesting a tissue microenvironment enriched in inflammatory and fibrotic features in TAA. *PMEPA1* has been implicated in EMT promotion and may modulate NF-κB signaling via its antisense lncRNA NKILA^67^-^69^. Additionally, *PTX3* activates NF-κB to upregulate *MMP3* and *POSTN*, thereby reinforcing EMT-like phenotypes and contributing to ECM remodeling^70^, ^71^.

Furthermore, to further elucidate the upstream regulatory mechanisms governing the functional states of Myeloid_c2-*SPP1* and Fibroblast_c1-*POSTN*, we integrated three categories of hub genes: highly expressed marker genes (*SPP1* and *POSTN*), ligand-receptor interaction molecules (including *ITGAV*, *ITGB1*, *ITGA8*, *ITGA5*, *MDK*, *SDC4*, *SDC2*, *NCL*, *LRP1*, *ITGA6*, and *ITGA4*), and core genes co-upregulated in inflammatory and EMT pathways (e.g., *GADD45B*, *GADD45A*, *PMEPA1*, *PTX3*, and *IL6*), totaling 18 hub genes. Using the TRRUST database, we identified eight TFs were predicted as upstream regulators: *POU5F1*, *POU2F1*, *HOXD3*, *PAX6*, *TWIST2*, *JUND*, *CEBPA*, and *SP1* (Supplementary Table 4). Subsequent pySCENIC motif enrichment and regulon activity analyses largely supported these predictions. Among them, six TFs (*JUND*, *TWIST2*, *CEBPA*, *PAX6*, *HOXD3*, *POU5F1*) exhibited significant motif enrichment and cell type-specific activity, with* CEBPA* preferentially active in Myeloid_c2-*SPP1* and *JUND* in Fibroblast_c1-*POSTN*. Although direct evidence of their involvement in TAA remains limited, prior studies support their critical roles in aortic wall remodeling, inflammation, and cell migration. For example, *JUND*, a component of the AP-1 transcriptional complex, regulates degenerative gene mechanisms in smooth muscle cells, acting as a critical driver of aortic dissection and rupture^72^.* TWIST2* plays a key role in EMT, with its suppression impairing multiple steps of peritoneal metastasis^73^.* CEBPA* directly binds and upregulates *SPP1*, modulating cell migration and angiogenesis^74^. The 5a splice variant of *PAX6* upregulates the integrin complex *ITGA5+ITGB1* in lens fiber cells, contributing to cataract formation^75^.* HOXD3* activates integrin β3^76^ and enhance the expression and function of integrin α5β1^77^.*POU5F1* (*OCT4*) induces dedifferentiation of human aortic smooth muscle cells via *KLF5* upregulation, contributing to the thoracic aortic dissection pathogenesis^78^. Notably, our TF-target interaction analysis identified cell type-specific TF modules in Myeloid_c2-*SPP1* and Fibroblast_c1-*POSTN*. These distinct modules may nonetheless converge to promote inflammation and ECM remodeling. Collectively, the identified signaling pathways, hub genes and common TFs may inform future therapeutic strategies for TAA.

Importantly, the key computational findings of this study were supported by independent histological and spatial transcriptomic validations. Multiplex immunofluorescence analyses of aortic tissues confirmed increased abundance and spatial proximity of *SPP1*^+^ myeloid cells and *POSTN*^+^ fibroblasts in the TAA sample, providing direct tissue-level evidence for immune stromal colocalization. Consistently, analysis of an independent public spatial transcriptomics dataset derived from Marfan syndrome-associated aortic root aneurysm further demonstrated enriched spatial colocalization between *SPP1*^+^ myeloid and *POSTN*^+^ fibroblast signatures, together with elevated expression of 18 core hub genes. Although derived from a related but distinct aortic disease context, these spatial data capture shared features of aortic inflammation and ECM remodeling, reinforcing the biological relevance of the immune and stromal interaction axis mediated by SPP1 and MK. Together, these orthogonal validations strengthen the conclusion that spatially organized crosstalk between myeloid cells and fibroblasts represents a conserved pathogenic mechanism in aortic wall remodeling.

**Figure 10:**
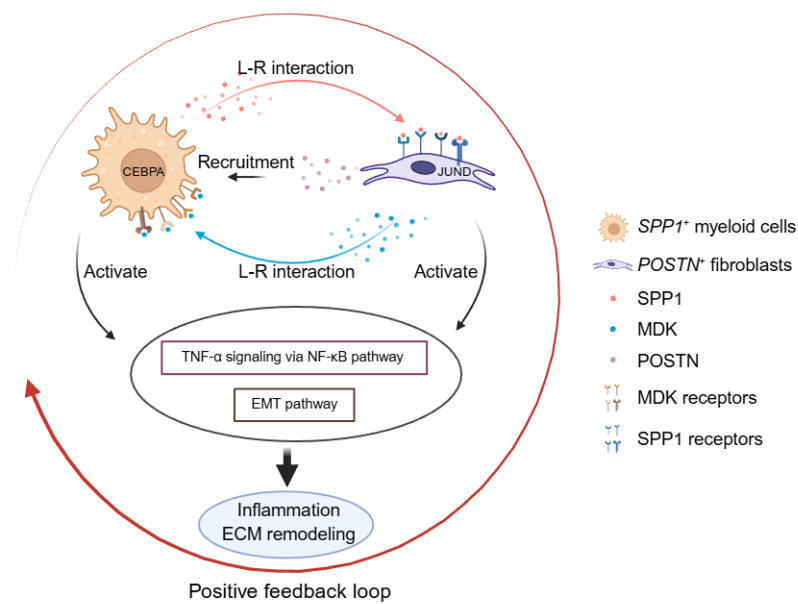
** Schematic model of the disease-associated SPP1-POSTN-MDK circuit in thoracic aortic aneurysm (TAA).** In TAA, SPP1^+^ myeloid cells (Myeloid_c2-SPP1) secrete SPP1, which activates POSTN^+^ fibroblasts (Fibroblast_c1-POSTN) through SPP1-integrin interactions (e.g., ITGAV/ITGA8/ITGA5-ITGB1). Activated fibroblasts upregulate POSTN and MDK, and fibroblast-derived MDK signals back to myeloid cells via MDK receptors (e.g., SDC4/SDC2/NCL/LRP1/ITGA4/ITGA6-ITGB1), reinforcing myeloid recruitment/activation and forming a positive feedback loop. This circuit converges on TNF-α signaling via NF-κB and epithelial-mesenchymal transition (EMT), whose downstream effects are executed through cell type-specific transcriptional programs, thereby amplifying inflammation and ECM remodeling and ultimately exacerbating vascular wall damage.

In summary, this study establishes an integrated single-cell framework for thoracic aortic aneurysm pathogenesis and identifies a disease associated inflammatory and fibrotic circuit between *SPP1*^+^ myeloid cells and *POSTN*^+^ fibroblasts. Through signaling mediated by SPP1 and MK, these cell populations coordinate immune activation and ECM remodeling, thereby promoting vascular wall degeneration. While pathways such as TNF-α signaling via NF-κB and EMT are broadly activated across cell types in TAA, their functional execution is governed by distinct, cell type-specific transcriptional programs (Figure 10). This organization highlights how shared inflammatory cues are translated into specialized cellular responses that collectively drive aortic wall remodeling. Together with spatial validation, our findings underscore immune-stromal crosstalk as a central mechanism in TAA progression and point to a targetable signaling network with therapeutic potential.

## Limitations

Despite these insights, several limitations should be acknowledged. First, we integrated publicly available scRNA-seq datasets, and key observations were further supported by multiplex immunofluorescence and independent spatial transcriptomics. However, the multiplex immunofluorescence validation cohort was small (n = 3), which may limit statistical power and generalizability across clinical contexts. In addition, the spatial transcriptomics data used for validation were derived from Marfan syndrome-associated aortic root aneurysm rather than sporadic TAA, reflecting the limited availability of public spatial transcriptomics datasets for sporadic TAA; nevertheless, shared features of aortic inflammation and ECM remodeling support the relevance of this validation. Finally, the clinical applicability of SPP1 and MK signaling as circulating biomarkers or therapeutic targets requires confirmation in prospective and longitudinal studies. Despite these limitations, our study provides a systematic framework for understanding immune-stromal crosstalk in TAA and generates testable hypotheses for future investigation.

## Supplementary Material

Supplementary figures and tables.

## Data Availability

The raw scRNA-seq datasets analyzed in this study are publicly available from the NCBI Sequence Read Archive (SRA) under accession numbers PRJNA919181 (corresponding to GEO accession GSE222318) and PRJNA649846 (corresponding to GEO accession GSE155468). In addition, the spatial transcriptomics dataset analyzed in this study is publicly available from the Genome Sequence Archive (GSA) under accession number HRA004063.

## References

[1] Gao J, Cao H, Hu G, Wu Y, Xu Y, Cui H (2023). The mechanism and therapy of aortic aneurysms. Signal Transduct Target Ther.

[2] Da X, Li Z, Huang X, He Z, Yu Y, Tian T (2023). AGGF1 therapy inhibits thoracic aortic aneurysms by enhancing integrin alpha7-mediated inhibition of TGF-beta1 maturation and ERK1/2 signaling. Nat Commun.

[3] Wu J, Zafar MA, Liu Y, Chen JF, Li Y, Ziganshin BA (2023). Fate of the unoperated ascending thoracic aortic aneurysm: three-decade experience from the Aortic Institute at Yale University. Eur Heart J.

[4] McClure RS, Brogly SB, Lajkosz K, Payne D, Hall SF, Johnson AP (2018). Epidemiology and management of thoracic aortic dissections and thoracic aortic aneurysms in Ontario, Canada: A population-based study. J Thorac Cardiovasc Surg.

[5] Bossone E, Eagle KA (2021). Epidemiology and management of aortic disease: aortic aneurysms and acute aortic syndromes. Nat Rev Cardiol.

[6] Martin-Blazquez A, Heredero A, Aldamiz-Echevarria G, Martin-Lorenzo M, Alvarez-Llamas G (2021). Non-syndromic thoracic aortic aneurysm: cellular and molecular insights. J Pathol.

[7] Li Y, Ren P, Dawson A, Vasquez HG, Ageedi W, Zhang C (2020). Single-Cell Transcriptome Analysis Reveals Dynamic Cell Populations and Differential Gene Expression Patterns in Control and Aneurysmal Human Aortic Tissue. Circulation.

[8] Liu X, Chen W, Zhu G, Yang H, Li W, Luo M (2022). Single-cell RNA sequencing identifies an Il1rn(+)/Trem1(+) macrophage subpopulation as a cellular target for mitigating the progression of thoracic aortic aneurysm and dissection. Cell Discov.

[9] Ganizada BH, Veltrop RJA, Akbulut AC, Koenen RR, Accord R, Lorusso R (2024). Unveiling cellular and molecular aspects of ascending thoracic aortic aneurysms and dissections. Basic Res Cardiol.

[10] Wang X, Zhang H, Cao L, He Y, Ma A, Guo W (2020). The Role of Macrophages in Aortic Dissection. Front Physiol.

[11] Chen R, Zhang H, Tang B, Luo Y, Yang Y, Zhong X (2024). Macrophages in cardiovascular diseases: molecular mechanisms and therapeutic targets. Signal Transduct Target Ther.

[12] Cheng Z, Zhou YZ, Wu Y, Wu QY, Liao XB, Fu XM (2018). Diverse roles of macrophage polarization in aortic aneurysm: destruction and repair. J Transl Med.

[13] Mackay CDA, Jadli AS, Fedak PWM, Patel VB (2022). Adventitial Fibroblasts in Aortic Aneurysm: Unraveling Pathogenic Contributions to Vascular Disease. Diagnostics (Basel).

[14] Gibb AA, Lazaropoulos MP, Elrod JW (2020). Myofibroblasts and Fibrosis: Mitochondrial and Metabolic Control of Cellular Differentiation. Circ Res.

[15] Jones JA, Beck C, Barbour JR, Zavadzkas JA, Mukherjee R, Spinale FG (2009). Alterations in aortic cellular constituents during thoracic aortic aneurysm development: myofibroblast-mediated vascular remodeling. Am J Pathol.

[16] Liu XW, Wang P, Zhang L, Zhu Y, Zhai JY, Wang CN (2024). Single-cell RNA sequencing and ATAC sequencing identify novel biomarkers for bicuspid aortic valve-associated thoracic aortic aneurysm. Front Cardiovasc Med.

[17] Liu X, Zeng Q, Yang H, Li W, Chen Q, Yin K (2024). Single-Nucleus Multiomic Analyses Identifies Gene Regulatory Dynamics of Phenotypic Modulation in Human Aneurysmal Aortic Root. Adv Sci (Weinh).

[18] Cao G, Xuan X, Li Y, Hu J, Zhang R, Jin H (2023). Single-cell RNA sequencing reveals the vascular smooth muscle cell phenotypic landscape in aortic aneurysm. Cell Commun Signal.

[19] Wang Q, Guo X, Huo B, Feng X, Fang ZM, Jiang DS (2022). Integrating Bulk Transcriptome and Single-Cell RNA Sequencing Data Reveals the Landscape of the Immune Microenvironment in Thoracic Aortic Aneurysms. Front Cardiovasc Med.

[20] Chou EL, Chaffin M, Simonson B, Pirruccello JP, Akkad AD, Nekoui M (2022). Aortic Cellular Diversity and Quantitative Genome-Wide Association Study Trait Prioritization Through Single-Nuclear RNA Sequencing of the Aneurysmal Human Aorta. Arterioscler Thromb Vasc Biol.

[21] Tsai SL, Baselga-Garriga C, Melton DA (2020). Midkine is a dual regulator of wound epidermis development and inflammation during the initiation of limb regeneration. Elife.

[22] Neumaier EE, Rothhammer V, Linnerbauer M (2023). The role of midkine in health and disease. Front Immunol.

[23] Weckbach LT, Preissner KT, Deindl E (2018). The Role of Midkine in Arteriogenesis, Involving Mechanosensing, Endothelial Cell Proliferation, and Vasodilation. Int J Mol Sci.

[24] Zhang ZZ, Wang G, Yin SH, Yu XH (2021). Midkine: A multifaceted driver of atherosclerosis. Clin Chim Acta.

[25] Lautz T, Lasch M, Borgolte J, Troidl K, Pagel JI, Caballero-Martinez A (2018). Midkine Controls Arteriogenesis by Regulating the Bioavailability of Vascular Endothelial Growth Factor A and the Expression of Nitric Oxide Synthase 1 and 3. EBioMedicine.

[26] Wolock SL, Lopez R, Klein AM (2019). Scrublet: Computational Identification of Cell Doublets in Single-Cell Transcriptomic Data. Cell Syst.

[27] Butler A, Hoffman P, Smibert P, Papalexi E, Satija R (2018). Integrating single-cell transcriptomic data across different conditions, technologies, and species. Nat Biotechnol.

[28] Korsunsky I, Millard N, Fan J, Slowikowski K, Zhang F, Wei K (2019). Fast, sensitive and accurate integration of single-cell data with Harmony. Nat Methods.

[29] Yu G, Wang LG, Han Y, He QY (2012). clusterProfiler: an R package for comparing biological themes among gene clusters. OMICS.

[30] Liberzon A, Birger C, Thorvaldsdottir H, Ghandi M, Mesirov JP, Tamayo P (2015). The Molecular Signatures Database (MSigDB) hallmark gene set collection. Cell Syst.

[31] Hanzelmann S, Castelo R, Guinney J (2013). GSVA: gene set variation analysis for microarray and RNA-seq data. BMC Bioinformatics.

[32] Jin S, Guerrero-Juarez CF, Zhang L, Chang I, Ramos R, Kuan CH (2021). Inference and analysis of cell-cell communication using CellChat. Nat Commun.

[33] Trapnell C, Cacchiarelli D, Grimsby J, Pokharel P, Li S, Morse M (2014). The dynamics and regulators of cell fate decisions are revealed by pseudotemporal ordering of single cells. Nat Biotechnol.

[34] Garcia-Alonso L, Holland CH, Ibrahim MM, Turei D, Saez-Rodriguez J (2019). Benchmark and integration of resources for the estimation of human transcription factor activities. Genome Res.

[35] Han H, Cho JW, Lee S, Yun A, Kim H, Bae D (2018). TRRUST v2: an expanded reference database of human and mouse transcriptional regulatory interactions. Nucleic Acids Res.

[36] Van de Sande B, Flerin C, Davie K, De Waegeneer M, Hulselmans G, Aibar S (2020). A scalable SCENIC workflow for single-cell gene regulatory network analysis. Nat Protoc.

[37] Yim A, Smith C, Brown AM (2022). Osteopontin/secreted phosphoprotein-1 harnesses glial-, immune-, and neuronal cell ligand-receptor interactions to sense and regulate acute and chronic neuroinflammation. Immunol Rev.

[38] Dorafshan S, Razmi M, Safaei S, Gentilin E, Madjd Z, Ghods R (2022). Periostin: biology and function in cancer. Cancer Cell Int.

[39] Zhou M, Zhu Y, Zhou Z, Qi F, Zheng S, Gao S (2022). Fibroblast-Secreted Phosphoprotein 1 Mediates Extracellular Matrix Deposition and Inhibits Smooth Muscle Cell Contractility in Marfan Syndrome Aortic Aneurysm. J Cardiovasc Transl Res.

[40] Chen R, Lehmann HI, Xiao J (2022). Novel Effector Molecules Regulating Smooth Muscle Cell Contractility in Marfan Syndrome: Phosphoprotein 1 Secreted by Fibroblasts. J Cardiovasc Transl Res.

[41] Freiholtz D, Bergman O, Pradhananga S, Lang K, Poujade FA, Granath C (2023). SPP1/osteopontin: a driver of fibrosis and inflammation in degenerative ascending aortic aneurysm?. J Mol Med (Berl).

[42] Hoeft K, Schaefer GJL, Kim H, Schumacher D, Bleckwehl T, Long Q (2023). Platelet-instructed SPP1(+) macrophages drive myofibroblast activation in fibrosis in a CXCL4-dependent manner. Cell Rep.

[43] Li R, Chen B, Kubota A, Hanna A, Humeres C, Hernandez SC (2023). Protective effects of macrophage-specific integrin alpha5 in myocardial infarction are associated with accentuated angiogenesis. Nat Commun.

[44] Gu H, Li Q, Liu Z, Li Y, Liu K, Kong X (2025). SPP1-ITGalpha5/beta1 Accelerates Calcification of Nucleus Pulposus Cells by Inhibiting Mitophagy via Ubiquitin-Dependent PINK1/PARKIN Pathway Blockade. Adv Sci (Weinh).

[45] Leask A, Hutchenreuther J (2014). Activation of latent TGFbeta by alphavbeta 1 integrin: of potential importance in myofibroblast activation in fibrosis. J Cell Commun Signal.

[46] Wang Z, An J, Zhu D, Chen H, Lin A, Kang J (2022). Periostin: an emerging activator of multiple signaling pathways. J Cell Commun Signal.

[47] Nie X, Shen C, Tan J, Wu Z, Wang W, Chen Y (2020). Periostin: A Potential Therapeutic Target For Pulmonary Hypertension?. Circ Res.

[48] Yagi H, Nishigori M, Murakami Y, Osaki T, Muto S, Iba Y (2020). Discovery of novel biomarkers for atherosclerotic aortic aneurysm through proteomics-based assessment of disease progression. Sci Rep.

[49] Bruckner A, Brandtner A, Rieck S, Matthey M, Geisen C, Fels B (2024). Site-specific genetic and functional signatures of aortic endothelial cells at aneurysm predilection sites in healthy and AngII ApoE(-/-) mice. Angiogenesis.

[50] Huang H, Li J, Lu Y, Min L, Li D, Dai L (2015). Role of midkine-progranulin interaction during angiogenesis of hepatocellular carcinoma. Int J Clin Exp Pathol.

[51] Zhang S, Zhang L, Wang L, Wang H, Wu J, Cai H (2023). Machine learning identified MDK score has prognostic value for idiopathic pulmonary fibrosis based on integrated bulk and single cell expression data. Front Genet.

[52] Actis Dato V, Chiabrando GA (2018). The Role of Low-Density Lipoprotein Receptor-Related Protein 1 in Lipid Metabolism, Glucose Homeostasis and Inflammation. Int J Mol Sci.

[53] Zhang Y, Zuo C, Liu L, Hu Y, Yang B, Qiu S (2021). Single-cell RNA-sequencing atlas reveals an MDK-dependent immunosuppressive environment in ErbB pathway-mutated gallbladder cancer. J Hepatol.

[54] Zhu Z, Ling X, Zhou H, Xie J (2023). Syndecan-4 is the key proteoglycan involved in mediating sepsis-associated lung injury. Heliyon.

[55] Li X, Zhao S, Bian X, Zhang L, Lu L, Pei S (2022). Signatures of EMT, immunosuppression, and inflammation in primary and recurrent human cutaneous squamous cell carcinoma at single-cell resolution. Theranostics.

[56] Yu X, Xie L, Ge J, Li H, Zhong S, Liu X (2023). Integrating single-cell RNA-seq and spatial transcriptomics reveals MDK-NCL dependent immunosuppressive environment in endometrial carcinoma. Front Immunol.

[57] Jung S, Ha J, Park JH, Yoo KH (2025). Decoding SPP1 regulation: Genetic and nongenetic insights into its role in disease progression. Mol Cells.

[58] Wang X, Chen J, Li C, Liu Y, Chen S, Lv F (2024). Integrated bulk and single-cell RNA sequencing identifies an aneuploidy-based gene signature to predict sensitivity of lung adenocarcinoma to traditional chemotherapy drugs and patients' prognosis. PeerJ.

[59] Zhao Y, Huang Z, Gao L, Ma H, Chang R (2024). Osteopontin/SPP1: a potential mediator between immune cells and vascular calcification. Front Immunol.

[60] Hohlstein P, Abu Jhaisha S, Yagmur E, Wawer D, Pollmanns MR, Adams JK (2023). Elevated Midkine Serum Levels Are Associated with Long-Term Survival in Critically Ill Patients. Int J Mol Sci.

[61] Liu G, Pan S, Xia H, Li M, Wu A (2024). The causal relationship between thoracic aortic aneurysm and immune cells: a mendelian randomization study. BMC Cardiovasc Disord.

[62] Di Gregorio J, Robuffo I, Spalletta S, Giambuzzi G, De Iuliis V, Toniato E (2020). The Epithelial-to-Mesenchymal Transition as a Possible Therapeutic Target in Fibrotic Disorders. Front Cell Dev Biol.

[63] Tseng TH, Chen CL, Chang CH, Wang JH, Young TH (2023). IL-6 induces periostin production in human ACL remnants: a possible mechanism causing post-traumatic osteoarthritis. J Orthop Surg Res.

[64] Palomer X, Salvador JM, Grinan-Ferre C, Barroso E, Pallas M, Vazquez-Carrera M (2024). GADD45A: With or without you. Med Res Rev.

[65] Hwang JH, Kim TH, Kim YH, Noh JR, Choi DH, Kim KS (2019). Gadd45beta promotes regeneration after injury through TGFbeta-dependent restitution in experimental colitis. Exp Mol Med.

[66] Du WW, Rafiq M, Yuan H, Li X, Wang S, Wu J A Novel Protein NAB1-356 Encoded by circRNA circNAB1 Mitigates Atrial Fibrillation by Reducing Inflammation and Fibrosis. Adv Sci (Weinh). 2025: e2411959.

[67] Xu X, Hirata H, Shiraki M, Kamohara A, Nishioka K, Miyamoto H (2019). Prostate transmembrane protein androgen induced 1 is induced by activation of osteoclasts and regulates bone resorption. FASEB J.

[68] Zhang L, Wang X, Lai C, Zhang H, Lai M (2019). PMEPA1 induces EMT via a non-canonical TGF-beta signalling in colorectal cancer. J Cell Mol Med.

[69] Liu B, Sun L, Liu Q, Gong C, Yao Y, Lv X (2015). A cytoplasmic NF-kappaB interacting long noncoding RNA blocks IkappaB phosphorylation and suppresses breast cancer metastasis. Cancer Cell.

[70] Wang SM, Hsu JC, Ko CY, Wu HE, Hsiao YW, Wang JM (2023). Astrocytic Cebpd Regulates Pentraxin 3 Expression to Promote Fibrotic Scar Formation After Spinal Cord Injury. Mol Neurobiol.

[71] Wang Y, Wang B, Cao W, Xu X (2024). PTX3 activates POSTN and promotes the progression of glioblastoma via the MAPK/ERK signalling axis. Biochem Biophys Res Commun.

[72] Luo Y, Luo J, An P, Zhao Y, Zhao W, Fang Z (2024). The activator protein-1 complex governs a vascular degenerative transcriptional programme in smooth muscle cells to trigger aortic dissection and rupture. Eur Heart J.

[73] Terauchi M, Kajiyama H, Yamashita M, Kato M, Tsukamoto H, Umezu T (2007). Possible involvement of TWIST in enhanced peritoneal metastasis of epithelial ovarian carcinoma. Clin Exp Metastasis.

[74] Liu YN, Kang BB, Chen JH (2004). Transcriptional regulation of human osteopontin promoter by C/EBPalpha and AML-1 in metastatic cancer cells. Oncogene.

[75] Duncan MK, Kozmik Z, Cveklova K, Piatigorsky J, Cvekl A (2000). Overexpression of PAX6(5a) in lens fiber cells results in cataract and upregulation of (alpha)5(beta)1 integrin expression. J Cell Sci.

[76] Kumar S, Gonzalez EA, Rameshwar P, Etchegaray JP (2020). Non-Coding RNAs as Mediators of Epigenetic Changes in Malignancies. Cancers (Basel).

[77] Boudreau NJ, Varner JA (2004). The homeobox transcription factor Hox D3 promotes integrin alpha5beta1 expression and function during angiogenesis. J Biol Chem.

[78] Yan Y, Tan MW, Xue X, Ding XY, Wang GK, Xu ZY (2016). Involvement of Oct4 in the pathogenesis of thoracic aortic dissection via inducing the dedifferentiated phenotype of human aortic smooth muscle cells by directly upregulating KLF5. J Thorac Cardiovasc Surg.

