# Supplementary material for: Single-cell Profiling Reveals Cooperative Participation of SPP1+ Myeloid and POSTN+ Fibroblast Subsets in Inflammation and Remodeling in Thoracic Aortic Aneurysm
Source: Int J Med Sci. 2026 Mar 4;23(4):1333–55. doi: 10.7150/ijms.124136 (PMC13048888; doi:10.7150/ijms.124136)
Supplement: Supplementary file 1 — Supplementary figures and tables. [file ijmsv23p1333s1.pdf]

SUPPLEMENTARY DATA

Single-cell Profiling Reveals Cooperative Participation of *SPP1*$^+$ Myeloid and *POSTN*$^+$ Fibroblast Subsets in Inflammation and Remodeling in Thoracic Aortic Aneurysm

Yingjiao Ju[1], Jingyi Yao[1], Song Zhang[1], Jiangxu Wu[2], Jiongao Xiang[3], Li Min[1,4*], Mingyuan Liu[3*]

Supplementary Figure 1. Batch effect assessment in integrated TAA single-cell datasets.

Supplementary Figure 2. Differential cell population proportions between control and disease groups.

Supplementary Figure 3. Top marker genes from all immune and non-immune subcellular clusters.

Supplementary Figure 4. Shared pathway enrichment and gene-level overlap between immune and stromal populations.

Supplementary Figure 5. Cell-cell communication in SPP1 and MK signaling networks between myeloid and fibroblast subpopulations in control and TAA groups.

Supplementary Figure 6. Enrichment plots (upper panels) and leading-edge gene expression heatmaps (lower panels) for representative pathways dysregulated in myeloid_c2-*SPP1* and fibroblast_c1-*POSTN* subpopulations in the TAA group compared with the control group.

Supplementary Figure 7. TF motif enrichment and regulon activity in myeloid and fibroblast subpopulations.

Supplementary Table 1. Specifically activated transcription factors in myeloid and fibroblast cell subpopulations

SupplementaryTable 2. DEGs in Myeloid_c2-SPP1 (TAA vs. Control) with NF-κB and EMT pathway enrichment

Supplementary Table 3. DEGs in Fibroblast_c1-POSTN (TAA vs. Control) with NF-κB and EMT pathway enrichment

Supplementary Table 4. Transcription factors predicted from 18 hub genes using TRRUST

Supplementary Table 5. Clinical characteristics of samples used for spatial transcriptomics and multiplex immunofluorescence

Supplementary Figure 1

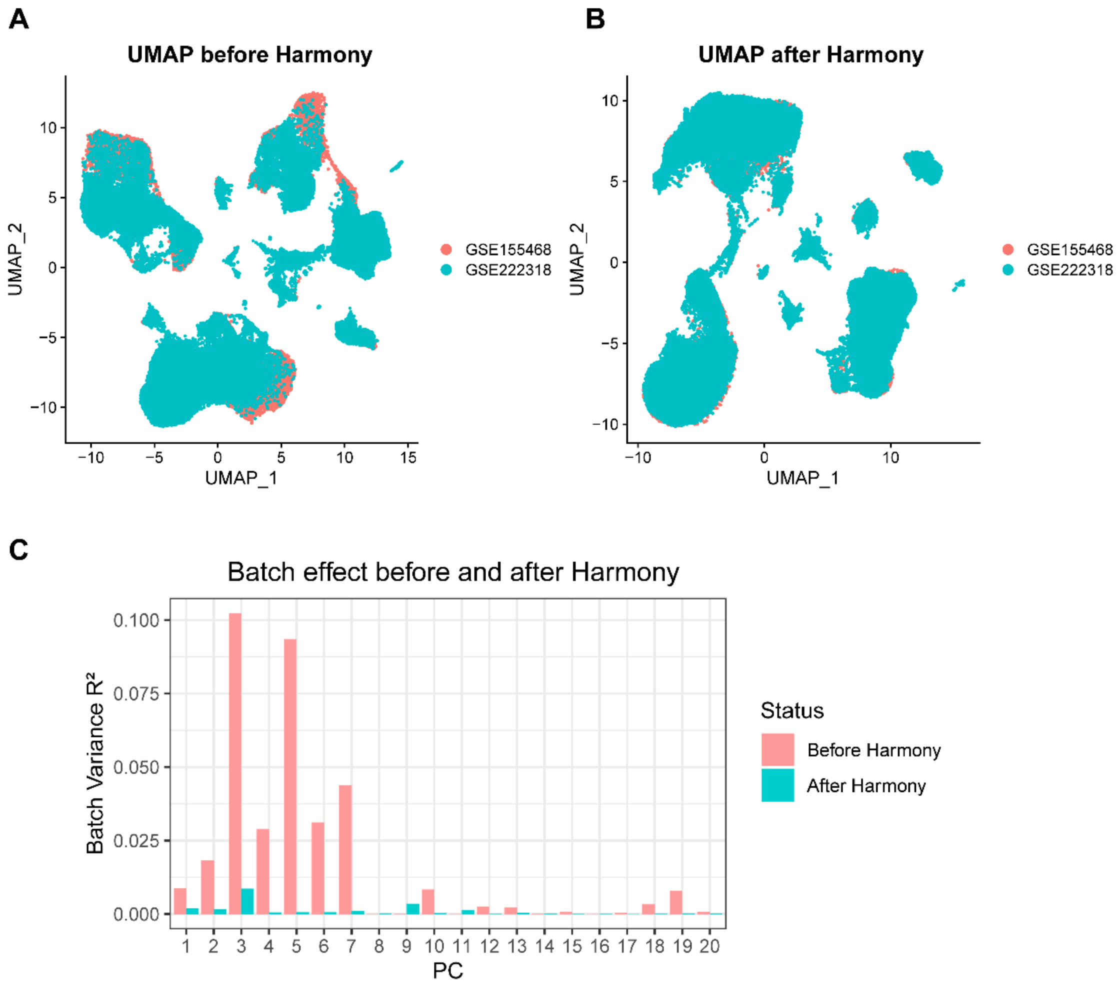

Supplementary Figure 1. Batch effect assessment in integrated TAA single-cell datasets. A, **B**, UMAP visualization of cells colored by dataset before (A) and after (B) Harmony integration. **C**, Quantitative assessment of batch effects across the first 20 principal components, showing the proportion of variance explained by batch before and after Harmony integration.

Supplementary Figure 2

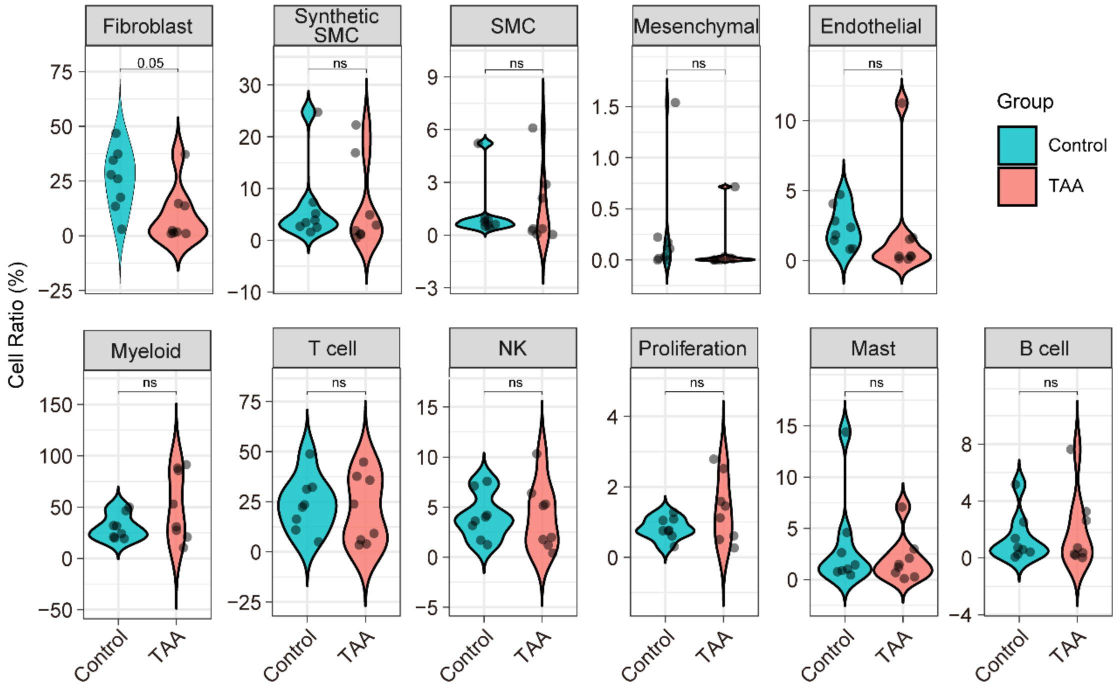

Supplementary Figure 2. Differential cell population proportions between control and disease groups. Violin plots showing that fibroblasts were significantly decreased in the TAA group compared to the control group. In contrast, myeloid and other cell types exhibited a trend toward change, but the differences were not statistically significant. Statistical analysis was performed using the Wilcoxon rank-sum test.

Supplementary Figure 3

A

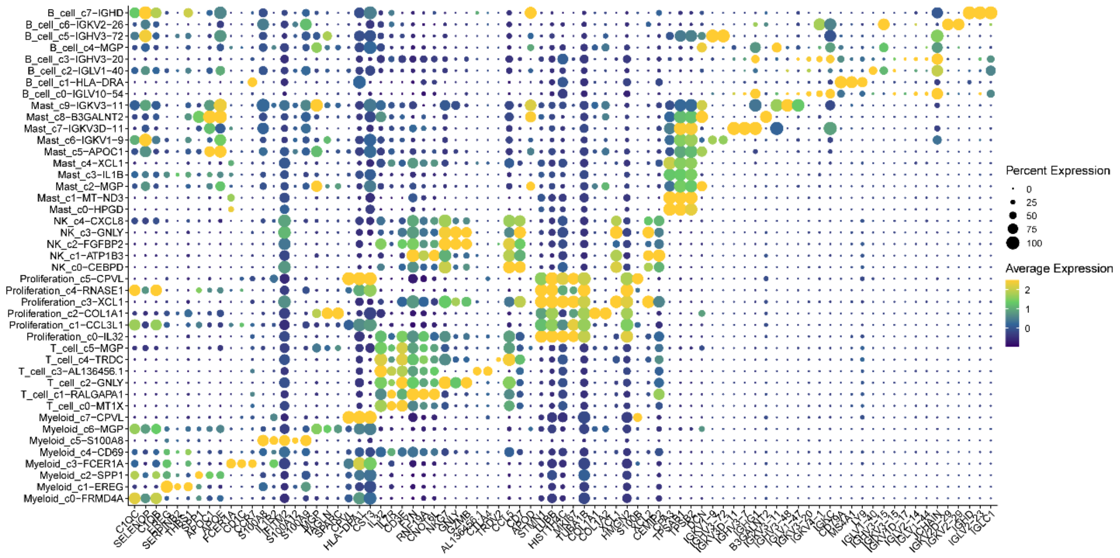

B

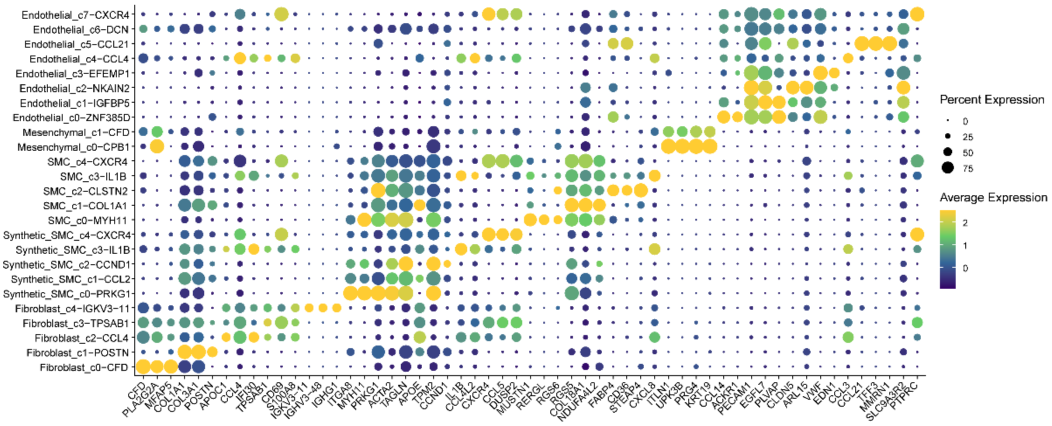

Supplementary Figure 3. Top marker genes from all immune and non-imm une subcellular clusters. A, **B**, Dot plots showing the expression of the top t hree marker genes for 43 sub-immune cell clusters (A) and 25 sub-non-immun e cell clusters (B).

Supplementary Figure 4

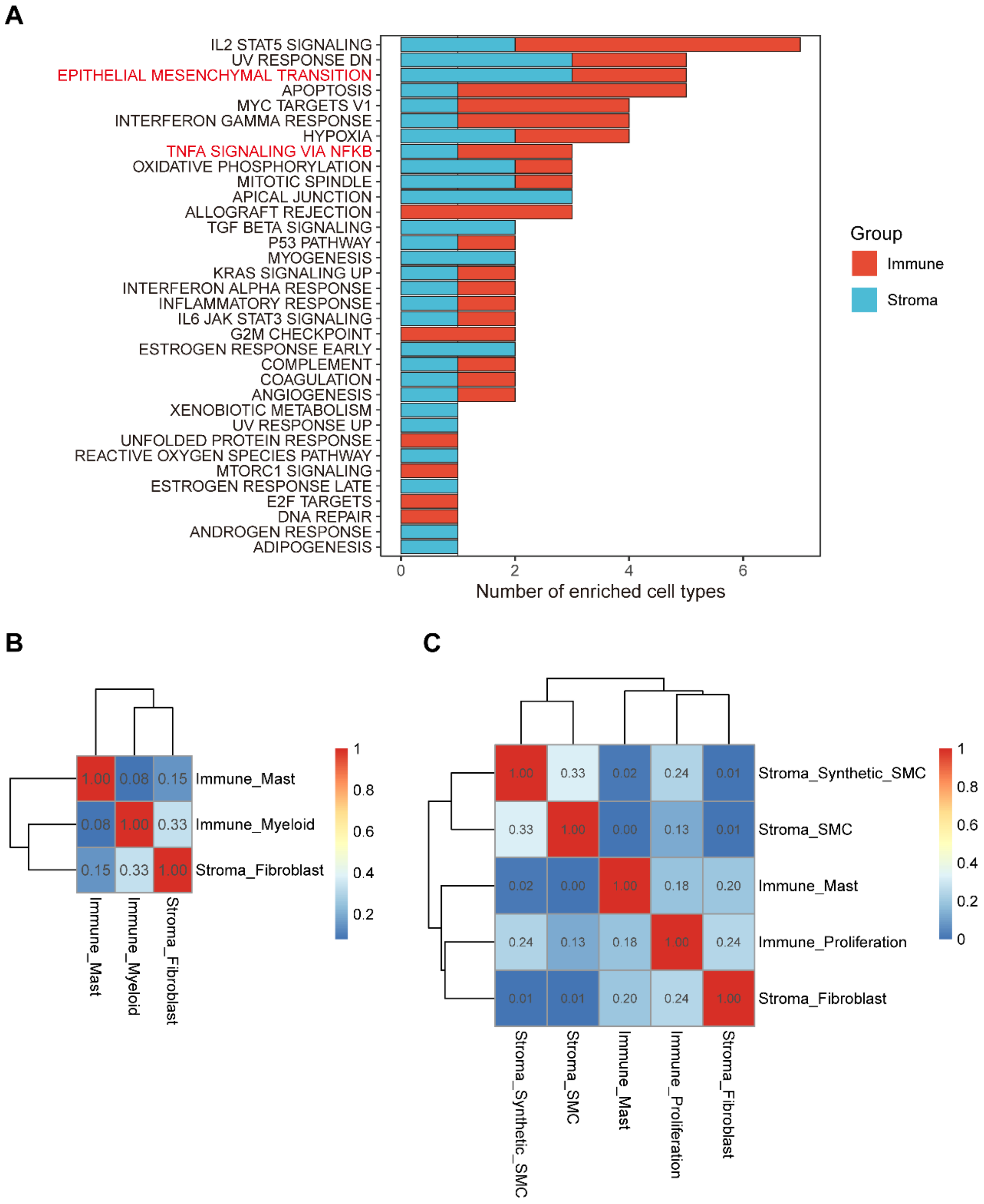

Supplementary Figure 4. Shared pathway enrichment and gene-level overla p between immune and stromal populations. **A**, Number of cell types with s ignificant enrichment (adjusted $p < 0.05$) for each pathway in immune versus s tromal populations. **B**, Jaccard similarity of genes driving TNF-α signaling via NF-κB across cell types. **C**, Jaccard similarity of genes driving epithelial mese nchymal transition (EMT) across cell types.

Supplementary Figure 5

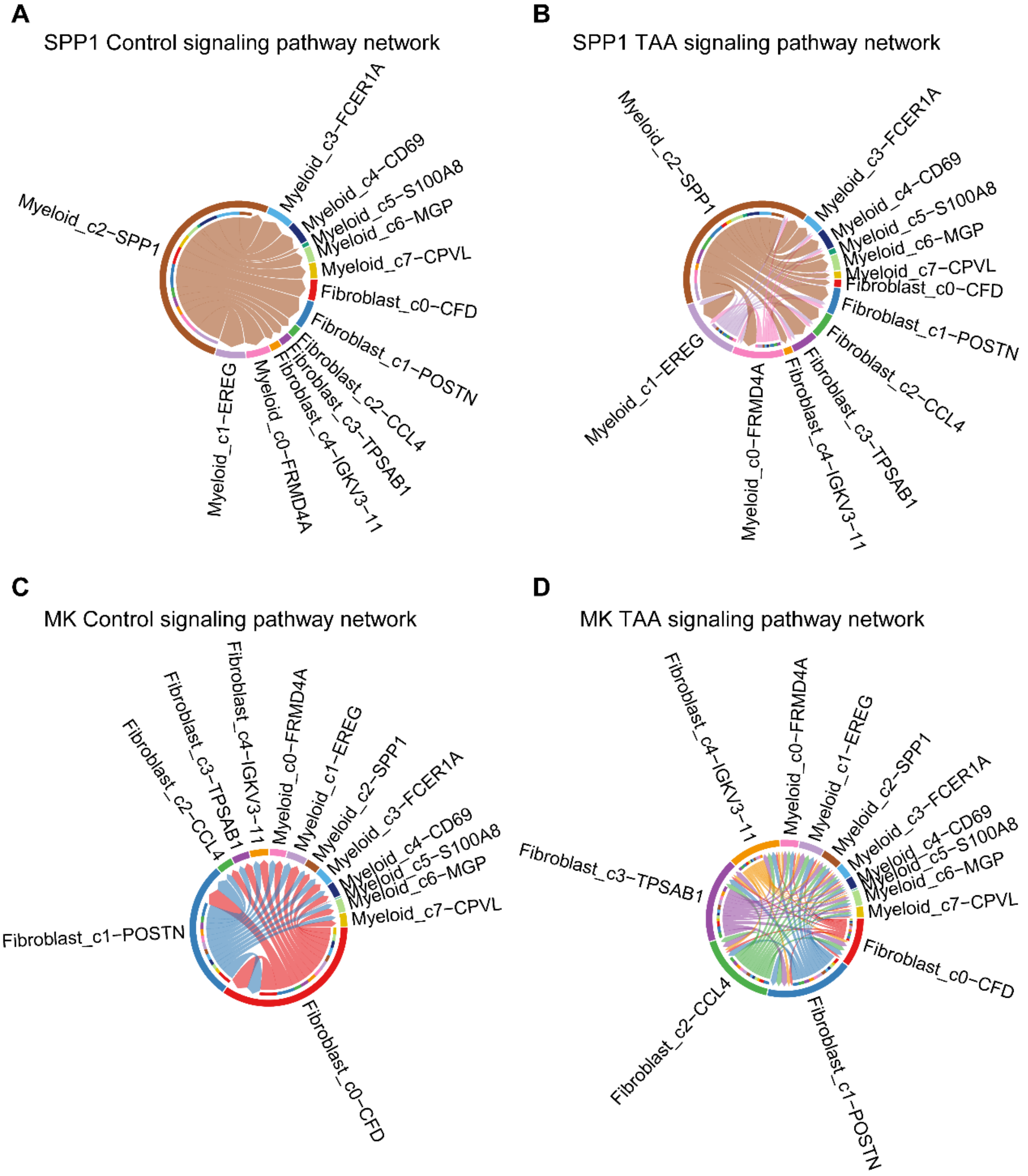

Supplementary Figure 5. Cell-cell communication in SPP1 and MK signaling networks between myeloid and fibroblast subpopulations in control and TAA groups. A-B, Circos plots of the SPP1 signaling pathway network in the control (A) and TAA (B) groups. **C-D**, Circos plots of the MK signaling pathway network in the control (C) and TAA (D) groups.

Supplementary Figure 6

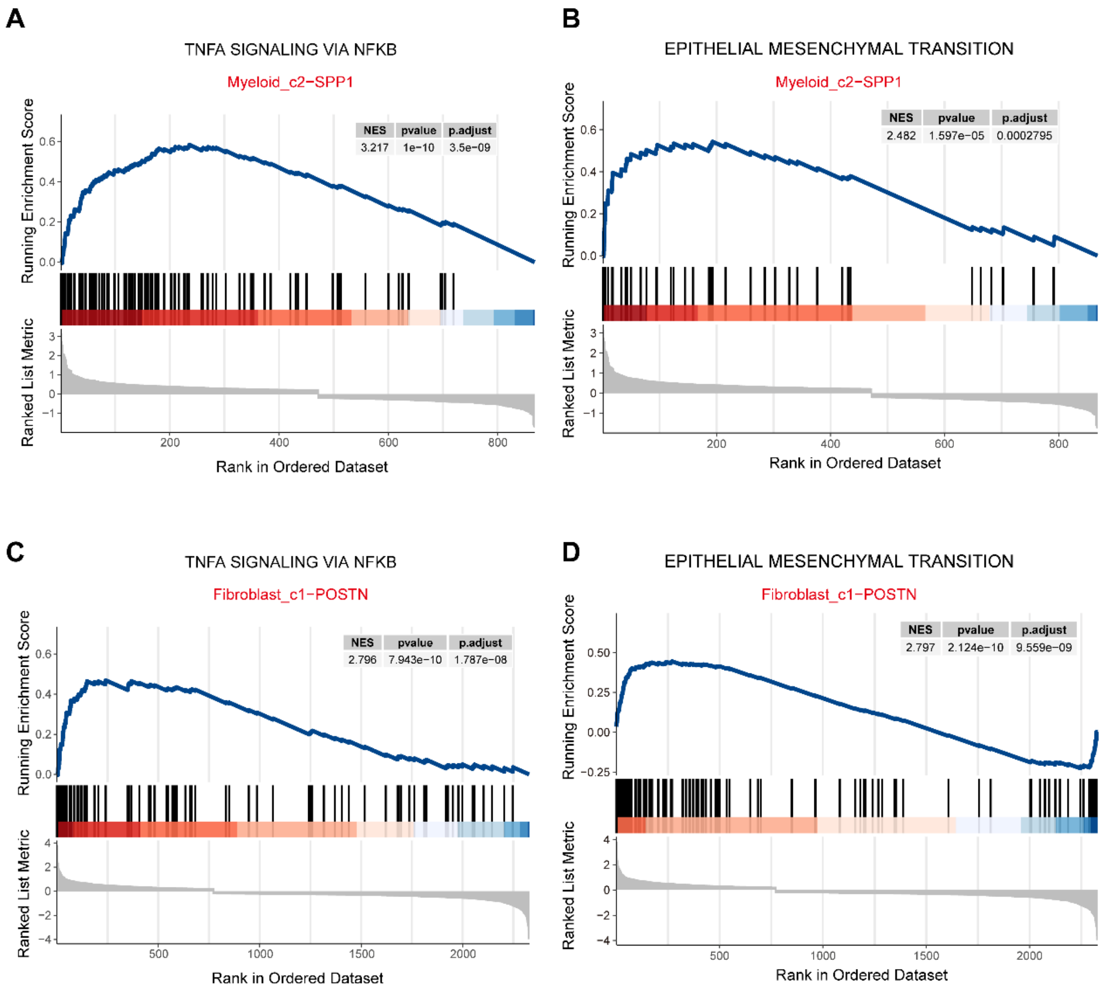

Supplementary Figure 6. Enrichment plots (upper panels) and leading-edge gene expression heatmaps (lower panels) for representative pathways dysregulated in myeloid_c2-*SPP1* and fibroblast_c1-*POSTN* subpopulations in the TAA group compared with the control group. NES, normalized enrichment score. Vertical lines in the enrichment plots indicate the positions of gene set members in the ranked gene list, while the heatmaps show average expression levels of leading-edge genes. Adjusted P values were calculated using the BH method. **A**, Enrichment plot and heatmap for TNFα signaling via NF-κB in myeloid_c2-*SPP1*. **B**, Enrichment plot and heatmap for epithelial mesenchymal transition in myeloid_c2-*SPP1*. **C**, Enrichment plot and heatmap for TNFα signaling via NF-κB in fibroblast_c1-*POSTN*. **D**, Enrichment plot and heatmap for epithelial mesenchymal transition in fibroblast_c1-*POSTN*.

Supplementary Figure 7

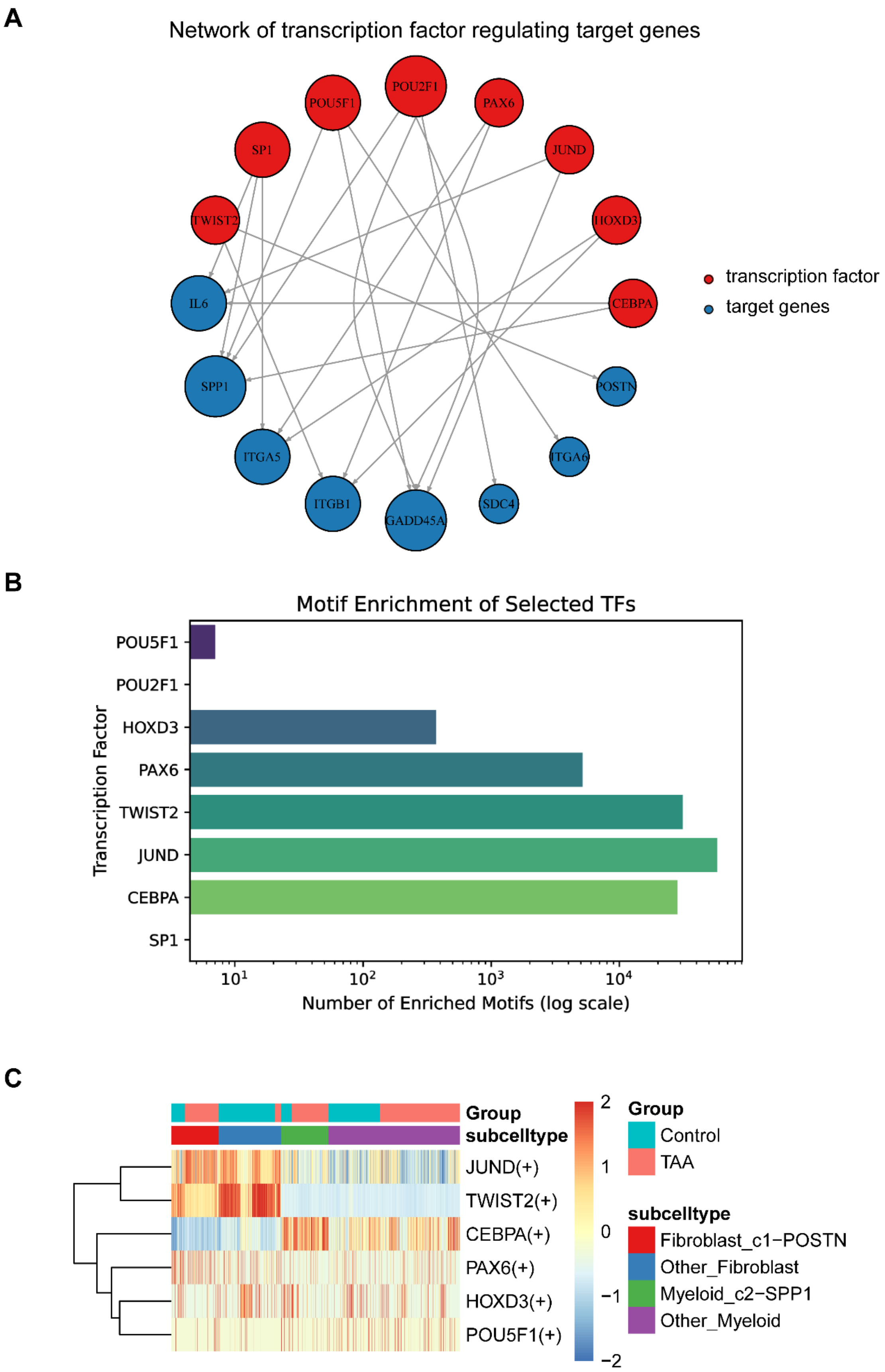

Supplementary Figure 7. TF motif enrichment and regulon activity in myeloid and fibroblast subpopulations. A, TF–target regulatory network from TRRUST. Red nodes: TFs; blue nodes: targets; node size: interaction count; arrows: regulatory direction. B, Number of enriched motifs for selected TFs identified by pySCENIC. C, Heatmap of scaled AUCell regulon activity across subpopulations and groups (Control vs. TAA).

Supplementary Table 1. Specifically activated transcription factors in myeloid and fibroblast cell subpopulations

Transcription factor	p_val	avg_log2FC	pct.1	pct.2	p_val_adj	cluster
HNF4A	3.40E-44	2.64E+00	0.87	0.181	9.23E-42	Fibroblast_c0-CFD
TFAP2C	2.50E-40	2.62E+00	0.94	0.32	6.77E-38	Fibroblast_c0-CFD
SMAD3	1.53E-38	2.88E+00	0.79	0.182	4.15E-36	Fibroblast_c0-CFD
TFAP2A	1.61E-37	2.67E+00	0.66	0.097	4.35E-35	Fibroblast_c0-CFD
FOXM1	5.44E-36	2.04E+00	0.55	0.088	1.47E-33	Fibroblast_c0-CFD
ESR1	1.30E-33	2.86E+00	0.26	0.036	3.52E-31	Fibroblast_c0-CFD
FOXA1	1.42E-30	1.92E+00	0.87	0.373	3.84E-28	Fibroblast_c0-CFD
NFIC	1.98E-26	1.47E+00	0.68	0.205	5.36E-24	Fibroblast_c0-CFD
SRF	2.12E-25	1.48E+00	0.83	0.363	5.75E-23	Fibroblast_c0-CFD
KLF3	2.19E-22	1.75E+00	0.48	0.102	5.94E-20	Fibroblast_c0-CFD
NR3C1	2.49E-22	1.05E+00	0.98	0.824	6.75E-20	Fibroblast_c0-CFD
STAT4	2.42E-20	1.02E+00	0.57	0.21	6.57E-18	Fibroblast_c0-CFD
ATF6	4.74E-20	1.51E+00	0.58	0.242	1.29E-17	Fibroblast_c0-CFD
E2F7	2.58E-19	1.43E+00	0.52	0.17	6.99E-17	Fibroblast_c0-CFD
PAX6	1.05E-18	9.92E-01	0.91	0.527	2.84E-16	Fibroblast_c0-CFD
NFKB2	8.64E-17	1.47E+00	0.93	0.688	2.34E-14	Fibroblast_c0-CFD
FOXO3	5.02E-16	1.44E+00	0.31	0.083	1.36E-13	Fibroblast_c0-CFD
CEBPG	2.43E-15	1.27E+00	0.29	0.096	6.57E-13	Fibroblast_c0-CFD
SMAD4	2.50E-15	1.64E+00	0.5	0.166	6.78E-13	Fibroblast_c0-CFD
MNT	3.06E-15	1.47E+00	1	0.932	8.30E-13	Fibroblast_c0-CFD
MYBL2	5.95E-15	1.08E+00	0.28	0.12	1.61E-12	Fibroblast_c0-CFD
LEF1	1.98E-13	1.20E+00	0.48	0.199	5.35E-11	Fibroblast_c0-CFD
MYB	5.39E-13	8.85E-01	0.35	0.137	1.46E-10	Fibroblast_c0-CFD
YY1	6.74E-13	1.44E+00	0.68	0.39	1.83E-10	Fibroblast_c0-CFD
SMAD5	1.81E-12	9.39E-01	0.38	0.161	4.91E-10	Fibroblast_c0-CFD
MITF	2.10E-12	6.83E-01	0.27	0.135	5.70E-10	Fibroblast_c0-CFD
CREB3L1	1.27E-11	6.96E-01	0.33	0.141	3.43E-09	Fibroblast_c0-CFD
NANOG	1.08E-10	3.36E-01	0.28	0.108	2.93E-08	Fibroblast_c0-CFD
E2F5	5.95E-10	1.32E+00	0.36	0.115	1.61E-07	Fibroblast_c0-CFD
SOX10	1.09E-09	7.27E-01	0.37	0.15	2.96E-07	Fibroblast_c0-CFD
ETV4	2.39E-09	8.16E-01	0.57	0.292	6.47E-07	Fibroblast_c0-CFD
ATF2	3.00E-09	3.30E-01	0.44	0.218	8.14E-07	Fibroblast_c0-CFD
PPARA	5.47E-09	9.37E-01	0.64	0.426	1.48E-06	Fibroblast_c0-CFD
FOXO4	1.36E-08	1.55E+00	0.36	0.14	3.68E-06	Fibroblast_c0-CFD
SOX11	1.38E-08	8.51E-01	0.31	0.13	3.75E-06	Fibroblast_c0-CFD
BHLHE40	1.81E-08	5.62E-01	1	0.901	4.91E-06	Fibroblast_c0-CFD
ARNTL	8.51E-08	2.12E+00	0.33	0.138	2.31E-05	Fibroblast_c0-CFD
BCL6	1.18E-07	6.69E-01	1	0.938	3.20E-05	Fibroblast_c0-CFD
ELK4	2.40E-07	6.76E-01	0.27	0.144	6.50E-05	Fibroblast_c0-CFD
LHX2	4.52E-07	4.06E-01	0.3	0.161	1.22E-04	Fibroblast_c0-CFD
HNF1A	1.50E-06	4.40E-01	0.99	0.988	4.07E-04	Fibroblast_c0-CFD

Transcription factor	p_val	avg_log2FC	pct.1	pct.2	p_val_adj	cluster
ZNF24	2.83E-06	4.90E-01	0.95	0.844	7.67E-04	Fibroblast_c0-CFD
ZNF263	6.25E-06	7.41E-01	1	0.993	1.69E-03	Fibroblast_c0-CFD
ZNF274	1.10E-05	6.32E-01	1	0.945	2.99E-03	Fibroblast_c0-CFD
RXRA	1.44E-05	6.86E-01	0.65	0.422	3.90E-03	Fibroblast_c0-CFD
GATA2	4.44E-05	5.31E-01	0.59	0.362	1.20E-02	Fibroblast_c0-CFD
FLI1	5.24E-05	5.80E-01	0.65	0.442	1.42E-02	Fibroblast_c0-CFD
FOXA2	5.79E-05	2.56E-01	0.75	0.736	1.57E-02	Fibroblast_c0-CFD
HHEX	7.01E-05	6.18E-01	0.94	0.862	1.90E-02	Fibroblast_c0-CFD
SOX2	1.21E-04	7.50E-01	0.26	0.073	3.29E-02	Fibroblast_c0-CFD
RARA	2.03E-04	1.18E+00	0.48	0.29	5.51E-02	Fibroblast_c0-CFD
PGR	7.90E-04	4.68E-01	0.3	0.195	2.14E-01	Fibroblast_c0-CFD
KMT2A	8.54E-04	3.86E-01	0.6	0.367	2.31E-01	Fibroblast_c0-CFD
TEAD1	5.52E-34	4.10E+00	0.39	0.059	1.50E-31	Fibroblast_c1-POSTN
PBX3	3.04E-16	3.32E+00	0.27	0.037	8.25E-14	Fibroblast_c1-POSTN
POU5F1	1.04E-24	3.25E+00	0.26	0.066	2.81E-22	Fibroblast_c1-POSTN
NANOG	1.37E-11	2.87E+00	0.32	0.105	3.70E-09	Fibroblast_c1-POSTN
TCF4	7.16E-22	2.87E+00	0.27	0.043	1.94E-19	Fibroblast_c1-POSTN
SOX11	1.51E-17	2.80E+00	0.45	0.118	4.09E-15	Fibroblast_c1-POSTN
HOXB13	1.31E-15	2.78E+00	0.26	0.074	3.56E-13	Fibroblast_c1-POSTN
LHX2	2.89E-18	2.19E+00	0.53	0.142	7.84E-16	Fibroblast_c1-POSTN
BHLHE40	5.18E-20	2.06E+00	1	0.901	1.40E-17	Fibroblast_c1-POSTN
MEIS1	5.27E-27	1.99E+00	0.29	0.052	1.43E-24	Fibroblast_c1-POSTN
KLF3	7.32E-15	1.94E+00	0.36	0.112	1.98E-12	Fibroblast_c1-POSTN
NFKB2	1.11E-26	1.88E+00	1	0.682	3.01E-24	Fibroblast_c1-POSTN
SMAD3	9.99E-23	1.69E+00	0.58	0.199	2.71E-20	Fibroblast_c1-POSTN
ATF7	1.79E-13	1.62E+00	0.29	0.087	4.86E-11	Fibroblast_c1-POSTN
NFIC	5.88E-17	1.56E+00	0.58	0.213	1.59E-14	Fibroblast_c1-POSTN
SMAD5	8.03E-12	1.56E+00	0.34	0.164	2.18E-09	Fibroblast_c1-POSTN
MYBL2	4.63E-16	1.54E+00	0.4	0.11	1.25E-13	Fibroblast_c1-POSTN
FOXM1	2.32E-17	1.51E+00	0.28	0.111	6.30E-15	Fibroblast_c1-POSTN
SOX2	2.31E-19	1.49E+00	0.34	0.067	6.27E-17	Fibroblast_c1-POSTN
SRF	3.38E-15	1.41E+00	0.77	0.368	9.16E-13	Fibroblast_c1-POSTN
NCOA2	2.13E-08	1.39E+00	0.26	0.098	5.78E-06	Fibroblast_c1-POSTN
CREB3L1	1.87E-08	1.37E+00	0.32	0.142	5.06E-06	Fibroblast_c1-POSTN
E2F7	9.33E-11	1.21E+00	0.4	0.18	2.53E-08	Fibroblast_c1-POSTN
ONECUT1	1.10E-08	1.19E+00	0.31	0.108	2.98E-06	Fibroblast_c1-POSTN
TFAP2C	7.97E-23	1.19E+00	0.81	0.331	2.16E-20	Fibroblast_c1-POSTN
CUX1	9.86E-11	1.15E+00	0.3	0.103	2.67E-08	Fibroblast_c1-POSTN
KMT2A	3.30E-14	1.12E+00	0.71	0.358	8.96E-12	Fibroblast_c1-POSTN
PAX6	4.28E-22	1.05E+00	0.93	0.526	1.16E-19	Fibroblast_c1-POSTN
BCL6	4.89E-11	1.01E+00	1	0.938	1.33E-08	Fibroblast_c1-POSTN
ZNF24	4.75E-16	9.60E-01	0.98	0.842	1.29E-13	Fibroblast_c1-POSTN
KLF5	1.90E-15	9.42E-01	0.27	0.11	5.15E-13	Fibroblast_c1-POSTN

Transcription factor	p_val	avg_log2FC	pct.1	pct.2	p_val_adj	cluster
ATF3	1.15E-06	9.28E-01	1	0.872	3.12E-04	Fibroblast_c1-POSTN
ELK4	3.33E-04	8.43E-01	0.25	0.146	9.01E-02	Fibroblast_c1-POSTN
YY1	4.93E-06	8.31E-01	0.58	0.398	1.34E-03	Fibroblast_c1-POSTN
NR3C1	4.23E-13	7.50E-01	0.98	0.824	1.15E-10	Fibroblast_c1-POSTN
TFAP2A	3.39E-15	7.14E-01	0.33	0.124	9.20E-13	Fibroblast_c1-POSTN
TFDP1	6.57E-08	5.96E-01	0.58	0.282	1.78E-05	Fibroblast_c1-POSTN
ATF6	3.85E-06	4.73E-01	0.38	0.258	1.04E-03	Fibroblast_c1-POSTN
LEF1	5.73E-07	4.58E-01	0.42	0.204	1.55E-04	Fibroblast_c1-POSTN
FOXA1	1.74E-07	2.58E-01	0.62	0.394	4.70E-05	Fibroblast_c1-POSTN
SRF	1.85E-23	1.46E+00	0.9	0.358	5.02E-21	Fibroblast_c2-CCL4
SMAD3	3.64E-18	1.03E+00	0.51	0.205	9.87E-16	Fibroblast_c2-CCL4
FOXA1	5.62E-17	9.04E-01	0.83	0.377	1.52E-14	Fibroblast_c2-CCL4
BCL6	5.74E-15	1.39E+00	0.98	0.94	1.56E-12	Fibroblast_c2-CCL4
HNF4A	9.04E-15	9.90E-01	0.54	0.208	2.45E-12	Fibroblast_c2-CCL4
FOXP1	1.19E-14	6.50E-01	0.98	0.946	3.23E-12	Fibroblast_c2-CCL4
NFIC	2.05E-14	7.30E-01	0.44	0.225	5.55E-12	Fibroblast_c2-CCL4
HMBOX1	4.21E-14	1.36E+00	0.95	0.876	1.14E-11	Fibroblast_c2-CCL4
HBP1	6.79E-14	1.94E+00	0.98	0.93	1.84E-11	Fibroblast_c2-CCL4
MNT	3.69E-13	9.48E-01	0.99	0.933	1.00E-10	Fibroblast_c2-CCL4
NR5A1	7.59E-13	2.99E-01	1	0.999	2.06E-10	Fibroblast_c2-CCL4
ZEB2	1.16E-12	1.44E+00	0.97	0.912	3.16E-10	Fibroblast_c2-CCL4
TFAP2C	1.45E-12	4.94E-01	0.63	0.346	3.94E-10	Fibroblast_c2-CCL4
NR2F6	7.71E-12	1.02E+00	0.99	0.91	2.09E-09	Fibroblast_c2-CCL4
SMAD4	3.38E-11	6.05E-01	0.42	0.172	9.16E-09	Fibroblast_c2-CCL4
SP3	9.70E-11	1.20E+00	0.46	0.258	2.63E-08	Fibroblast_c2-CCL4
ZNF639	1.07E-10	1.02E+00	0.95	0.885	2.89E-08	Fibroblast_c2-CCL4
E2F6	2.84E-10	9.07E-01	0.96	0.958	7.70E-08	Fibroblast_c2-CCL4
ATF2	1.14E-09	1.10E+00	0.42	0.219	3.09E-07	Fibroblast_c2-CCL4
FOSL1	3.84E-09	1.33E+00	0.4	0.126	1.04E-06	Fibroblast_c2-CCL4
MXI1	6.46E-09	8.51E-01	0.97	0.965	1.75E-06	Fibroblast_c2-CCL4
SNAI2	9.53E-09	8.20E-01	1	0.959	2.58E-06	Fibroblast_c2-CCL4
CDX2	5.56E-08	6.79E-01	0.95	0.926	1.51E-05	Fibroblast_c2-CCL4
HNF1A	5.56E-08	4.50E-01	0.97	0.989	1.51E-05	Fibroblast_c2-CCL4
ZBTB7A	2.44E-07	5.48E-01	0.97	0.971	6.62E-05	Fibroblast_c2-CCL4
MBD1	3.11E-07	7.07E-01	0.99	0.98	8.43E-05	Fibroblast_c2-CCL4
THAP11	1.55E-06	5.77E-01	0.97	0.975	4.19E-04	Fibroblast_c2-CCL4
NR2F2	2.74E-06	2.66E-01	0.95	0.918	7.43E-04	Fibroblast_c2-CCL4
PPARA	4.12E-06	6.05E-01	0.61	0.428	1.12E-03	Fibroblast_c2-CCL4
TGIF2	4.57E-06	5.40E-01	0.96	0.888	1.24E-03	Fibroblast_c2-CCL4
ZNF24	1.06E-05	3.42E-01	0.99	0.841	2.89E-03	Fibroblast_c2-CCL4
NR3C1	1.08E-05	5.27E-01	0.97	0.825	2.94E-03	Fibroblast_c2-CCL4
ETV4	2.22E-05	7.82E-01	0.48	0.299	6.03E-03	Fibroblast_c2-CCL4
ZNF274	9.86E-05	2.60E-01	0.98	0.947	2.67E-02	Fibroblast_c2-CCL4

Transcription factor	p_val	avg_log2FC	pct.1	pct.2	p_val_adj	cluster
PAX6	1.51E-04	4.38E-01	0.78	0.538	4.10E-02	Fibroblast_c2-CCL4
JUND	2.40E-04	6.16E-01	0.3	0.182	6.51E-02	Fibroblast_c2-CCL4
STAT6	2.70E-04	3.27E-01	0.67	0.44	7.31E-02	Fibroblast_c2-CCL4
FLI1	4.25E-04	3.65E-01	0.59	0.447	1.15E-01	Fibroblast_c2-CCL4
ESR2	4.53E-04	3.90E-01	0.34	0.24	1.23E-01	Fibroblast_c2-CCL4
FOXP2	1.76E-03	2.90E-01	0.89	0.873	4.78E-01	Fibroblast_c2-CCL4
FOSL2	2.96E-03	4.83E-01	0.43	0.242	8.03E-01	Fibroblast_c2-CCL4
SRF	8.43E-18	1.31E+00	0.79	0.367	2.28E-15	Fibroblast_c3-TPSAB1
SMAD3	3.90E-17	5.84E-01	0.49	0.207	1.06E-14	Fibroblast_c3-TPSAB1
NFIC	4.66E-13	9.50E-01	0.45	0.224	1.26E-10	Fibroblast_c3-TPSAB1
FOXA1	5.06E-12	7.42E-01	0.69	0.388	1.37E-09	Fibroblast_c3-TPSAB1
SMAD4	5.88E-12	1.02E+00	0.39	0.175	1.59E-09	Fibroblast_c3-TPSAB1
TFAP2C	9.33E-12	8.13E-01	0.63	0.346	2.53E-09	Fibroblast_c3-TPSAB1
ATF2	7.66E-11	1.34E+00	0.42	0.219	2.08E-08	Fibroblast_c3-TPSAB1
MITF	4.26E-09	1.09E+00	0.31	0.132	1.15E-06	Fibroblast_c3-TPSAB1
HNF4A	4.35E-09	3.60E-01	0.43	0.218	1.18E-06	Fibroblast_c3-TPSAB1
STAT4	9.36E-09	1.80E+00	0.41	0.223	2.54E-06	Fibroblast_c3-TPSAB1
NR2F6	1.78E-08	7.40E-01	0.99	0.91	4.82E-06	Fibroblast_c3-TPSAB1
STAT6	6.01E-08	7.21E-01	0.66	0.441	1.63E-05	Fibroblast_c3-TPSAB1
MNT	2.49E-07	4.55E-01	0.98	0.934	6.76E-05	Fibroblast_c3-TPSAB1
ZNF24	3.35E-07	5.42E-01	0.96	0.843	9.07E-05	Fibroblast_c3-TPSAB1
HBP1	5.99E-07	1.05E+00	0.96	0.932	1.62E-04	Fibroblast_c3-TPSAB1
RUNX3	6.54E-07	9.78E-01	0.61	0.407	1.77E-04	Fibroblast_c3-TPSAB1
PPARA	9.59E-07	6.81E-01	0.63	0.427	2.60E-04	Fibroblast_c3-TPSAB1
KMT2A	1.36E-06	4.83E-01	0.45	0.379	3.67E-04	Fibroblast_c3-TPSAB1
E2F7	1.98E-06	9.75E-01	0.39	0.181	5.37E-04	Fibroblast_c3-TPSAB1
NR3C1	2.77E-06	5.46E-01	0.95	0.827	7.52E-04	Fibroblast_c3-TPSAB1
HMBOX1	1.13E-05	7.32E-01	0.96	0.875	3.06E-03	Fibroblast_c3-TPSAB1
MXI1	2.15E-05	5.46E-01	0.98	0.964	5.81E-03	Fibroblast_c3-TPSAB1
ZEB2	2.71E-05	1.25E+00	0.95	0.913	7.34E-03	Fibroblast_c3-TPSAB1
FOXP1	4.66E-05	4.30E-01	0.98	0.946	1.26E-02	Fibroblast_c3-TPSAB1
BCL6	8.26E-05	8.11E-01	0.99	0.939	2.24E-02	Fibroblast_c3-TPSAB1
ZNF639	3.25E-04	6.96E-01	0.95	0.885	8.81E-02	Fibroblast_c3-TPSAB1
SNAI2	7.35E-04	4.57E-01	0.98	0.961	1.99E-01	Fibroblast_c3-TPSAB1
NFATC2	7.92E-04	3.40E-01	0.92	0.76	2.15E-01	Fibroblast_c3-TPSAB1
TFDP1	1.02E-03	2.84E-01	0.37	0.299	2.76E-01	Fibroblast_c3-TPSAB1
E2F6	1.70E-03	5.39E-01	0.97	0.958	4.60E-01	Fibroblast_c3-TPSAB1
ZBTB7A	1.74E-03	3.24E-01	0.97	0.971	4.71E-01	Fibroblast_c3-TPSAB1
PAX6	5.07E-03	2.98E-01	0.75	0.541	1.00E+00	Fibroblast_c3-TPSAB1
LEF1	6.28E-03	3.52E-01	0.3	0.214	1.00E+00	Fibroblast_c3-TPSAB1
JUND	9.77E-03	3.52E-01	0.26	0.186	1.00E+00	Fibroblast_c3-TPSAB1
SMAD3	7.67E-16	3.02E-01	0.42	0.212	2.08E-13	Fibroblast_c4-IGKV3-11
NFIC	2.80E-15	8.86E-01	0.53	0.218	7.58E-13	Fibroblast_c4-IGKV3-11

Transcription factor	p_val	avg_log2FC	pct.1	pct.2	p_val_adj	cluster
SRF	5.54E-15	8.36E-01	0.73	0.372	1.50E-12	Fibroblast_c4-IGKV3-11
NFKB2	6.14E-12	7.92E-01	0.95	0.687	1.66E-09	Fibroblast_c4-IGKV3-11
FOXM1	9.37E-12	1.03E+00	0.27	0.112	2.54E-09	Fibroblast_c4-IGKV3-11
FOXA1	1.21E-11	9.57E-01	0.69	0.388	3.29E-09	Fibroblast_c4-IGKV3-11
HNF4A	2.65E-11	4.20E-01	0.36	0.223	7.18E-09	Fibroblast_c4-IGKV3-11
NR3C1	4.66E-11	7.51E-01	0.97	0.825	1.26E-08	Fibroblast_c4-IGKV3-11
LHX2	2.89E-09	1.23E+00	0.34	0.158	7.83E-07	Fibroblast_c4-IGKV3-11
TFAP2C	3.24E-09	3.90E-01	0.63	0.346	8.79E-07	Fibroblast_c4-IGKV3-11
SMAD4	5.66E-08	6.32E-01	0.26	0.186	1.53E-05	Fibroblast_c4-IGKV3-11
NANOG	4.21E-07	1.40E+00	0.27	0.109	1.14E-04	Fibroblast_c4-IGKV3-11
MNT	8.56E-07	3.49E-01	0.99	0.933	2.32E-04	Fibroblast_c4-IGKV3-11
ZNF274	1.56E-06	3.88E-01	0.99	0.946	4.22E-04	Fibroblast_c4-IGKV3-11
SNAI2	8.22E-05	3.37E-01	1	0.959	2.23E-02	Fibroblast_c4-IGKV3-11
SOX11	8.27E-05	9.96E-01	0.3	0.131	2.24E-02	Fibroblast_c4-IGKV3-11
KLF3	2.03E-04	8.42E-01	0.27	0.12	5.50E-02	Fibroblast_c4-IGKV3-11
E2F6	5.18E-04	5.27E-01	0.99	0.956	1.40E-01	Fibroblast_c4-IGKV3-11
RXRA	5.59E-04	5.54E-01	0.6	0.426	1.51E-01	Fibroblast_c4-IGKV3-11
HMBOX1	6.22E-04	5.10E-01	0.93	0.877	1.69E-01	Fibroblast_c4-IGKV3-11
ZNF263	9.62E-04	3.05E-01	1	0.993	2.61E-01	Fibroblast_c4-IGKV3-11
NR2F6	1.06E-03	6.01E-01	0.97	0.912	2.88E-01	Fibroblast_c4-IGKV3-11
ZNF24	1.09E-03	2.82E-01	0.96	0.843	2.95E-01	Fibroblast_c4-IGKV3-11
E2F7	3.36E-03	4.81E-01	0.26	0.192	9.12E-01	Fibroblast_c4-IGKV3-11
KMT2A	4.10E-03	4.25E-01	0.41	0.382	1.00E+00	Fibroblast_c4-IGKV3-11
SPI1	1.58E-30	2.74E+00	0.62	0.139	4.28E-28	Myeloid_c0-FRMD4A
LYL1	1.59E-29	1.25E+00	0.3	0.067	4.31E-27	Myeloid_c0-FRMD4A
MAFB	1.65E-28	4.18E+00	0.39	0.059	4.47E-26	Myeloid_c0-FRMD4A
IKZF1	1.26E-25	1.94E+00	0.47	0.135	3.42E-23	Myeloid_c0-FRMD4A
ELF3	2.50E-24	2.15E+00	0.29	0.072	6.77E-22	Myeloid_c0-FRMD4A
RBPJ	3.89E-23	3.66E-01	0.41	0.102	1.05E-20	Myeloid_c0-FRMD4A
BATF	3.97E-23	1.29E+00	0.38	0.119	1.08E-20	Myeloid_c0-FRMD4A
NFE2	4.24E-22	2.90E+00	0.26	0.045	1.15E-19	Myeloid_c0-FRMD4A
HNF4G	1.47E-21	2.29E+00	0.34	0.073	3.99E-19	Myeloid_c0-FRMD4A
ELF1	5.79E-19	9.29E-01	0.27	0.075	1.57E-16	Myeloid_c0-FRMD4A
PPARG	2.47E-18	1.03E+00	0.74	0.308	6.70E-16	Myeloid_c0-FRMD4A
OTX2	6.49E-18	2.08E+00	0.37	0.096	1.76E-15	Myeloid_c0-FRMD4A
POU2F2	1.37E-17	1.27E+00	0.75	0.385	3.72E-15	Myeloid_c0-FRMD4A
ZNF217	3.47E-17	2.22E+00	0.54	0.169	9.41E-15	Myeloid_c0-FRMD4A
XBP1	5.93E-17	1.49E+00	0.35	0.108	1.61E-14	Myeloid_c0-FRMD4A
NEUROD1	2.57E-16	1.42E+00	0.49	0.205	6.97E-14	Myeloid_c0-FRMD4A
RUNX1	3.24E-16	1.32E+00	0.57	0.25	8.77E-14	Myeloid_c0-FRMD4A
BCL11A	3.57E-16	1.04E+00	0.36	0.136	9.68E-14	Myeloid_c0-FRMD4A
ESR2	6.04E-16	1.18E+00	0.59	0.219	1.64E-13	Myeloid_c0-FRMD4A
POU2F1	2.16E-15	1.25E+00	0.58	0.202	5.84E-13	Myeloid_c0-FRMD4A

Transcription factor	p_val	avg_log2FC	pct.1	pct.2	p_val_adj	cluster
ETS2	2.82E-15	1.37E+00	0.39	0.125	7.65E-13	Myeloid_c0-FRMD4A
ARID3A	3.44E-15	1.33E+00	0.36	0.148	9.32E-13	Myeloid_c0-FRMD4A
CREM	4.39E-15	1.05E+00	0.25	0.093	1.19E-12	Myeloid_c0-FRMD4A
NFATC1	4.93E-14	4.07E-01	0.28	0.12	1.34E-11	Myeloid_c0-FRMD4A
NFKB1	1.02E-12	1.37E+00	0.58	0.242	2.78E-10	Myeloid_c0-FRMD4A
PROX1	1.04E-12	1.08E+00	0.28	0.121	2.81E-10	Myeloid_c0-FRMD4A
TAL1	4.57E-12	1.69E+00	0.41	0.15	1.24E-09	Myeloid_c0-FRMD4A
AHR	1.24E-11	6.82E-01	0.27	0.103	3.37E-09	Myeloid_c0-FRMD4A
USF2	2.22E-11	1.38E+00	0.34	0.138	6.01E-09	Myeloid_c0-FRMD4A
TWIST1	9.37E-11	1.15E+00	0.47	0.193	2.54E-08	Myeloid_c0-FRMD4A
CREB1	1.07E-10	1.16E+00	0.39	0.176	2.90E-08	Myeloid_c0-FRMD4A
NR1H2	1.13E-10	1.24E+00	0.43	0.181	3.05E-08	Myeloid_c0-FRMD4A
FOXL2	1.15E-10	1.73E+00	0.7	0.445	3.12E-08	Myeloid_c0-FRMD4A
GFI1B	2.52E-10	1.86E+00	0.27	0.097	6.84E-08	Myeloid_c0-FRMD4A
RELA	2.67E-10	8.86E-01	0.6	0.286	7.25E-08	Myeloid_c0-FRMD4A
NCOA1	3.02E-10	2.77E+00	0.28	0.098	8.19E-08	Myeloid_c0-FRMD4A
FLI1	9.47E-10	1.06E+00	0.74	0.434	2.57E-07	Myeloid_c0-FRMD4A
KDM5B	1.52E-09	1.95E+00	0.25	0.12	4.12E-07	Myeloid_c0-FRMD4A
SMAD1	2.95E-09	9.17E-01	0.26	0.115	7.99E-07	Myeloid_c0-FRMD4A
CREB3	4.16E-09	1.08E+00	0.29	0.146	1.13E-06	Myeloid_c0-FRMD4A
FOXO1	7.84E-09	1.38E+00	0.31	0.108	2.13E-06	Myeloid_c0-FRMD4A
SP2	8.81E-09	1.11E+00	0.28	0.105	2.39E-06	Myeloid_c0-FRMD4A
NR1H3	3.99E-08	1.15E+00	0.31	0.152	1.08E-05	Myeloid_c0-FRMD4A
ATF1	1.10E-07	3.05E-01	0.62	0.388	2.97E-05	Myeloid_c0-FRMD4A
CREB3L1	1.36E-07	8.23E-01	0.29	0.144	3.70E-05	Myeloid_c0-FRMD4A
EPAS1	4.01E-07	8.37E-01	0.47	0.194	1.09E-04	Myeloid_c0-FRMD4A
POU4F2	1.09E-06	1.99E+00	0.26	0.103	2.96E-04	Myeloid_c0-FRMD4A
ELK1	1.96E-06	3.41E-01	0.28	0.167	5.32E-04	Myeloid_c0-FRMD4A
SOX10	3.37E-06	3.07E-01	0.31	0.155	9.14E-04	Myeloid_c0-FRMD4A
GATA1	1.41E-05	5.35E-01	0.73	0.586	3.81E-03	Myeloid_c0-FRMD4A
ZEB1	3.35E-05	4.93E-01	0.74	0.507	9.07E-03	Myeloid_c0-FRMD4A
CDX2	7.20E-05	2.85E-01	0.97	0.924	1.95E-02	Myeloid_c0-FRMD4A
MBD2	9.11E-05	4.45E-01	0.57	0.405	2.47E-02	Myeloid_c0-FRMD4A
NFE2L2	1.80E-04	4.54E-01	0.3	0.181	4.87E-02	Myeloid_c0-FRMD4A
ATF4	4.57E-04	1.09E+00	0.37	0.232	1.24E-01	Myeloid_c0-FRMD4A
ATF2	5.74E-03	6.69E-01	0.34	0.226	1.00E+00	Myeloid_c0-FRMD4A
ATF6	8.04E-03	6.93E-01	0.37	0.259	1.00E+00	Myeloid_c0-FRMD4A
RELA	1.49E-30	1.88E+00	0.86	0.264	4.03E-28	Myeloid_c1-EREG
FOS	2.48E-26	1.69E+00	0.36	0.097	6.72E-24	Myeloid_c1-EREG
CEBPB	3.28E-24	1.85E+00	0.83	0.349	8.90E-22	Myeloid_c1-EREG
NFKB1	3.00E-23	8.70E-01	0.67	0.234	8.13E-21	Myeloid_c1-EREG
RUNX1	2.20E-22	1.39E+00	0.67	0.242	5.96E-20	Myeloid_c1-EREG
IKZF1	2.33E-22	2.82E-01	0.36	0.144	6.31E-20	Myeloid_c1-EREG

Transcription factor	p_val	avg_log2FC	pct.1	pct.2	p_val_adj	cluster
ELF3	6.43E-22	1.43E+00	0.28	0.073	1.74E-19	Myeloid_c1-EREG
FOXL2	1.45E-20	1.27E+00	0.89	0.429	3.94E-18	Myeloid_c1-EREG
ARID3A	2.20E-20	1.76E+00	0.36	0.148	5.95E-18	Myeloid_c1-EREG
REL	8.68E-19	1.74E+00	0.85	0.472	2.35E-16	Myeloid_c1-EREG
ZNF217	2.08E-18	1.46E+00	0.47	0.175	5.64E-16	Myeloid_c1-EREG
RARA	9.42E-18	1.14E+00	0.67	0.274	2.55E-15	Myeloid_c1-EREG
RELB	1.05E-17	1.02E+00	0.56	0.242	2.86E-15	Myeloid_c1-EREG
PPARG	1.88E-17	7.00E-01	0.69	0.312	5.09E-15	Myeloid_c1-EREG
BACH1	2.43E-17	1.41E+00	0.27	0.069	6.58E-15	Myeloid_c1-EREG
JUN	4.48E-17	1.56E+00	0.32	0.098	1.22E-14	Myeloid_c1-EREG
ATF1	2.54E-16	1.08E+00	0.78	0.375	6.89E-14	Myeloid_c1-EREG
WT1	7.18E-16	9.63E-01	0.6	0.278	1.95E-13	Myeloid_c1-EREG
EPAS1	5.18E-15	1.36E+00	0.53	0.189	1.40E-12	Myeloid_c1-EREG
CEBPD	7.03E-15	1.30E+00	0.62	0.231	1.90E-12	Myeloid_c1-EREG
KDM5B	1.56E-14	1.52E+00	0.34	0.112	4.24E-12	Myeloid_c1-EREG
BCL11A	3.55E-14	8.22E-01	0.29	0.142	9.63E-12	Myeloid_c1-EREG
POU2F2	4.31E-14	7.21E-01	0.74	0.386	1.17E-11	Myeloid_c1-EREG
NR1H2	7.60E-14	9.21E-01	0.47	0.178	2.06E-11	Myeloid_c1-EREG
NEUROD1	6.70E-13	1.05E+00	0.51	0.203	1.82E-10	Myeloid_c1-EREG
PROX1	1.91E-10	1.07E+00	0.29	0.12	5.17E-08	Myeloid_c1-EREG
USF2	2.68E-10	4.50E-01	0.3	0.142	7.26E-08	Myeloid_c1-EREG
TCF7L2	1.25E-09	5.38E-01	0.41	0.218	3.40E-07	Myeloid_c1-EREG
SOX9	1.70E-09	4.24E-01	0.97	0.842	4.60E-07	Myeloid_c1-EREG
E2F3	4.13E-08	4.31E-01	0.45	0.249	1.12E-05	Myeloid_c1-EREG
JUND	4.26E-08	1.27E+00	0.39	0.175	1.15E-05	Myeloid_c1-EREG
ATF4	8.23E-08	3.06E-01	0.38	0.231	2.23E-05	Myeloid_c1-EREG
SP3	4.48E-07	2.89E-01	0.42	0.262	1.21E-04	Myeloid_c1-EREG
ETV4	6.20E-07	5.87E-01	0.48	0.299	1.68E-04	Myeloid_c1-EREG
TP63	6.13E-06	6.99E-01	0.4	0.25	1.66E-03	Myeloid_c1-EREG
GATA4	6.13E-06	4.65E-01	0.98	0.856	1.66E-03	Myeloid_c1-EREG
VDR	1.94E-05	5.07E-01	0.69	0.488	5.25E-03	Myeloid_c1-EREG
FOSL2	2.05E-05	5.12E-01	0.36	0.248	5.55E-03	Myeloid_c1-EREG
GLI2	4.16E-05	4.29E-01	0.63	0.468	1.13E-02	Myeloid_c1-EREG
GATA2	1.28E-04	6.43E-01	0.51	0.368	3.48E-02	Myeloid_c1-EREG
KLF4	1.81E-04	5.28E-01	0.7	0.565	4.92E-02	Myeloid_c1-EREG
NFE2L2	3.03E-04	7.16E-01	0.26	0.184	8.21E-02	Myeloid_c1-EREG
POU2F1	3.29E-04	4.48E-01	0.32	0.223	8.91E-02	Myeloid_c1-EREG
PPARG	8.05E-15	2.33E+00	0.66	0.314	2.18E-12	Myeloid_c2-SPP1
FOXP1	7.92E-07	2.13E+00	1	0.944	2.15E-04	Myeloid_c2-SPP1
ERG	3.43E-08	1.08E+00	0.82	0.601	9.29E-06	Myeloid_c2-SPP1
NR1H3	3.62E-05	1.05E+00	0.27	0.156	9.80E-03	Myeloid_c2-SPP1
FOXL2	2.66E-08	9.53E-01	0.75	0.441	7.22E-06	Myeloid_c2-SPP1
ZEB2	1.50E-04	9.26E-01	0.99	0.91	4.07E-02	Myeloid_c2-SPP1

Transcription factor	p_val	avg_log2FC	pct.1	pct.2	p_val_adj	cluster
NEUROD1	2.94E-05	9.23E-01	0.39	0.213	7.96E-03	Myeloid_c2-SPP1
RUNX1	2.30E-11	8.35E-01	0.52	0.254	6.22E-09	Myeloid_c2-SPP1
NR1H2	4.31E-05	8.32E-01	0.34	0.188	1.17E-02	Myeloid_c2-SPP1
USF2	1.47E-09	7.54E-01	0.25	0.146	3.99E-07	Myeloid_c2-SPP1
BHLHE22	9.75E-06	7.30E-01	1	0.962	2.64E-03	Myeloid_c2-SPP1
POU2F2	1.05E-11	4.98E-01	0.71	0.388	2.84E-09	Myeloid_c2-SPP1
EPAS1	2.02E-04	4.83E-01	0.35	0.204	5.47E-02	Myeloid_c2-SPP1
HMBOX1	5.08E-05	4.57E-01	0.97	0.874	1.38E-02	Myeloid_c2-SPP1
RFX5	3.08E-32	1.41E+00	0.96	0.358	8.35E-30	Myeloid_c3-FCER1A
BATF	1.79E-30	3.20E+00	0.5	0.109	4.84E-28	Myeloid_c3-FCER1A
IKZF1	3.50E-29	2.54E+00	0.53	0.13	9.48E-27	Myeloid_c3-FCER1A
CREM	8.93E-29	3.39E+00	0.42	0.079	2.42E-26	Myeloid_c3-FCER1A
CREB1	1.42E-28	1.98E+00	0.62	0.157	3.84E-26	Myeloid_c3-FCER1A
SPI1	7.66E-28	7.89E-01	0.52	0.148	2.08E-25	Myeloid_c3-FCER1A
NFKB1	1.24E-27	3.20E+00	0.76	0.227	3.36E-25	Myeloid_c3-FCER1A
SPIB	6.83E-27	8.42E-01	0.31	0.074	1.85E-24	Myeloid_c3-FCER1A
RBPJ	8.52E-26	4.68E+00	0.4	0.103	2.31E-23	Myeloid_c3-FCER1A
ATF1	1.70E-25	2.21E+00	0.81	0.372	4.61E-23	Myeloid_c3-FCER1A
NFATC1	2.93E-25	3.02E+00	0.48	0.103	7.95E-23	Myeloid_c3-FCER1A
RELA	4.25E-25	2.30E+00	0.77	0.272	1.15E-22	Myeloid_c3-FCER1A
IRF4	8.79E-24	1.71E+00	0.33	0.076	2.38E-21	Myeloid_c3-FCER1A
TCF7	1.99E-23	2.26E+00	0.26	0.046	5.38E-21	Myeloid_c3-FCER1A
BCL11A	2.23E-22	1.52E+00	0.41	0.132	6.04E-20	Myeloid_c3-FCER1A
ARID3A	3.62E-21	2.24E+00	0.51	0.135	9.81E-19	Myeloid_c3-FCER1A
RUNX1	1.50E-20	1.59E+00	0.61	0.247	4.06E-18	Myeloid_c3-FCER1A
PAX5	3.68E-19	1.41E+00	0.26	0.078	9.97E-17	Myeloid_c3-FCER1A
RELB	6.41E-19	2.70E+00	0.65	0.234	1.74E-16	Myeloid_c3-FCER1A
FOS	1.21E-17	1.94E+00	0.38	0.095	3.28E-15	Myeloid_c3-FCER1A
NEUROD1	3.11E-17	1.38E+00	0.52	0.202	8.42E-15	Myeloid_c3-FCER1A
EPAS1	1.97E-16	2.06E+00	0.58	0.185	5.35E-14	Myeloid_c3-FCER1A
ZNF217	6.60E-16	1.31E+00	0.5	0.172	1.79E-13	Myeloid_c3-FCER1A
POU2F2	6.80E-15	6.74E-01	0.81	0.38	1.84E-12	Myeloid_c3-FCER1A
WT1	1.97E-14	1.01E+00	0.61	0.277	5.34E-12	Myeloid_c3-FCER1A
E2F3	3.55E-14	1.48E+00	0.55	0.241	9.63E-12	Myeloid_c3-FCER1A
TCF3	2.65E-12	1.21E+00	0.33	0.124	7.17E-10	Myeloid_c3-FCER1A
ZBED1	2.11E-11	1.72E+00	0.34	0.098	5.72E-09	Myeloid_c3-FCER1A
SREBF2	4.58E-11	1.87E+00	0.43	0.168	1.24E-08	Myeloid_c3-FCER1A
NFYA	1.51E-10	9.25E-01	0.38	0.198	4.10E-08	Myeloid_c3-FCER1A
IRF1	1.43E-09	1.37E+00	0.31	0.116	3.87E-07	Myeloid_c3-FCER1A
CREB3	1.50E-09	6.67E-01	0.26	0.148	4.05E-07	Myeloid_c3-FCER1A
XBP1	7.14E-09	8.04E-01	0.25	0.117	1.93E-06	Myeloid_c3-FCER1A
USF2	8.89E-09	1.12E+00	0.26	0.145	2.41E-06	Myeloid_c3-FCER1A
ATF4	2.85E-08	4.35E-01	0.42	0.228	7.73E-06	Myeloid_c3-FCER1A

Transcription factor	p_val	avg_log2FC	pct.1	pct.2	p_val_adj	cluster
PGR	1.27E-07	8.73E-01	0.36	0.19	3.43E-05	Myeloid_c3-FCER1A
PAX8	1.46E-07	4.18E-01	0.98	0.883	3.96E-05	Myeloid_c3-FCER1A
POU2F1	5.44E-07	5.71E-01	0.33	0.222	1.47E-04	Myeloid_c3-FCER1A
CEBPB	7.30E-07	4.44E-01	0.61	0.368	1.98E-04	Myeloid_c3-FCER1A
PROX1	1.24E-06	8.22E-01	0.28	0.121	3.37E-04	Myeloid_c3-FCER1A
TCF7L2	5.23E-06	4.83E-01	0.33	0.225	1.42E-03	Myeloid_c3-FCER1A
REL	8.96E-06	4.34E-01	0.7	0.484	2.43E-03	Myeloid_c3-FCER1A
EOMES	9.49E-06	8.86E-01	0.29	0.163	2.57E-03	Myeloid_c3-FCER1A
TP63	1.28E-05	5.73E-01	0.42	0.248	3.46E-03	Myeloid_c3-FCER1A
TAL1	1.29E-05	9.52E-01	0.25	0.163	3.49E-03	Myeloid_c3-FCER1A
VDR	2.66E-05	3.16E-01	0.7	0.488	7.20E-03	Myeloid_c3-FCER1A
CEBPD	2.75E-05	8.47E-01	0.48	0.242	7.45E-03	Myeloid_c3-FCER1A
JUND	5.10E-05	7.38E-01	0.35	0.178	1.38E-02	Myeloid_c3-FCER1A
NFE2L2	2.87E-04	8.34E-01	0.33	0.178	7.78E-02	Myeloid_c3-FCER1A
GATA1	5.29E-04	3.85E-01	0.71	0.588	1.43E-01	Myeloid_c3-FCER1A
NR1H2	1.41E-03	6.01E-01	0.25	0.196	3.82E-01	Myeloid_c3-FCER1A
E2F2	2.05E-03	7.89E-01	0.58	0.45	5.55E-01	Myeloid_c3-FCER1A
FOSL2	2.11E-03	4.31E-01	0.32	0.251	5.72E-01	Myeloid_c3-FCER1A
TBX21	2.76E-30	4.61E+00	0.37	0.025	7.48E-28	Myeloid_c4-CD69
EOMES	5.16E-18	1.83E+00	0.49	0.147	1.40E-15	Myeloid_c4-CD69
BATF	4.77E-14	1.07E+00	0.27	0.128	1.29E-11	Myeloid_c4-CD69
RELB	6.68E-14	8.80E-01	0.57	0.241	1.81E-11	Myeloid_c4-CD69
STAT4	1.70E-13	1.28E+00	0.48	0.218	4.62E-11	Myeloid_c4-CD69
TCF7L2	6.20E-11	1.18E+00	0.54	0.208	1.68E-08	Myeloid_c4-CD69
RUNX3	8.49E-10	1.68E+00	0.65	0.403	2.30E-07	Myeloid_c4-CD69
BCL11A	1.07E-09	1.65E+00	0.28	0.142	2.89E-07	Myeloid_c4-CD69
WT1	1.40E-08	1.06E+00	0.5	0.286	3.79E-06	Myeloid_c4-CD69
REL	6.21E-07	4.25E-01	0.71	0.483	1.68E-04	Myeloid_c4-CD69
USF2	8.29E-06	4.88E-01	0.27	0.144	2.25E-03	Myeloid_c4-CD69
VDR	2.98E-05	9.61E-01	0.64	0.492	8.08E-03	Myeloid_c4-CD69
PAX8	6.36E-05	5.83E-01	0.93	0.887	1.72E-02	Myeloid_c4-CD69
RUNX1	7.07E-05	4.54E-01	0.31	0.272	1.92E-02	Myeloid_c4-CD69
STAT6	1.09E-04	8.85E-01	0.6	0.446	2.95E-02	Myeloid_c4-CD69
ZNF217	1.12E-03	4.10E-01	0.27	0.192	3.03E-01	Myeloid_c4-CD69
TAL1	1.79E-03	4.71E-01	0.25	0.163	4.86E-01	Myeloid_c4-CD69
RELA	2.67E-03	1.27E+00	0.33	0.308	7.24E-01	Myeloid_c4-CD69
ZEB1	3.39E-03	4.97E-01	0.65	0.515	9.18E-01	Myeloid_c4-CD69
NFKB1	4.06E-03	7.10E-01	0.23	0.271	1.00E+00	Myeloid_c4-CD69
TFDP1	5.20E-03	5.32E-01	0.39	0.298	1.00E+00	Myeloid_c4-CD69
NFYA	6.86E-03	3.11E-01	0.26	0.208	1.00E+00	Myeloid_c4-CD69
NR5A1	8.53E-44	3.67E-01	1	0.999	2.31E-41	Myeloid_c5-S100A8
FOXP2	2.09E-40	2.76E+00	1	0.863	5.67E-38	Myeloid_c5-S100A8
THAP11	2.62E-35	2.06E+00	1	0.973	7.09E-33	Myeloid_c5-S100A8

Transcription factor	p_val	avg_log2FC	pct.1	pct.2	p_val_adj	cluster
ZNF639	6.63E-31	2.01E+00	0.99	0.882	1.80E-28	Myeloid_c5-S100A8
MXI1	8.76E-29	2.27E+00	1	0.963	2.37E-26	Myeloid_c5-S100A8
E2F6	3.61E-24	1.68E+00	0.99	0.956	9.78E-22	Myeloid_c5-S100A8
FOXP1	4.71E-24	1.28E+00	1	0.944	1.28E-21	Myeloid_c5-S100A8
NR2F2	7.59E-24	2.80E-01	1	0.914	2.06E-21	Myeloid_c5-S100A8
ATF3	1.09E-20	1.59E+00	0.99	0.873	2.96E-18	Myeloid_c5-S100A8
HMBOX1	2.65E-20	1.53E+00	1	0.872	7.18E-18	Myeloid_c5-S100A8
CEBPB	1.21E-19	8.41E-01	0.83	0.349	3.29E-17	Myeloid_c5-S100A8
TGIF2	6.87E-19	1.15E+00	0.99	0.886	1.86E-16	Myeloid_c5-S100A8
HNF1A	3.85E-17	3.26E-01	1	0.987	1.04E-14	Myeloid_c5-S100A8
NR2F6	8.34E-17	8.51E-01	1	0.909	2.26E-14	Myeloid_c5-S100A8
ZEB2	1.19E-14	8.96E-01	0.99	0.91	3.24E-12	Myeloid_c5-S100A8
KLF13	1.36E-13	1.06E+00	1	0.926	3.68E-11	Myeloid_c5-S100A8
HBP1	2.15E-13	5.30E-01	1	0.928	5.83E-11	Myeloid_c5-S100A8
ZBTB7A	5.03E-13	6.21E-01	1	0.968	1.36E-10	Myeloid_c5-S100A8
FOSL2	1.77E-11	7.74E-01	0.5	0.236	4.79E-09	Myeloid_c5-S100A8
ZNF263	2.47E-10	4.66E-01	1	0.993	6.68E-08	Myeloid_c5-S100A8
GLI2	2.59E-10	2.53E-01	0.73	0.459	7.02E-08	Myeloid_c5-S100A8
TCF7L2	1.00E-09	5.81E-01	0.37	0.222	2.72E-07	Myeloid_c5-S100A8
ZEB1	1.13E-09	4.84E-01	0.88	0.496	3.07E-07	Myeloid_c5-S100A8
MBD1	1.30E-09	5.62E-01	1	0.979	3.51E-07	Myeloid_c5-S100A8
NFATC2	3.02E-09	3.14E-01	0.95	0.757	8.18E-07	Myeloid_c5-S100A8
HHEX	2.68E-07	4.55E-01	1	0.857	7.25E-05	Myeloid_c5-S100A8
SNAI2	3.19E-06	3.77E-01	0.99	0.96	8.65E-04	Myeloid_c5-S100A8
BHLHE22	6.17E-06	4.71E-01	0.98	0.963	1.67E-03	Myeloid_c5-S100A8
ATF4	5.59E-04	5.86E-01	0.3	0.238	1.52E-01	Myeloid_c5-S100A8
ESR2	1.08E-15	1.91E+00	0.58	0.22	2.93E-13	Myeloid_c6-MGP
SPI1	1.42E-14	1.66E+00	0.44	0.154	3.84E-12	Myeloid_c6-MGP
FOXL2	1.72E-12	1.37E+00	0.75	0.441	4.65E-10	Myeloid_c6-MGP
ETS2	6.52E-10	1.09E+00	0.31	0.132	1.77E-07	Myeloid_c6-MGP
POU2F1	6.67E-10	6.27E-01	0.38	0.218	1.81E-07	Myeloid_c6-MGP
RUNX1	7.37E-10	4.89E-01	0.48	0.258	2.00E-07	Myeloid_c6-MGP
ELK1	3.50E-09	1.82E+00	0.42	0.155	9.48E-07	Myeloid_c6-MGP
USF2	1.73E-08	9.76E-01	0.25	0.146	4.68E-06	Myeloid_c6-MGP
CDX2	7.47E-08	4.75E-01	0.99	0.922	2.02E-05	Myeloid_c6-MGP
IKZF1	2.34E-07	6.79E-01	0.27	0.152	6.34E-05	Myeloid_c6-MGP
TWIST1	5.18E-07	8.55E-01	0.41	0.198	1.40E-04	Myeloid_c6-MGP
ATF2	6.18E-07	7.10E-01	0.39	0.222	1.67E-04	Myeloid_c6-MGP
POU2F2	3.34E-06	5.24E-01	0.57	0.4	9.04E-04	Myeloid_c6-MGP
PPARG	4.86E-06	2.83E-01	0.48	0.329	1.32E-03	Myeloid_c6-MGP
FLI1	1.17E-05	4.89E-01	0.64	0.442	3.18E-03	Myeloid_c6-MGP
CREB1	3.44E-05	8.12E-01	0.25	0.188	9.34E-03	Myeloid_c6-MGP
ATF4	5.34E-05	9.63E-01	0.43	0.227	1.45E-02	Myeloid_c6-MGP

Transcription factor	p_val	avg_log2FC	pct.1	pct.2	p_val_adj	cluster
REL	5.87E-05	2.75E-01	0.66	0.488	1.59E-02	Myeloid_c6-MGP
KLF4	7.24E-05	7.31E-01	0.73	0.562	1.96E-02	Myeloid_c6-MGP
CEBPB	9.85E-05	3.91E-01	0.54	0.373	2.67E-02	Myeloid_c6-MGP
TAL1	1.30E-04	6.33E-01	0.33	0.157	3.52E-02	Myeloid_c6-MGP
GATA1	1.89E-04	4.23E-01	0.75	0.584	5.13E-02	Myeloid_c6-MGP
SP3	2.41E-04	5.16E-01	0.4	0.263	6.52E-02	Myeloid_c6-MGP
MBD2	4.01E-04	3.61E-01	0.57	0.405	1.09E-01	Myeloid_c6-MGP
NFATC1	5.52E-04	7.70E-01	0.28	0.12	1.50E-01	Myeloid_c6-MGP
NEUROD1	2.21E-03	5.83E-01	0.35	0.217	6.00E-01	Myeloid_c6-MGP
STAT6	3.09E-03	4.94E-01	0.57	0.448	8.37E-01	Myeloid_c6-MGP
RARA	3.40E-03	2.93E-01	0.45	0.292	9.20E-01	Myeloid_c6-MGP
EOMES	8.41E-03	3.38E-01	0.27	0.165	1.00E+00	Myeloid_c6-MGP
RFX5	1.07E-52	4.30E+00	0.99	0.355	2.91E-50	Myeloid_c7-CPVL
SPIB	1.01E-31	3.78E+00	0.36	0.07	2.73E-29	Myeloid_c7-CPVL
POU2F2	6.41E-27	1.85E+00	0.88	0.374	1.74E-24	Myeloid_c7-CPVL
SREBF2	2.64E-26	1.88E+00	0.62	0.152	7.15E-24	Myeloid_c7-CPVL
NFYA	1.80E-25	2.34E+00	0.63	0.177	4.87E-23	Myeloid_c7-CPVL
MYC	3.10E-21	2.21E+00	0.37	0.058	8.39E-19	Myeloid_c7-CPVL
NFYB	3.66E-21	3.71E+00	0.36	0.031	9.92E-19	Myeloid_c7-CPVL
SREBF1	8.66E-20	1.31E+00	0.3	0.085	2.35E-17	Myeloid_c7-CPVL
BCL11A	5.62E-19	1.20E+00	0.36	0.136	1.52E-16	Myeloid_c7-CPVL
ZBED1	1.14E-18	1.68E+00	0.34	0.098	3.08E-16	Myeloid_c7-CPVL
TCF3	7.45E-18	2.50E+00	0.49	0.111	2.02E-15	Myeloid_c7-CPVL
PAX5	1.06E-17	2.91E+00	0.34	0.072	2.87E-15	Myeloid_c7-CPVL
CREB3	1.49E-17	1.62E+00	0.44	0.133	4.05E-15	Myeloid_c7-CPVL
CREB1	2.84E-14	1.12E+00	0.37	0.178	7.69E-12	Myeloid_c7-CPVL
POU2F1	1.73E-10	5.92E-01	0.39	0.218	4.69E-08	Myeloid_c7-CPVL
E2F3	3.93E-09	1.14E+00	0.5	0.245	1.06E-06	Myeloid_c7-CPVL
E2F2	2.00E-08	1.10E+00	0.66	0.443	5.41E-06	Myeloid_c7-CPVL
ATF6	9.98E-07	1.28E+00	0.48	0.25	2.70E-04	Myeloid_c7-CPVL
VDR	2.02E-06	7.13E-01	0.71	0.487	5.46E-04	Myeloid_c7-CPVL
NFE2L2	2.13E-06	1.31E+00	0.32	0.179	5.78E-04	Myeloid_c7-CPVL
RUNX3	4.12E-05	3.46E-01	0.61	0.407	1.12E-02	Myeloid_c7-CPVL
MBD2	5.28E-05	6.60E-01	0.58	0.404	1.43E-02	Myeloid_c7-CPVL
IRF1	5.48E-05	5.61E-01	0.29	0.117	1.48E-02	Myeloid_c7-CPVL
FLI1	1.10E-04	2.90E-01	0.63	0.443	2.98E-02	Myeloid_c7-CPVL
EOMES	1.65E-04	3.37E-01	0.28	0.164	4.48E-02	Myeloid_c7-CPVL
WT1	3.14E-03	4.00E-01	0.38	0.296	8.52E-01	Myeloid_c7-CPVL
TP63	4.25E-03	6.65E-01	0.31	0.258	1.00E+00	Myeloid_c7-CPVL

Supplementary Table 2. DEGs in Myeloid_c2-SPP1 (TAA vs. Control) with NF-κB and EMT pathway enrichment

Gene	p_val	avg_log2FC	pct.1	pct.2	p_val_adj	TNF-α[a]	EMT[b]	shared[c]
PMEPA1	5.03E-50	3.19E-01	0.212	0.065	1.54E-45	Y	Y	Y
IL6	2.19E-35	5.43E-01	0.136	0.035	6.72E-31	Y	Y	Y
GADD45A	1.09E-34	2.62E-01	0.262	0.126	3.34E-30	Y	Y	Y
PTX3	2.09E-24	3.32E-01	0.147	0.059	6.42E-20	Y	Y	Y
GADD45B	5.26E-11	2.63E-01	0.746	0.621	1.61E-06	Y	Y	Y
FN1	0	2.58E+00	0.759	0.229	0		Y	Y
VIM	1.80E-209	9.23E-01	0.981	0.949	5.52E-205		Y	Y
VCAN	5.83E-191	1.27E+00	0.549	0.156	1.79E-186		Y	Y
TIMP1	4.71E-188	1.78E+00	0.865	0.625	1.44E-183		Y	Y
DCN	5.32E-135	-4.02E-01	0.003	0.106	1.63E-130		Y	Y
FLNA	1.01E-125	7.85E-01	0.692	0.401	3.11E-121		Y	Y
NT5E	6.73E-88	4.99E-01	0.196	0.012	2.06E-83		Y	Y
BASP1	8.90E-84	8.12E-01	0.522	0.275	2.73E-79		Y	Y
LGALS1	2.32E-67	4.51E-01	0.932	0.851	7.10E-63		Y	Y
TGFBI	6.76E-67	6.52E-01	0.782	0.628	2.07E-62		Y	Y
TPM4	2.17E-65	4.45E-01	0.796	0.584	6.67E-61		Y	Y
CXCL12	1.22E-54	-4.37E-01	0.022	0.1	3.73E-50		Y	Y
TGFB1	1.32E-54	2.84E-01	0.745	0.495	4.06E-50		Y	Y
HTRA1	2.37E-48	3.11E-01	0.384	0.191	7.26E-44		Y	Y
MGP	1.95E-43	-5.69E-01	0.067	0.168	5.97E-39		Y	Y
DAB2	2.25E-10	-5.66E-01	0.598	0.593	6.92E-06		Y	Y
CXCL1	4.34E-199	2.19E+00	0.51	0.125	1.33E-194	Y	Y	
CD44	6.30E-145	8.32E-01	0.892	0.738	1.93E-140	Y	Y	
VEGFA	1.98E-78	5.32E-01	0.545	0.279	6.08E-74	Y	Y	
INHBA	1.50E-22	3.47E-01	0.127	0.049	4.59E-18	Y	Y	
AREG	3.68E-16	4.47E-01	0.326	0.223	1.13E-11	Y	Y	
GEM	4.46E-11	2.67E-01	0.207	0.138	1.37E-06	Y	Y	
ANPEP	8.23E-234	1.25E+00	0.665	0.238	2.52E-229		Y	
SPP1	2.98E-110	2.12E+00	0.603	0.301	9.14E-106		Y	
CAPG	3.72E-100	3.72E-01	0.823	0.504	1.14E-95		Y	
ITGA5	2.75E-93	6.01E-01	0.578	0.302	8.45E-89		Y	
CXCL8	1.57E-91	6.05E-01	0.876	0.698	4.81E-87		Y	
SNTB1	6.78E-75	4.75E-01	0.364	0.141	2.08E-70		Y	
EMP3	2.30E-57	4.22E-01	0.867	0.735	7.04E-53		Y	
THBS1	4.03E-39	7.12E-01	0.266	0.12	1.24E-34		Y	
GLIPR1	7.09E-16	-3.84E-01	0.534	0.553	2.17E-11		Y	
FUCA1	6.28E-12	-4.88E-01	0.177	0.232	1.93E-07		Y	

Gene	p_val	avg_log2FC	pct.1	pct.2	p_val_adj	TNF-α[a]	EMT[b]	shared[c]
PHLDA1	9.31E-241	1.83E+00	0.755	0.45	2.86E-236	Y		Y
CXCL3	3.54E-193	1.28E+00	0.843	0.579	1.09E-188	Y		Y
NFKBIA	8.49E-189	1.01E+00	0.905	0.78	2.61E-184	Y		Y
CXCL2	1.64E-170	1.24E+00	0.859	0.633	5.03E-166	Y		Y
G0S2	1.17E-162	1.96E+00	0.582	0.241	3.58E-158	Y		Y
BTG1	5.32E-149	8.81E-01	0.869	0.663	1.63E-144	Y		Y
SOCS3	3.81E-133	8.69E-01	0.726	0.428	1.17E-128	Y		Y
PNRC1	2.07E-112	5.95E-01	0.922	0.798	6.36E-108	Y		Y
IER3	1.18E-104	7.42E-01	0.875	0.715	3.62E-100	Y		Y
IFNGR2	2.27E-86	5.24E-01	0.772	0.541	6.96E-82	Y		Y
ACKR3	6.22E-85	7.61E-01	0.21	0.023	1.91E-80	Y		Y
MSC	9.64E-82	4.28E-01	0.19	0.014	2.96E-77	Y		Y
SPHK1	2.65E-78	4.64E-01	0.452	0.21	8.13E-74	Y		Y
TNFAIP8	6.97E-77	5.74E-01	0.697	0.467	2.14E-72	Y		Y
NINJ1	3.71E-73	5.03E-01	0.811	0.614	1.14E-68	Y		Y
DENND5A	9.56E-70	5.30E-01	0.68	0.444	2.93E-65	Y		Y
DUSP1	5.31E-68	4.63E-01	0.889	0.737	1.63E-63	Y		Y
NFIL3	5.47E-67	3.72E-01	0.457	0.221	1.68E-62	Y		Y
CDKN1A	2.71E-66	4.33E-01	0.784	0.565	8.31E-62	Y		Y
ATP2B1	1.08E-57	4.18E-01	0.765	0.537	3.30E-53	Y		Y
CCL2	3.67E-55	1.01E+00	0.606	0.426	1.13E-50	Y		Y
PPP1R15A	8.02E-52	4.07E-01	0.85	0.659	2.46E-47	Y		Y
MYC	1.81E-44	3.97E-01	0.321	0.158	5.54E-40	Y		Y
TSC22D1	4.56E-42	4.75E-01	0.346	0.181	1.40E-37	Y		Y
BHLHE40	1.05E-39	3.14E-01	0.592	0.388	3.23E-35	Y		Y
JUNB	4.22E-35	4.80E-01	0.794	0.627	1.30E-30	Y		Y
TNFAIP2	2.51E-30	3.73E-01	0.683	0.539	7.69E-26	Y		Y
IRS2	7.43E-09	-3.22E-01	0.232	0.277	0.000227961	Y		Y
CCL20	1.08E-205	2.09E+00	0.626	0.257	3.31E-201	Y		
IL1B	2.32E-173	1.32E+00	0.836	0.56	7.11E-169	Y		
EHD1	1.24E-129	8.88E-01	0.578	0.27	3.80E-125	Y		
CEBPB	3.04E-122	7.48E-01	0.82	0.596	9.31E-118	Y		
DUSP4	1.37E-116	8.56E-01	0.328	0.064	4.20E-112	Y		
CLCF1	5.04E-116	7.15E-01	0.33	0.068	1.55E-111	Y		
TRAF1	4.09E-106	6.43E-01	0.592	0.285	1.25E-101	Y		
TNIP1	1.16E-101	6.00E-01	0.569	0.284	3.57E-97	Y		
DUSP2	2.84E-95	7.60E-01	0.717	0.467	8.70E-91	Y		
MAP3K8	1.21E-91	6.23E-01	0.796	0.586	3.72E-87	Y		
PTGS2	7.57E-89	8.79E-01	0.474	0.218	2.32E-84	Y		

Gene	p_val	avg_log2FC	pct.1	pct.2	p_val_adj	TNF-α[a]	EMT[b]	shared[c]
ZC3H12A	4.73E-86	4.65E-01	0.476	0.213	1.45E-81	Y		
BCL2A1	3.82E-85	7.94E-01	0.823	0.607	1.17E-80	Y		
FOSL2	1.03E-84	4.98E-01	0.631	0.355	3.17E-80	Y		
PHLDA2	4.28E-83	6.70E-01	0.438	0.193	1.31E-78	Y		
SOD2	5.94E-83	6.27E-01	0.901	0.812	1.82E-78	Y		
SERPINB2	3.17E-82	1.32E+00	0.213	0.027	9.74E-78	Y		
TNIP2	1.53E-79	3.53E-01	0.346	0.12	4.70E-75	Y		
MXD1	2.16E-79	5.48E-01	0.578	0.313	6.64E-75	Y		
BCL3	7.05E-71	4.28E-01	0.513	0.26	2.16E-66	Y		
OLR1	2.02E-68	5.11E-01	0.647	0.393	6.19E-64	Y		
TNF	1.31E-67	4.91E-01	0.609	0.358	4.03E-63	Y		
EIF1	1.64E-67	-4.55E-01	0.981	0.966	5.02E-63	Y		
IL7R	9.65E-67	4.70E-01	0.263	0.077	2.96E-62	Y		
IL1A	1.66E-66	6.52E-01	0.385	0.167	5.09E-62	Y		
RNF19B	1.18E-65	3.98E-01	0.472	0.245	3.62E-61	Y		
CCL4	1.92E-64	4.60E-01	0.882	0.737	5.90E-60	Y		
SPSB1	3.81E-61	3.99E-01	0.262	0.082	1.17E-56	Y		
MAP2K3	9.38E-61	4.35E-01	0.611	0.397	2.88E-56	Y		
TRIP10	5.84E-58	3.07E-01	0.29	0.109	1.79E-53	Y		
HBEGF	7.39E-56	4.69E-01	0.411	0.212	2.27E-51	Y		
FOSL1	3.40E-50	2.57E-01	0.332	0.145	1.04E-45	Y		
LIF	2.74E-46	3.62E-01	0.117	0.011	8.40E-42	Y		
PFKFB3	1.04E-43	3.62E-01	0.651	0.447	3.20E-39	Y		
RCAN1	9.65E-43	4.68E-01	0.303	0.147	2.96E-38	Y		
RIPK2	7.68E-42	2.85E-01	0.526	0.323	2.36E-37	Y		
TNFAIP6	1.36E-41	4.95E-01	0.277	0.124	4.16E-37	Y		
DUSP5	3.02E-40	3.03E-01	0.516	0.32	9.27E-36	Y		
ETS2	1.01E-34	2.58E-01	0.72	0.536	3.10E-30	Y		
NAMPT	1.39E-30	2.81E-01	0.863	0.713	4.27E-26	Y		
IL18	7.44E-11	-3.38E-01	0.405	0.435	2.28E-06	Y		
LITAF	4.80E-10	-2.64E-01	0.796	0.739	1.47E-05	Y		
SGK1	1.04E-07	-4.26E-01	0.576	0.57	0.003184888	Y		

[a]DEGs enriched in TNF-α signaling via NF-κB pathway

[b]DEGs enriched in EMT pathway

[c]DEGs enriched in the above two pathways and shared with Fibroblast_c1-POSTN

Supplementary Table 3. DEGs in Fibroblast_c1-POSTN (TAA vs. Control) with NF-κB and EMT pathway enrichment

Gene	p_val	avg_log2FC	pct.1	pct.2	p_val_adj	TNF-α[a]	EMT[b]	shared[c]
GADD45B	2.35E-164	1.19E+00	0.827	0.638	7.20E-160	Y	Y	Y
GADD45A	2.77E-115	5.67E-01	0.579	0.311	8.49E-111	Y	Y	Y
PMEPA1	1.23E-111	5.65E-01	0.662	0.404	3.77E-107	Y	Y	Y
PTX3	1.67E-42	4.60E-01	0.161	0.049	5.13E-38	Y	Y	Y
IL6	8.46E-36	8.02E-01	0.212	0.1	2.59E-31	Y	Y	Y
FN1	0	1.36E+00	0.985	0.892	0		Y	Y
HTRA1	0	9.67E-01	0.918	0.705	0		Y	Y
DCN	0	-2.87E+00	0.859	0.953	0		Y	Y
LGALS1	0	9.10E-01	0.973	0.914	0		Y	Y
VIM	1.30E-291	7.33E-01	0.996	0.989	3.99E-287		Y	Y
CXCL12	6.03E-290	9.35E-01	0.916	0.635	1.85E-285		Y	Y
TPM4	3.66E-239	8.59E-01	0.901	0.699	1.12E-234		Y	Y
BASP1	4.37E-224	6.83E-01	0.61	0.216	1.34E-219		Y	Y
TGFB1	4.71E-194	8.33E-01	0.616	0.291	1.44E-189		Y	Y
VCAN	7.65E-187	7.50E-01	0.968	0.848	2.35E-182		Y	Y
TGFBI	1.02E-120	6.57E-01	0.346	0.089	3.12E-116		Y	Y
TIMP1	1.01E-94	5.43E-01	0.981	0.948	3.09E-90		Y	Y
MGP	3.08E-57	-3.43E-01	0.987	0.984	9.43E-53		Y	Y
FLNA	1.36E-38	3.59E-01	0.743	0.626	4.18E-34		Y	Y
NT5E	2.39E-34	-3.28E-01	0.352	0.47	7.33E-30		Y	Y
DAB2	4.70E-21	-3.15E-01	0.477	0.544	1.44E-16		Y	Y
JUN	7.53E-206	9.31E-01	0.959	0.845	2.31E-201	Y	Y	
TNC	1.07E-177	1.05E+00	0.415	0.085	3.28E-173	Y	Y	
TNFAIP3	2.10E-87	9.49E-01	0.439	0.223	6.44E-83	Y	Y	
RHOB	2.78E-80	6.16E-01	0.778	0.618	8.52E-76	Y	Y	
ID2	3.31E-30	-5.01E-01	0.405	0.504	1.02E-25	Y	Y	
SERPINE1	1.04E-19	3.69E-01	0.459	0.351	3.20E-15	Y	Y	
CCN1	2.45E-10	3.38E-01	0.855	0.782	7.52E-06	Y	Y	
COL3A1	0	2.33E+00	0.983	0.838	0		Y	
ABI3BP	0	-2.04E+00	0.564	0.896	0		Y	
SLIT2	0	-2.33E+00	0.487	0.806	0		Y	
SPARC	0	2.29E+00	0.984	0.892	0		Y	
THBS2	0	1.30E+00	0.917	0.638	0		Y	
COL1A2	0	1.89E+00	0.993	0.957	0		Y	
PCOLCE	0	1.30E+00	0.967	0.842	0		Y	
TPM2	0	1.44E+00	0.928	0.638	0		Y	
COL5A1	0	1.04E+00	0.894	0.603	0		Y	
SERPINH1	0	1.26E+00	0.889	0.579	0		Y	
MFAP5	0	-2.94E+00	0.068	0.743	0		Y	
POSTN	0	3.82E+00	0.868	0.084	0		Y	
COL4A1	0	1.68E+00	0.88	0.461	0		Y	

Gene	p_val	avg_log2FC	pct.1	pct.2	p_val_adj	TNF-α[a]	EMT[b]	shared[c]
COL4A2	0	1.60E+00	0.913	0.505	0		Y	
MMP14	0	1.21E+00	0.75	0.275	0		Y	
PPIB	0	1.10E+00	0.963	0.861	0		Y	
COL1A1	0	2.88E+00	0.981	0.803	0		Y	
MYL9	0	1.18E+00	0.921	0.712	0		Y	
COL6A2	0	9.76E-01	0.984	0.939	0		Y	
TIMP3	0	-2.60E+00	0.425	0.897	0		Y	
FBLN1	0	-1.89E+00	0.917	0.946	0		Y	
BGN	0	2.36E+00	0.985	0.86	0		Y	
COL5A2	6.03E-306	9.95E-01	0.926	0.7	1.85E-301		Y	
LAMA2	2.86E-277	-1.66E+00	0.667	0.844	8.77E-273		Y	
IGFBP2	1.30E-270	1.23E+00	0.596	0.153	4.00E-266		Y	
SERPINE2	1.56E-262	-1.91E+00	0.223	0.553	4.78E-258		Y	
FBLN2	9.18E-259	-1.59E+00	0.473	0.769	2.81E-254		Y	
COMP	1.33E-255	-2.14E+00	0.022	0.256	4.07E-251		Y	
LUM	2.46E-244	-1.32E+00	0.788	0.907	7.53E-240		Y	
EFEMP2	2.72E-242	7.38E-01	0.84	0.574	8.34E-238		Y	
PFN2	1.66E-232	7.78E-01	0.679	0.331	5.10E-228		Y	
LOXL2	3.13E-229	7.51E-01	0.452	0.074	9.60E-225		Y	
CTHRC1	3.96E-199	1.05E+00	0.705	0.38	1.21E-194		Y	
PCOLCE2	1.83E-191	-1.66E+00	0.478	0.678	5.60E-187		Y	
TAGLN	2.29E-187	1.10E+00	0.925	0.739	7.01E-183		Y	
P3H1	2.02E-179	6.21E-01	0.545	0.213	6.19E-175		Y	
LRP1	6.28E-170	-9.12E-01	0.897	0.922	1.93E-165		Y	
GPX7	1.25E-163	4.92E-01	0.44	0.129	3.84E-159		Y	
TGM2	9.01E-159	6.87E-01	0.653	0.317	2.76E-154		Y	
PLOD1	5.31E-156	5.44E-01	0.54	0.222	1.63E-151		Y	
MYLK	1.19E-150	5.45E-01	0.416	0.113	3.64E-146		Y	
ELN	8.10E-145	1.62E+00	0.844	0.695	2.48E-140		Y	
GAS1	8.63E-145	-8.33E-01	0.318	0.581	2.65E-140		Y	
COL8A2	1.18E-144	-3.30E-01	0.067	0.272	3.62E-140		Y	
TGFBR3	5.93E-144	-7.87E-01	0.202	0.458	1.82E-139		Y	
PMP22	5.51E-139	-7.01E-01	0.803	0.845	1.69E-134		Y	
DST	3.85E-138	-7.86E-01	0.796	0.827	1.18E-133		Y	
SLIT3	6.28E-132	-1.22E+00	0.396	0.609	1.93E-127		Y	
COL6A3	2.96E-131	5.75E-01	0.931	0.817	9.08E-127		Y	
BMP1	7.08E-131	4.58E-01	0.549	0.262	2.17E-126		Y	
FOXC2	5.38E-125	4.75E-01	0.399	0.133	1.65E-120		Y	
FAP	7.37E-116	4.45E-01	0.706	0.421	2.26E-111		Y	
GPC1	4.22E-114	4.13E-01	0.337	0.097	1.29E-109		Y	
THY1	4.87E-110	4.08E-01	0.662	0.356	1.49E-105		Y	
LOXL1	6.84E-108	4.86E-01	0.662	0.413	2.10E-103		Y	
VCAM1	8.04E-100	5.83E-01	0.295	0.076	2.47E-95		Y	

Gene	p_val	avg_log2FC	pct.1	pct.2	p_val_adj	TNF-α[a]	EMT[b]	shared[c]
COL5A3	6.47E-94	3.84E-01	0.334	0.109	1.99E-89		Y	
EDIL3	9.74E-94	4.00E-01	0.844	0.574	2.99E-89		Y	
SFRP4	2.19E-92	-1.89E+00	0.641	0.721	6.71E-88		Y	
ECM1	8.80E-90	-6.26E-01	0.34	0.546	2.70E-85		Y	
CDH2	1.38E-89	3.66E-01	0.279	0.075	4.24E-85		Y	
SPOCK1	2.58E-85	2.77E-01	0.596	0.348	7.90E-81		Y	
FMOD	6.71E-79	-7.10E-01	0.427	0.593	2.06E-74		Y	
IGFBP3	4.22E-78	9.35E-01	0.316	0.118	1.30E-73		Y	
CD59	8.63E-77	4.27E-01	0.877	0.749	2.65E-72		Y	
RGS4	8.71E-74	4.32E-01	0.366	0.162	2.67E-69		Y	
PDGFRB	4.80E-71	4.14E-01	0.766	0.597	1.47E-66		Y	
ACTA2	6.81E-68	1.12E+00	0.468	0.29	2.09E-63		Y	
CRLF1	8.25E-67	-4.09E-01	0.08	0.214	2.53E-62		Y	
ITGB1	2.18E-60	3.62E-01	0.929	0.841	6.69E-56		Y	
TPM1	4.37E-54	3.43E-01	0.884	0.712	1.34E-49		Y	
SNAI2	6.87E-53	2.95E-01	0.316	0.15	2.11E-48		Y	
PDLIM4	9.59E-48	2.82E-01	0.506	0.35	2.94E-43		Y	
SFRP1	8.42E-46	-7.35E-01	0.252	0.385	2.58E-41		Y	
FGF2	1.22E-42	-2.77E-01	0.165	0.294	3.73E-38		Y	
FBN1	1.05E-25	-6.72E-01	0.881	0.868	3.21E-21		Y	
NOTCH2	6.28E-25	-3.11E-01	0.414	0.506	1.93E-20		Y	
FSTL3	8.83E-24	-3.17E-01	0.272	0.369	2.71E-19		Y	
COL12A1	7.50E-17	-6.31E-01	0.505	0.546	2.30E-12		Y	
FERMT2	7.03E-15	-3.42E-01	0.607	0.614	2.16E-10		Y	
ITGB5	6.45E-14	-3.08E-01	0.677	0.673	1.98E-09		Y	
COPA	2.16E-13	-2.60E-01	0.583	0.597	6.63E-09		Y	
TNFRSF11B	2.02E-11	-2.96E-01	0.078	0.124	6.19E-07		Y	
PRRX1	3.25E-11	-3.24E-01	0.812	0.747	9.96E-07		Y	
NTM	3.42E-09	-3.51E-01	0.15	0.192	0.000104792		Y	
PHLDA1	0	1.75E+00	0.696	0.23	0	Y		Y
PNRC1	2.81E-249	7.76E-01	0.929	0.771	8.62E-245	Y		Y
ACKR3	1.06E-237	-1.22E+00	0.27	0.612	3.26E-233	Y		Y
CCL2	1.52E-222	1.96E+00	0.682	0.335	4.66E-218	Y		Y
SOCS3	3.84E-187	1.08E+00	0.773	0.498	1.18E-182	Y		Y
CDKN1A	5.26E-183	1.25E+00	0.719	0.422	1.61E-178	Y		Y
DUSP1	6.19E-181	1.05E+00	0.941	0.826	1.90E-176	Y		Y
JUNB	4.33E-165	9.32E-01	0.944	0.837	1.33E-160	Y		Y
TSC22D1	6.92E-164	7.36E-01	0.838	0.584	2.12E-159	Y		Y
CXCL3	1.84E-124	1.49E+00	0.376	0.12	5.65E-120	Y		Y
SPHK1	1.89E-117	4.49E-01	0.36	0.107	5.80E-113	Y		Y
PPP1R15A	2.61E-115	7.63E-01	0.827	0.652	8.01E-111	Y		Y
NFKBIA	7.15E-111	8.13E-01	0.843	0.706	2.19E-106	Y		Y
TNFAIP2	1.99E-103	-6.09E-01	0.157	0.362	6.11E-99	Y		Y

Gene	p_val	avg_log2FC	pct.1	pct.2	p_val_adj	TNF-α[a]	EMT[b]	shared[c]
CXCL2	5.37E-99	1.68E+00	0.459	0.232	1.65E-94	Y		Y
IFNGR2	9.31E-99	3.82E-01	0.643	0.408	2.86E-94	Y		Y
BTG1	1.30E-93	4.49E-01	0.729	0.52	3.99E-89	Y		Y
NFIL3	1.64E-89	4.65E-01	0.518	0.281	5.05E-85	Y		Y
BHLHE40	4.82E-76	7.45E-01	0.632	0.444	1.48E-71	Y		Y
MSC	3.23E-73	3.21E-01	0.307	0.118	9.90E-69	Y		Y
IRS2	1.70E-69	-3.25E-01	0.139	0.301	5.20E-65	Y		Y
G0S2	3.50E-67	-5.72E-01	0.135	0.29	1.07E-62	Y		Y
TNFAIP8	2.25E-64	2.93E-01	0.274	0.103	6.90E-60	Y		Y
NINJ1	3.07E-62	2.83E-01	0.414	0.227	9.41E-58	Y		Y
IER3	2.84E-28	3.17E-01	0.559	0.441	8.70E-24	Y		Y
ATP2B1	3.38E-28	-3.26E-01	0.236	0.34	1.04E-23	Y		Y
DENND5A	1.07E-26	-3.64E-01	0.335	0.428	3.28E-22	Y		Y
MYC	1.98E-16	3.85E-01	0.436	0.346	6.07E-12	Y		Y
FOS	0	1.22E+00	0.974	0.894	0	Y		
ZFP36	0	1.73E+00	0.948	0.833	0	Y		
F3	4.60E-194	-6.70E-01	0.099	0.371	1.41E-189	Y		
GFPT2	1.73E-193	-1.00E+00	0.147	0.424	5.31E-189	Y		
ATF3	1.67E-174	1.24E+00	0.757	0.506	5.13E-170	Y		
KLF6	1.77E-145	8.77E-01	0.829	0.638	5.44E-141	Y		
EGR1	4.11E-134	7.25E-01	0.934	0.809	1.26E-129	Y		
NFE2L2	5.79E-132	-6.79E-01	0.742	0.806	1.78E-127	Y		
MARCKS	8.92E-107	4.64E-01	0.821	0.602	2.74E-102	Y		
KLF2	7.98E-105	9.43E-01	0.833	0.673	2.45E-100	Y		
CEBPD	6.01E-104	1.03E+00	0.865	0.793	1.84E-99	Y		
NFAT5	4.79E-98	-7.54E-01	0.41	0.578	1.47E-93	Y		
TUBB2A	2.21E-66	7.33E-01	0.438	0.264	6.77E-62	Y		
PER1	3.58E-63	-4.33E-01	0.329	0.492	1.10E-58	Y		
KLF4	3.01E-61	-6.21E-01	0.446	0.599	9.25E-57	Y		
GCH1	1.16E-60	-2.73E-01	0.045	0.149	3.55E-56	Y		
TANK	4.36E-59	-4.61E-01	0.286	0.444	1.34E-54	Y		
CFLAR	6.45E-57	-4.15E-01	0.331	0.487	1.98E-52	Y		
CCND1	3.71E-51	3.16E-01	0.548	0.371	1.14E-46	Y		
JAG1	1.30E-50	3.01E-01	0.537	0.364	3.98E-46	Y		
NR4A3	2.40E-49	-8.30E-01	0.179	0.309	7.38E-45	Y		
IRF1	6.28E-47	4.61E-01	0.495	0.329	1.92E-42	Y		
FOSB	6.45E-45	3.22E-01	0.883	0.728	1.98E-40	Y		
TRIB1	2.91E-40	3.40E-01	0.21	0.09	8.94E-36	Y		
SMAD3	1.45E-37	-4.72E-01	0.323	0.447	4.45E-33	Y		
KLF10	1.42E-36	2.93E-01	0.555	0.404	4.34E-32	Y		
B4GALT1	4.22E-35	-5.07E-01	0.406	0.503	1.29E-30	Y		
IL6ST	1.23E-34	-3.27E-01	0.78	0.792	3.78E-30	Y		
PDLIM5	1.64E-31	-5.86E-01	0.663	0.66	5.04E-27	Y		

Gene	p_val	avg_log2FC	pct.1	pct.2	p_val_adj	TNF-α[a]	EMT[b]	shared[c]
PLK2	8.34E-29	2.89E-01	0.334	0.217	2.56E-24	Y		
DRAM1	4.67E-26	-2.92E-01	0.173	0.267	1.43E-21	Y		
IER2	4.11E-25	4.20E-01	0.816	0.722	1.26E-20	Y		
ICAM1	5.08E-24	6.38E-01	0.4	0.302	1.56E-19	Y		
EGR3	1.33E-23	-3.37E-01	0.196	0.286	4.09E-19	Y		
CSF1	1.01E-20	-3.24E-01	0.308	0.393	3.09E-16	Y		
PLPP3	2.98E-15	-4.33E-01	0.631	0.666	9.14E-11	Y		
CCNL1	2.02E-11	-4.39E-01	0.646	0.652	6.19E-07	Y		

[a]DEGs enriched in TNF-α signaling via NF-κB pathway

[b]DEGs enriched in EMT pathway

[c]DEGs enriched in the above two pathways and shared with Fibroblast_c1-POSTN

Supplementary Table 4. Transcription factors predicted from 18 hub genes using TRRUST

ID	Key TF	Description	# of overlapped genes	P value	FDR
1	POU5F1	POU class 5 homeobox 1	3	1.21E-06	9.72E-06
2	POU2F1	POU class 2 homeobox 1	3	5.02E-06	2.01E-05
3	HOXD3	homeobox D3	2	7.63E-06	2.03E-05
4	PAX6	paired box 6	2	4.18E-05	8.36E-05
5	TWIST2	twist basic helix-loop-helix transcription factor 2	2	0.000226	0.000362
6	JUND	jun D proto-oncogene	2	0.000421	0.000562
7	CEBPA	CCAAT/enhancer binding protein (C/EBP), alpha	2	0.000949	0.00108
8	SP1	Sp1 transcription factor	3	0.00814	0.00814

Supplementary Table 5. Clinical characteristics of samples used for spatial transcriptomics and multiplex immunofluorescence

Subject ID	Group	Diagnosis	Gender	Age, years	Ethnicity	Hypertension	Pneumothorax	Family history	Assay
TAA-1	TAA	Thoracic aortic aneurysm	Male	30	Chinese	No	Yes	No	10X Visium spatial transcriptomics
TAA-2	TAA	Thoracic aortic aneurysm	Female	27	Chinese	No	No	Yes	10X Visium spatial transcriptomics
TAA-3	TAA	Thoracic aortic aneurysm	Male	62	Chinese	Yes	No	No	Multiplex immunofluorescence
Control-1	Control	Heart transplant recipient	Female	40	Chinese	No	No	No	10X Visium spatial transcriptomics
Control-2	Control	Heart transplant recipient	Male	53	Chinese	No	No	No	10X Visium spatial transcriptomics
Control-3	Control	Lower limb arterial thrombosis	Male	58	Chinese	No	No	No	Multiplex immunofluorescence
